# Diverse Approaches for the Difunctionalization of PPH Dendrimers, Precise Versus Stochastic: How Does this Influence Catalytic Performance?

**DOI:** 10.1021/acsmacrolett.4c00204

**Published:** 2024-06-25

**Authors:** Massimo Petriccone, Régis Laurent, Anne-Marie Caminade, Rosa María Sebastián

**Affiliations:** † Department of Chemistry, Science Faculty, Universitat Autònoma de Barcelona, Campus de Bellaterra, s/n, 08193 Cerdanyola del Vallès, Barcelona, Spain; ‡ Centro de Innovación en Química Avanzada (ORFEO−CINQA), Universitat Autònoma de Barcelona, Cerdanyola del Vallès, Bellaterra, 08193, Barcelona, Spain; § Laboratoire de Chimie de Coordination, CNRS, 205 Route de Narbonne, 31077 Toulouse, CEDEX 4, France; ∥ LCC−CNRS, Université de Toulouse, CNRS, 31077 Toulouse, France

## Abstract

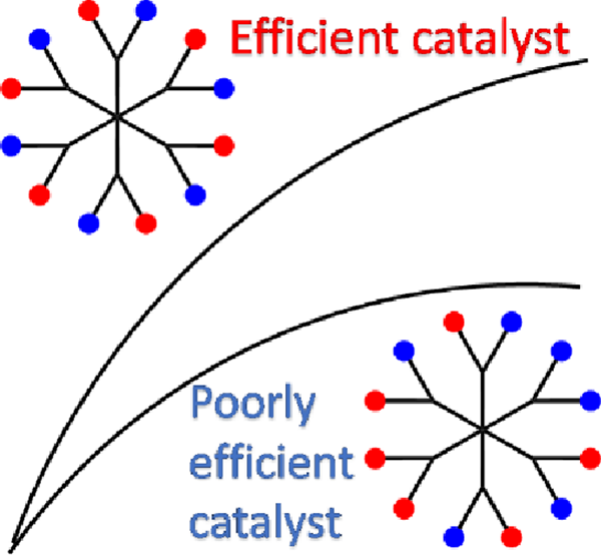

Random difunctionalization of dendrimer surfaces, frequently employed in biological applications, provides the advantage of dual functional groups through a synthetic pathway that is simpler compared to precise difunctionalization. However, is the random difunctionalization as efficient as the precise difunctionalization on the surface of dendrimers? This question is unanswered to date because most dendrimer families face challenges in achieving precise functionalization. Polyphosphorhydrazone (PPH) dendrimers present a unique opportunity to obtain precise difunctionalization at each terminal branching point. The work concerning catalysis we report with PPH dendrimers, whether precisely or randomly functionalized, addresses this question. Across PPH dendrimers, from generations 1 to 3, precise functionalization consistently outperforms random functionalization in terms of efficiency. This finding introduces a novel concept in dendrimer science, emphasizing the superiority of precise over random functionalization methodologies. Introducing a groundbreaking concept in the field of dendrimers.

Dendrimers are macromolecules synthesized step-by-step (generation after generation), to ensure a supposed perfect structure.^1^ Paradoxically, a random difunctionalization of the surface of dendrimers is frequently applied, thus affording “imperfect” nano-objects from “perfect” ones. The random difunctionalization is generally applied with the aim of affording two different properties to the dendrimers, for instance, one function for increasing the solubility in a given solvent such as water, the second affording the desired properties. The random difunctionalization is in particular used when studying biological properties of dendrimers.^2^ However, inconsistencies between batches have been already pointed out, leading to batches with varying biological activities.^3^ Meanwhile, to the best of our knowledge, no study up to now has compared the influence of random versus precise difunctionalization on all of the surfaces of dendrimers on a given property. In fact, such a comparison is not easy to carry out, as the precise difunctionalization of most types of dendrimers is not possible, except when using difunctionalized reagents, such as those based on triazine,^4^ or Janus-type dendrimers,^5^ or eventually after tedious purification by HPLC of one, two, or three functions on the surface of dendrimers.^6^ Interestingly, polyphosphorhydrazone (PPH) dendrimers^7^ possess the rare property of an easy sequential difunctionalization on one Cl, then on a second Cl, at each P(S)Cl_2_ terminal function.^8^

In this Letter we describe both the precise and random difunctionalization of PPH dendrimers, from generation 1 to generation 3, with a perfluoroalkyl chain and an iminophosphine, suitable for the complexation of palladium. Both families were then used in catalytic experiments for detecting their different or similar properties.

The PPH dendrimers were synthesized as described previously,^6^ to have P(S)Cl_2_ terminal functions at each generation, from generation 1 (6 P(S)Cl_2_) to generation 3 (24 P(S)Cl_2_). The ligand chosen for the catalytic experiments of type iminophosphine was previously used by us for complexing palladium on a monomer and a first generation dendrimer^9^ and was found efficient and usable in several catalytic experiments.^9,10^ The other function that we chose was a perfluoroalkyl chain, which could potentially modify the solubility of the PPH dendrimers.

The P(S)Cl_2_ functions of the PPH dendrimers react easily with phenols and amines. The easy and precise difunctionalization was previously observed essentially with amines.^8^ It was carried out only one time with two phenol derivatives,^11^ affording a difunctional dendrimer with a purity of ca. 95%. As in our experience, it is generally easier to functionalize the PPH dendrimers with phenols than with primary amines; we choose to use a phenol for both types of substituents. The perfluoroalkyl derivative **1** (HOAr_1_) was synthesized by a substitution reaction between 4-mercaptophenol and 1,1,1,2,2,3,3,4,4,5,5,6,6-tridecafluoro-8-iodooctane in the presence of triethylamine in refluxing THF overnight, instead of K_2_CO_3_ in refluxing acetone for 1 day.^12^ The ligand **2** (HOAr_2_) was synthesized as previously described^9^ by a condensation reaction between 2-(diphenylphosphino) benzaldehyde and 4-aminophenol.

Having both phenols in hand, the first step was to study their grafting on a model compound (**M**) bearing a single P(S)Cl_2_ function. It was decided to graft first phenol **1**, as it is a very stable compound, contrarily to phenol **2**, in which the phosphine can be easily oxidized. The monofunctionalized monomer **M-mono** was isolated and characterized, then phenol **2** bearing a phosphine was reacted to afford the difunctionalized monomer **3prc**. Both reactions were carried out under basic conditions, using cesium carbonate, whereas sodium sulfate was used as a drying agent. The monofunctionalization with phenol **1** was then attempted with generations 1 (**G1**), 2 (**G2**), and 3 (**G3**) of the PPH dendrimers in basic conditions. A very small proportion of unreacted P(S)Cl_2_ and of doubly reacted P(S)(OAr_1_)_2_ was observed in all cases by ^31^P{^1^H} NMR (less than 5%) (Scheme 1).

**Scheme 1:**
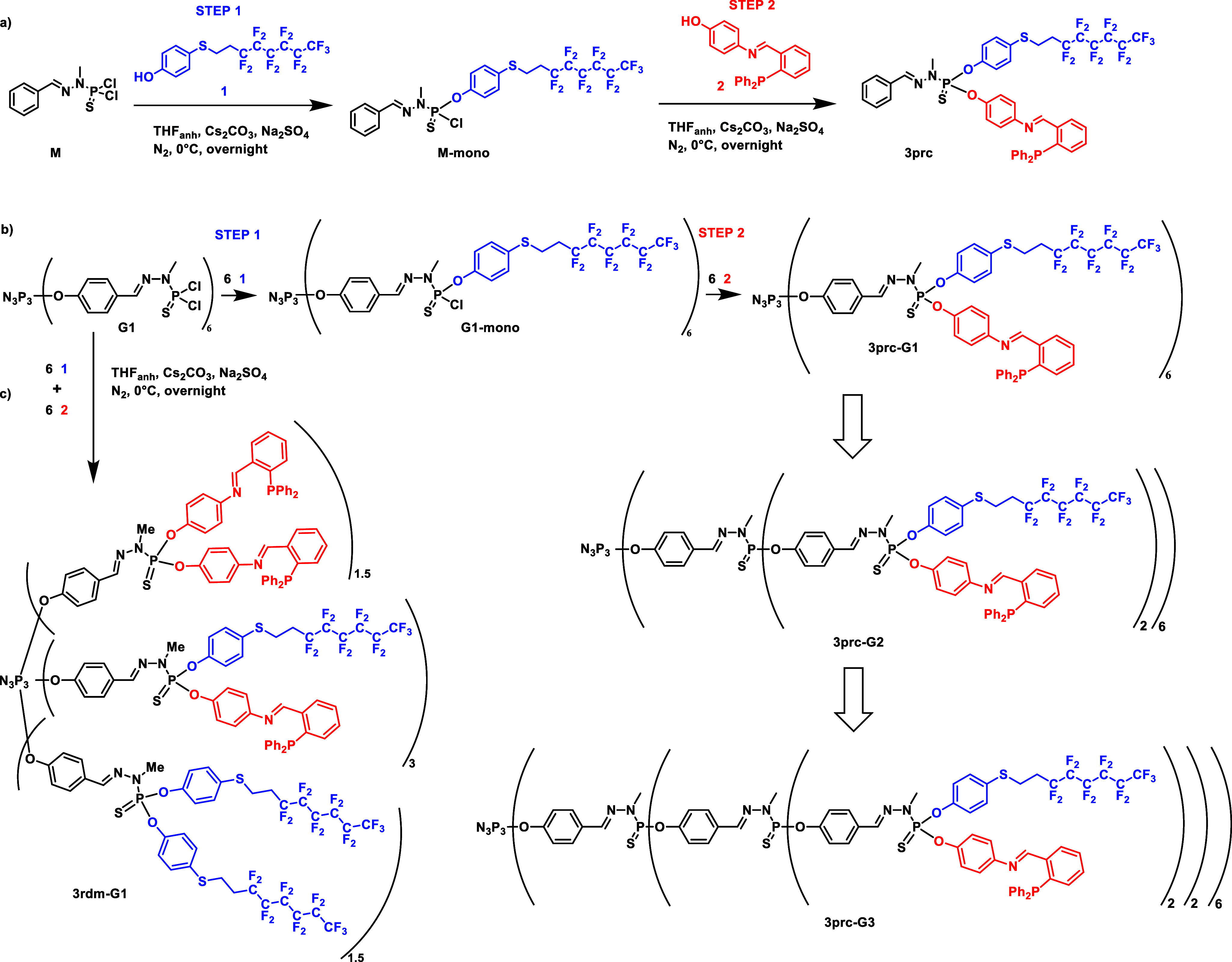
Synthesis of the (a) Precisely Difunctionalized Monomer (**3prc**) (b) of the First-Generation Dendrimer (**3prc-G1** Reaction Also Carried out to Synthesize **3prc-G2** and **3prc-G3**) (c) and of **3rdm-G1** (Reaction Also Carried out to Synthesize **3rdm-G2** and **3rdm-G3**); Structure of **3prc-G2** and **3prc-G3**

The main ^31^P{^1^H} NMR signal corresponding to the monosubstitution is observed at 68 ppm for all generations. The second phenol (**2**) was then reacted, also in the presence of cesium carbonate and sodium sulfate. After completion of this second reaction, dendrimers **3prc-Gn** (n = 1, 2, 3) (Scheme 1) were isolated, having less than 5% of symmetrical P(S)(OAr_1_)_2_ and P(S)(OAr_2_)_2_ terminal functions.

In the next experiment, generations 1, 2, and 3 of the PPH dendrimers were reacted simultaneously with both phenols **1** and **2** under basic conditions. As expected, this reaction provided randomly functionalized dendrimers **3rdm-Gn** (n = 1, 2, 3), in which ca. 50% of the terminal functions are of type P(S)(OAr_1_)(OAr_2_), ca. 25% of type P(S)(OAr_1_)_2_, and ca. 25% of type P(S)(OAr_2_)_2_. Scheme 1 illustrates the reaction for the first generation and the structure of **3rdm-G1**.

Figure 1 displays the ^31^P{^1^H} NMR spectra (only the part corresponding to the P=S groups) of both the **3prc-G1** and **3rdm-G1** dendrimers (only one of the numerous possible structures is shown for the latter), pointing to the large difference between both families of dendrimers. It should be emphasized that ^31^P NMR is a unique and very precious tool for the accurate characterization of dendrimers,^13^ in particular, of highly sophisticated dendritic structures.^14^ Indeed, neither ^1^H nor ^19^F NMR were found suitable to detect any difference between the families of random (**3rdm-Gn**) and precise (**3prc-Gn**) difunctionalization of the dendrimers surface. ^13^C{^1^H} NMR displays slight differences on some signals, but not as easily interpretable as ^31^P{^1^H} NMR spectra (see spectra in SI). Mass spectrometry is unusable, as cleavages and rearrangements are always observed with PPH dendrimers in MALDI-Tof experiments.^15^

**Figure 1:**
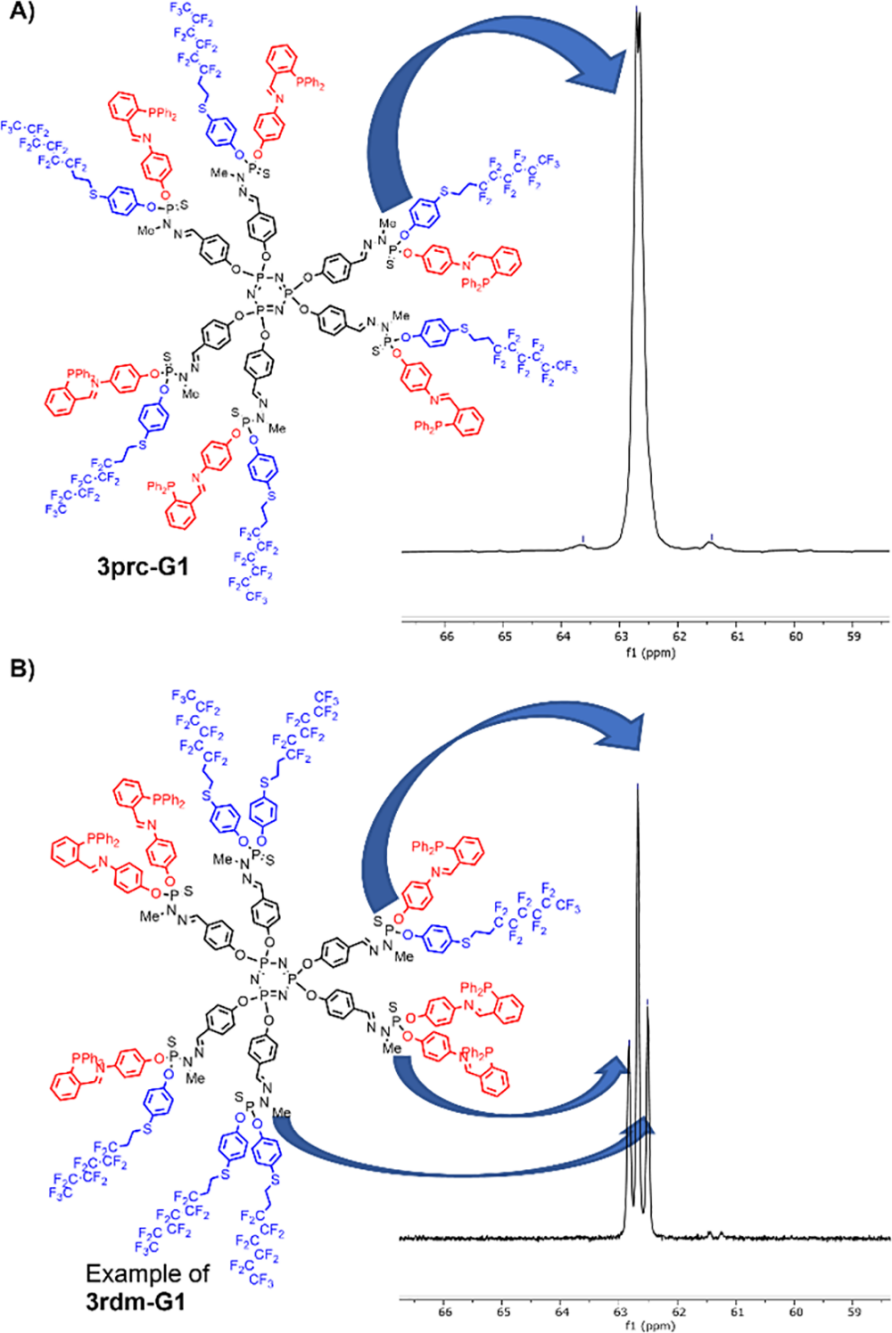
^31^P{^1^H} NMR spectra of the P(S) groups in **3prc-G1** (A) and **3rdm-G1** (B) dendrimers.

The complexation ability of the iminophosphine was checked on the model compound **3prc** and then on dendrimers **3prc-Gn** and **3rdm-Gn** (*n* = 1–3), respectively, using Pd(1,5-cyclooctadiene)Cl_2_ (Scheme 2). The complexation was, in particular, characterized by ^31^P{^1^H} NMR, which displayed the disappearance of the signal corresponding to the free phosphine at ca. δ = −13 ppm, on behalf of the appearance of the signal corresponding to the phosphine complex at ca. δ = 30.8 ppm. It can be noted that there is no control of the chirality on the P(S)(OAr_1_)(OAr_2_) surface groups, and thus, all catalytic tests were carried out with nonchiral ligands, reagents, and reactions.

**Scheme 2:**
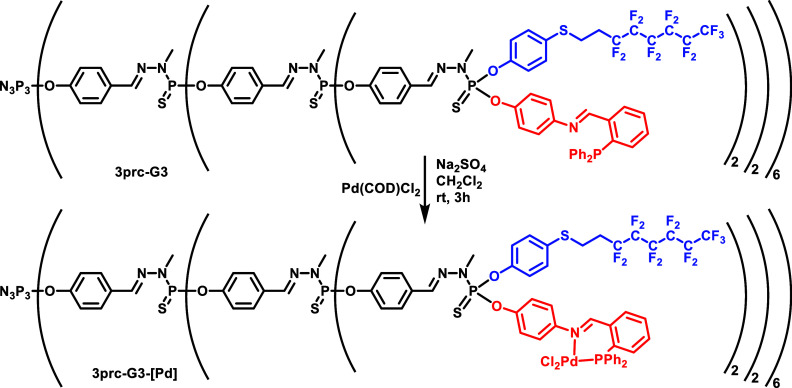
Example of the Synthesis of the Palladium Complex of the Dendrimer **3prc-G3** Reaction was also carried out with the monomer **3prc** and dendrimers **3prc-Gn** (*n* = 1, 2) and **3rdm-Gn** (*n* = 1–3).

Having in hand two families of dendrimer complexes, namely, precisely and randomly difunctionalized, from generations 1–3, they were then tested in the Stille coupling^16^ of iodobenzene with tributylvinyltin in THF at 50 °C. In order to have an accurate comparison between the different generations, the same quantity of catalytic sites was used in all cases. For instance, the efficiency of 1 equiv of generation 3 having 24 ligands complexing Pd is compared with that of 4 equiv of generation 1 having 6 ligands complexing Pd, and with that of 24 equiv of the monomeric complex. Even if the distance between the catalytic sites and their accessibility changes with the generations, this is the only way to compare the efficiency between generations. Figure 2 displays the results of these catalytic experiments monitored by NMR for 7 h. All experiments were carried out in duplicate, and the uncertainty is given in Figure 2. The precisely functionalized dendrimers display a slightly positive dendritic effect on going from generation 1 to generation 3.^17^ An analogous, slightly positive dendritic effect is observed with the randomly functionalized dendrimers. However, it is important to note that in all cases from generation 1 to generation 3 the precisely difunctionalized dendrimers **3prc-Gn-[Pd]** are more efficient catalysts than the randomly functionalized dendrimers **3rdm-Gn-[Pd]**. The difference is most accurate between the first generations **3prc-G1-[Pd]** and **3rdm-G1-[Pd]**, but it is still large with generations 2 and 3.

**Figure 2:**
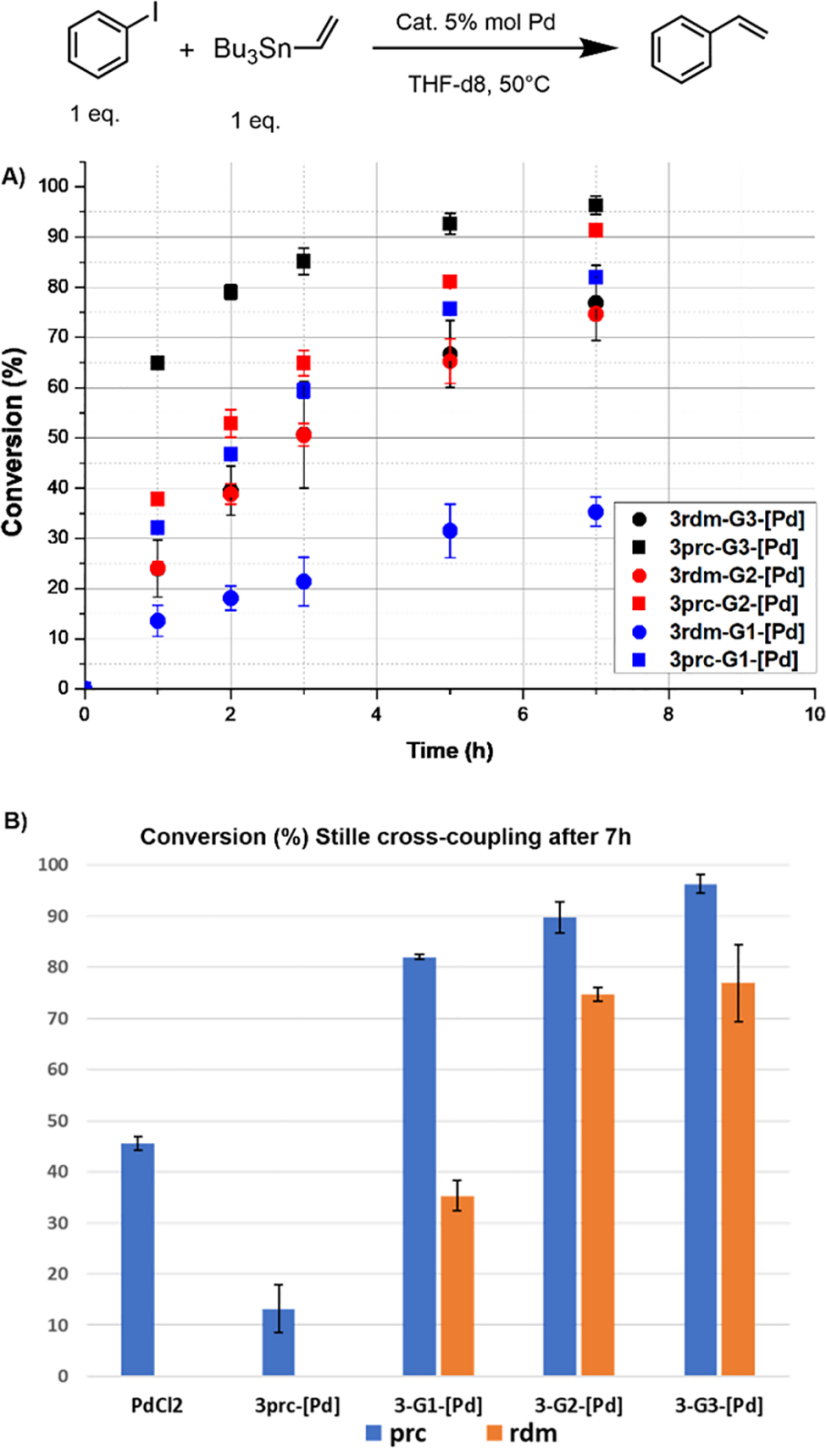
Stille cross-coupling catalytic experiments with precisely and randomly difunctionalized dendrimers from generation 1 to generation 3 and monomers: (A) evolution with time; (B) comparison of efficiency after 7 h.

Part B of Figure 2 displays the results after 7 h, with additional information about the use of PdCl_2_ alone and of the monomer. There is a large difference in the efficiency between monomer **3prc-[Pd]**, which is less efficient than PdCl_2_ alone, and the different generations of the dendrimers, illustrating again a dendritic effect. As we had previously demonstrated a different efficiency of the Pd-iminophosphine catalysts, depending on the type of Stille couplings carried out,^9^ we decided to test **3prc-G1-[Pd]** and **3rdm-G1-[Pd]** in another type of Stille couplings. The reagents used in this case were methyl-2-iodobenzoate and 2-(tributylstannyl) thiophene, and the catalysis was carried out for 22 h at 50 °C in THF. As shown in Figure 3, in this case also the precisely difunctionalized dendrimer **3prc-G1-[Pd]** was more efficient than the randomly difunctionalized dendrimer **3rdm-G1-[Pd]**.

**Figure 3:**
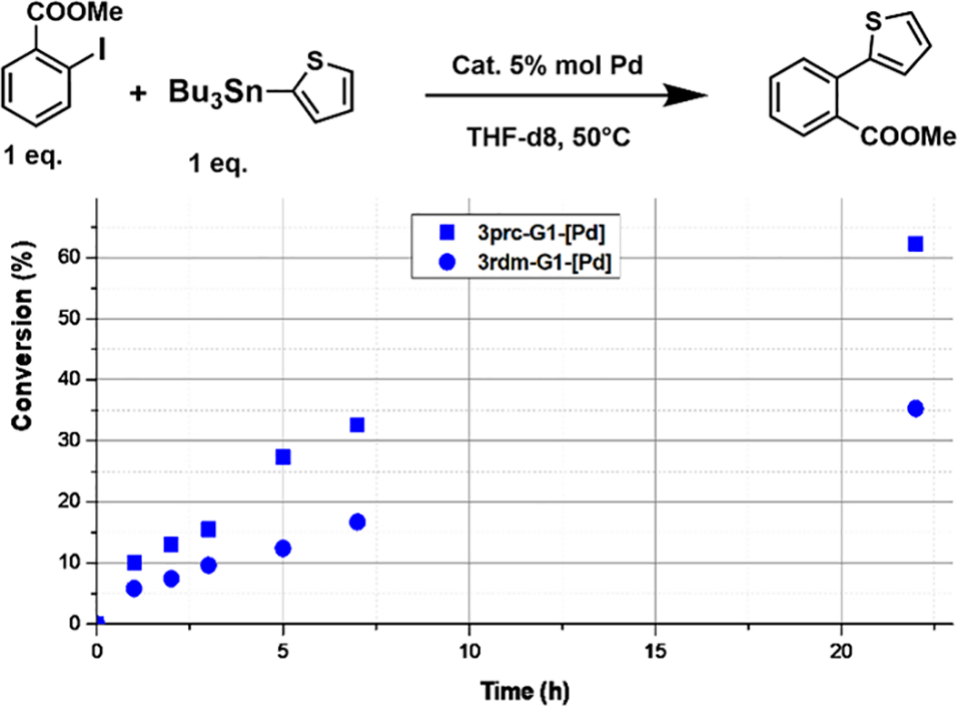
Another example of Stille coupling with precisely and randomly functionalized dendrimer **3-G1-[Pd]**.

In order to confirm more (or not) the large difference in efficiency between random and precisely difunctionalized dendrimers observed in the catalysis of Stille couplings, the model **3prc-[Pd]** and the first generations **3prc-G1-[Pd]** and **3rdm-G1-[Pd]** were tested in another type of cross-coupling reactions, the Heck reaction.^18^ The reagents used were iodobenzene and methyl acrylate, in the presence of NEt_3_.^18^ It was shown previously that the presence of triethylamine increases the selectivity of the Heck coupling product.^19^ In our case, we observed 100% selectivity in the *trans* product.

As the reaction occurs more slowly than the previous ones, experiments were carried out for a longer time, 30 h. A weak dendritic effect was observed on going from monomer **3prc-[Pd]** to **3prc-G1-[Pd]**. In this case as previously, the precisely difunctionalized dendrimer **3prc-G1-[Pd]** was found more efficient than the random difunctionalized dendrimer **3rdm-G1-[Pd]**, as illustrated in Figure 4.

**Figure 4:**
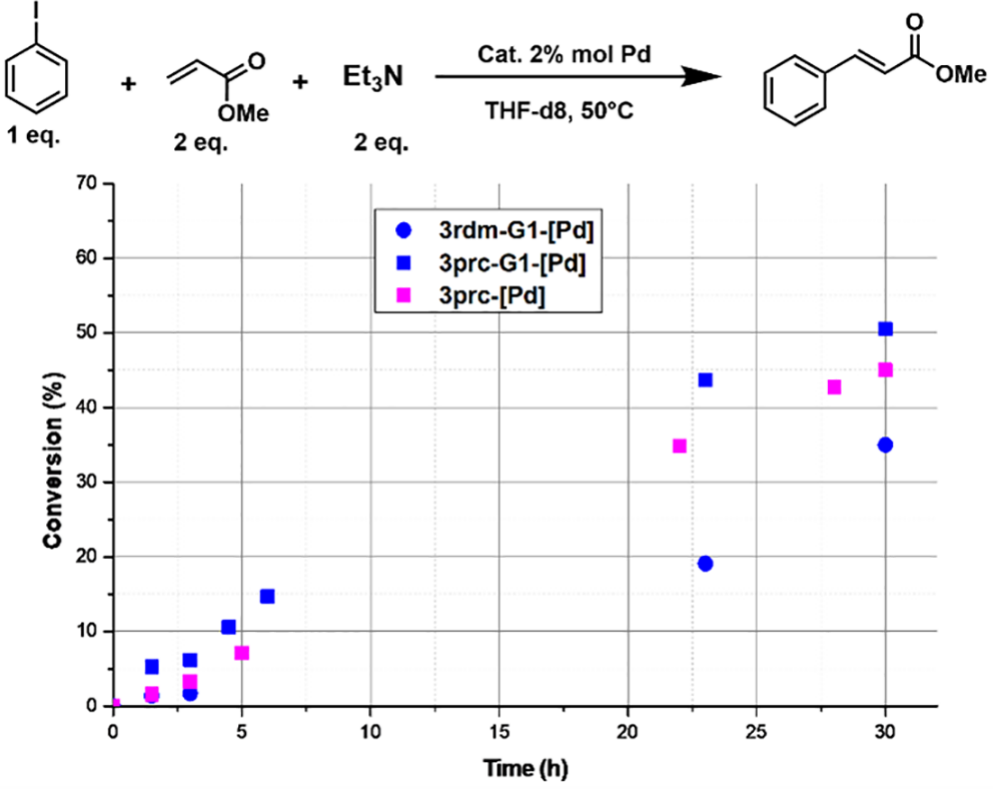
Heck cross-coupling reaction catalyzed by the monomer **3prc-[Pd]** and both dendrimers **3prc-G1-[Pd]** and **3rdm-G1-[Pd]**.

In this paper, we have demonstrated for the first time that the precise difunctionalization on all of the surface of dendrimers affords different results than their random difunctionalization. We have shown that the precise functionalization of PPH dendrimers is more efficient in two types of cross-coupling catalytic reactions. The difference is clearly visible for all generations, from the first to the third. Thus, besides the problems of reproducibility between batches previously known,^3^ the difference in efficiency between precisely and randomly difunctionalized dendrimers must be taken into account and should be checked in each case. Indeed, examples in which randomly functionalized dendrimers are more efficient than precisely functionalized dendrimers can probably also exist. We believe that this original finding is not limited to catalysis, but can be considered as a global warning when using randomly functionalized dendrimers in any field, such as in biology where it is widely used with only very few questions.^20^

## Supporting Information Available

Materials, characterization methods, and experimental section; ^31^P{^1^H} NMR, ^1^H NMR, ^13^C{^1^H} NMR, and ^19^F{^1^H} NMR, IR(ATR), and MS spectra of new products; general procedures for catalysis (PDF)
