# Supplementary material for: Diverse Approaches for the Difunctionalization of PPH Dendrimers, Precise Versus Stochastic: How Does this Influence Catalytic Performance?
Source: ACS Macro Lett. 2024 Jun 25;13(7):853–8. doi: 10.1021/acsmacrolett.4c00204 (PMC11256758; doi:10.1021/acsmacrolett.4c00204)
Supplement: Supplementary file 1 — mz4c00204_si_001.pdf [file mz4c00204_si_001.pdf]

Supporting Information

Diverse Approaches for the Difunctionalization of PPH Dendrimers. Precise Versus Stochastic: How Does this Influence Catalytic Performance?

Massimo Petriccone,[a,b,c,d] Régis Laurent,[c,d] Anne-Marie Caminade,*[c,d] and Rosa Maria Sebastián,*[a,b]

[a] Department of Chemistry, Science Faculty, Universitat Autònoma de Barcelona, Campus de Bellaterra, s/n, 08193 Cerdanyola del Vallès, Barcelona, Spain.

[b] Centro de Innovación en Química Avanzada (ORFEO-CINQA), Universitat Autònoma de Barcelona, Cerdanyola del Vallès, Bellaterra, 08193, Barcelona, Spain.

[c] Laboratoire de Chimie de Coordination, CNRS, 205 Route de Narbonne, 31077 Toulouse, CEDEX 4, France.

[d] LCC-CNRS, Université de Toulouse, CNRS, 31077 Toulouse, France

Corresponding Author

* Prof. Dr. R. M. Sebastián
E-mail: rosamaria.sebastian@uab.cat

* Dr A.-M. Caminade
E-mail: anne-marie.caminade@lcc-toulouse.fr

1 Experimental **2**

1.1 Materials and methods **2**

1.2 General procedures **2**

1.2.1 Synthesis of 3prc-[Pd] **3**
1.2.2 Synthesis of 3prc-G1-[Pd] **4**
1.2.3 Synthesis of 3rdm-G1-[Pd] **6**
1.2.4 Synthesis of 3prc-G2-[Pd] **7**
1.2.5 Synthesis of 3rdm-G2-[Pd] **8**
1.2.6 Synthesis of 3prc-G3-[Pd] **9**
1.2.7 Synthesis of 3rdm-G3-[Pd] **10**

1.3 General procedure for catalysis **11**

1.3.1 Stille cross-coupling **11**
1.3.2 Heck cross-coupling **11**

1.4 NMR Spectra **12**

1.5 IR Spectra **31**

1.6 MS Spectra **35**

1 Experimental

1.1 Materials and methods

Analytical thin layer chromatography (TLC) was performed on Merck silica gel 60 F254 pre-coated aluminium sheets (0.20mm thickness) that were visualized by observation under UV light (254nm or 365nm). Flash column chromatography was performed using silica gel 60 (230-400 mesh) purchased from Merck. Melting points (M.p.) were recorded using a Kofler Reichert apparatus and are uncorrected.

NMR were done on BRUKER 300 MHz – 400 MHz Ascend™ AVANCE NEO console, BRUKER 400 MHz AVANCE III console, BRUKER 500 MHz UltraShield™ AVANCE NEO console,

^{1}H-NMR spectra were recorded at room temperature. Chemical shifts (δ) are reported in parts per million (ppm) downfield from trimethyl silane (TMS) and referred to residual solvent peak: CDCl3 (δH = 7.26 ppm). The following abbreviations are used to indicate the multiplicity signal: s (singlet), d (doublet), t (triplet), q (quartet), quin (quintet), sept (septet), dd (doublet of doublets), dt (doublet of triplets), ddd (doublet of doublets), br s (broad singlet), m (multiplet), 2 x s (two singlets), 2 x d (two doublets). The spectra were processed using MestRerNova software.

^{13}C-NMR spectra were recorded at room temperature. Chemical shifts are reported in parts per million downfield from TMS signal and referred to residual peak solvent: $CDCl_3$ (δC = 77.2 ppm). Otherwise stated, the multiplicity refers to ^{1}H decoupled spectra. The spectra were processed using MestReNova software.

^{19}F-NMR spectra were recorded at room temperature. Chemical shifts are reported in parts per million downfield from $CFCl_3$ signal at 0.00 ppm as external standard. The spectra were processed using MestReNova software.

^{31}P-NMR spectra were recorded at room temperature in $CDCl_3$. Chemical shifts are reported in parts per million relative to H_3PO_4 85% aqueous solution (δP = 0.00 ppm) as an external standard. The spectra were processed using MestReNova software.

IR spectra were recorded with on a Bruker Tensor 27 spectrometer fitted with a universal Attenuated Total Reflectance Golden Gate module.

Determination of percentage of palladium (ICP-OES) was measured at the Servei d'Anàlisi Química de l'Universitat Autònoma de Barcelona.

1.2 General procedures

Unless otherwise stated, all reagents were purchased from commercial sources and used without further purification, except for:

- 4-hydroxibenzaldehyde
- Phosphorothioyl trichloride
- Hexachlorophosphazene
- (*E*)-4-((2-(diphenylphosphaneyl)benzylidene)amino)phenol (**2**) [8]
- 4-(2-perfluorohexyl)ethylthiolphenol (**1**) [11]
- (1-methylhydrazineyl)phosphonothioic dichloride [6]
- (*E*)-(2-benzylidene-1-methylhydrazineyl)phosphonothioic dichloride (**M**) [19]

- Phosphorhydrazone dendrimers from G1 to G3 [6]

Which where synthesised according to literature procedures. All reactions were carried out under an atmosphere of nitrogen using standard Schlenk line techniques unless stated otherwise. Dry, oxygen-free solvents (THF and CH_2Cl_2) were obtained from an Innovative technology PureSolv-MD-2 solvent purification system and directly stored under 4 Å. Other solvents (Et_2O and n-pentane) were distilled and degassed by freeze-pump-thaw technique before use. Abbreviations: tetrahydrofuran (THF), deuterated tetrahydrofuran (THF-*d8*).

1.2.1 Synthesis of 3prc-[Pd]

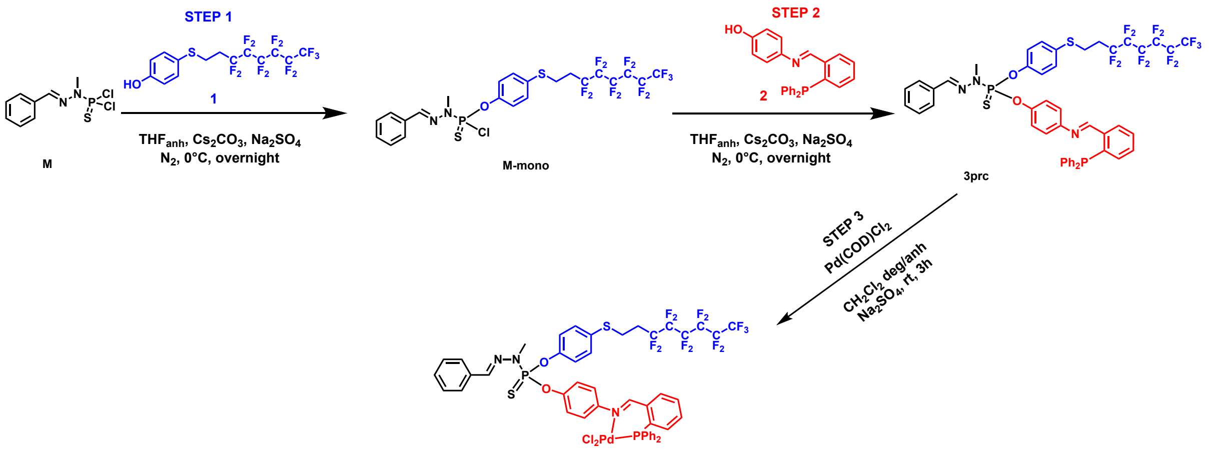

STEP1: in a Schlenk flask, equipped with an addition funnel, Cs_2CO_3 (0.61 g, 1,9 mmol), Na_2SO_4 (0.40 g, 2.8 mmol) and M (0.40 g, 1,4 mmol) were dried them under vacuum for 30 minutes, then under inert atmosphere was added degassed anhydrous THF (15ml), then under stirring, the system was placed in an ice bath. In a round bottomed flask **1** (0.64 g, 1,4 mmol) was dried under vacuum, then under inert atmosphere it was dissolved in degassed anhydrous THF (8 ml). This solution was transferred in the addition funnel and was added to the reaction solution dropwise with a rate of a drop every 8/9 sec. The reaction solution was left reaching r.t. under stirring overnight. The reaction was checked through ^{31}P-NMR, when the mono-substitution is complete (signal at 68.5 ppm), was possible to proceed the **STEP2** for the one pot reaction.

^{31}P{^{1}H}-NMR (162 MHz, $CDCl_3$) δ(ppm): 68.5, 62.3, 62.3.

STEP2: Cs_2CO_3 (0.94 g, 2,9 mmol) was added to the reaction solution and it was degas again fluxing N_2. In a round bottomed flask **2** (0.54 g, 1.4 mmol) was dried under vacuum, then under inert atmosphere was dissolved in degassed anhydrous THF (6 ml). This solution was added to the reaction mixture and was left under stirring at r.t. overnight. The reaction was checked through ^{31}P-NMR, when the signal of the mono substituted product disappeared (68.5 ppm), the reaction was stopped. The solution was filtered from the salt, then the solvent was evaporated obtaining a yellow sticky soft solid as crude mixture.

^{31}P{^{1}H}-NMR (162 MHz, $CDCl_3$) δ(ppm): 62.8, 62.5, 62.3, -13.3, -13.3, -13.6.

STEP3: in a Schlenk flask, the crude mixture from the **STEP2** (1,24 g, 1,2 mmol, moles were calculated doing an estimation of impurities through the NMR), Na_2SO_4 (0.60 g, 4.2 mmol) and Pd(1,5-cyclooctadiene)Cl_2 (0.34 g, 1.1) were dried under vacuum, then under inert atmosphere was added degassed anhydrous CH_2Cl_2 (50 ml) and the stirring was started. The reaction was checked through ^{31}P-NMR after 3h (disappearing of the signal at -13.3 ppm). If the complexation

was not complete was added a little amount of Pd(1,5-cyclooctadiene)Cl_2. The solution was filtered from the salt, then the volume of the solution was reduced to 2-3 ml. This solution was dropped in pentane/Et_2O 4:1 (70 ml) under hard stirring, the solution from the precipitate, the solid was washed 2x with the same mixture of solvents. The product was obtained as an orange solid (1.32 g, 1.0 mmol). **Yield = 73%**

M.p.: 147°C. **Chemical Formula:** $C_{47}H_{36}Cl_2F_{13}N_3O_2P_2PdS_2$. **^{1}H-NMR** (400 MHz, $CDCl_3$) δ(ppm): 8.19 (s, 1H, CH=N), 7.98 – 7.90 (m, 1H), 7.80 – 7.70 (m, 3H), 7.67 (s, 1H), 7.63 – 7.11 (m, 22H), 7.03 (dd, J_1 = 10.6, J_2 = 7.5 Hz, 1H), 3.33 (d, J = 10.6 Hz, 3H), 3.14 – 3.03 (m, 2H, CH_2-S), 2.47 – 2.29 (m, 2H, CH_2-CF_2). **^{13}C{^{1}H}- NMR** (101 MHz, $CDCl_3$) δ(ppm): 165.5 (d, J = 8.7 Hz), 157.6, 149.9 (t, J = 5.8 Hz), 148.9 (d, J = 2.3 Hz), 140.7 (d, J = 13.8 Hz), 137.2 (d, J = 8.5 Hz), 136.7 (d, J = 14.9 Hz), 134.7, 134.4 (d, J = 7.9 Hz), 134.2 (d, J = 11.3 Hz), 133.8 (d, J = 3.1 Hz), 132.6 (d, J = 3.1 Hz), 131.8 (d, J = 1.6 Hz), 131.0 (d, J = 2.2 Hz), 129.5, 129.1 (d, J = 1.5 Hz), 128.8, 127.2, 125.3, 124.8, 124.6, 122.5 (d, J = 4.9 Hz), 122.4, 121.90, 121.7 (d, J = 4.8 Hz), 122.6 – 105.9 (complex pattern due to C-F coupling), 33.2 (d, J = 12.6 Hz, CH_2-S), 31.6 (t, J = 22.1 Hz, CH_3-N), 25.5 (t, J = 4.4 Hz, CH_2-CF_2). **^{19}F NMR** (377 MHz, $CDCl_3$) δ(ppm): δ -80.8 (t, J = 9.8 Hz), -114.1, -121.9, -122.8, -123.2, -126.1. **^{31}P{^{1}H}-NMR** (162 MHz, $CDCl_3$) δ(ppm): 62.5, 30.8. **IR (neat) ν (cm^{-1}):** 1488, 1235, 1195, 1143, 913, 692. **[M+Na]$^+$ calculated** = 1245.9821; **[M+Na]$^+$ measured** = 1245.9865. **ICP-OS: Pd(%(p/p))** = 7.9 ± 1.5%rsd; **Calculated Pd(%(p/p))** = 8.7.

1.2.2 Synthesis of 3prc-G1-[Pd]

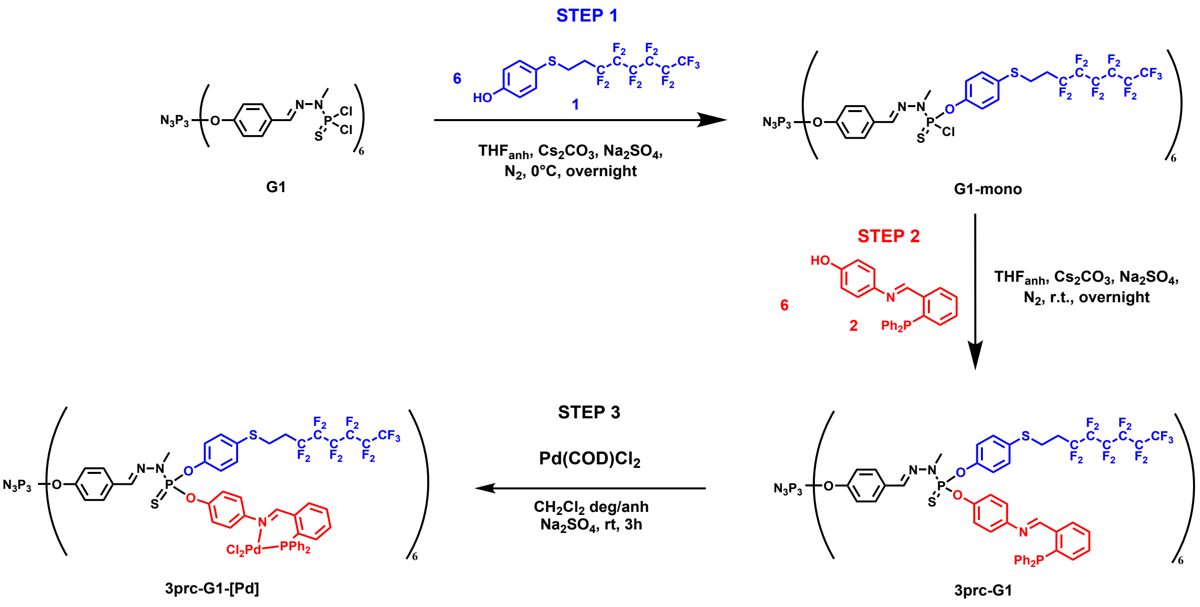

STEP1: in a Schlenk, equipped with an addition funnel, Cs_2CO_3 (1.26 g, 3.8 mmol), Na_2SO_4 (0.60 g, 4.2 mmol)and Gc_1 (0.90 g, 0.5 mmol) were dried under vacuum for 30 minutes. Then under inert atmosphere, degassed anhydrous THF was added (25 ml) and under stirring, the system was place in an ice bath. In a round bottomed flask, **1** (1.34 g, 2.8 mmol) was dried under vacuum, then under inert atmosphere was dissolved in degassed anhydrous THF (18 ml). This solution was transferred in the addition funnel and was added to the reaction mixture dropwise with a rate of a drop every 8/9 sec. The reaction solution was left under stirring overnight letting it reach room temperature. The reaction was checked through ^{31}P-NMR, when the mono-substitution was complete (signal at 68.0 ppm 31P-NMR), it was possible to proceed with the STEP2 in the one pot reaction.

$^{31}P\{^1H\}$-NMR (162 MHz, $CDCl_3$) δ(ppm): 68.0, 62.4, 62.3, 8.3.

STEP2: Cs_2CO_3 (2.09 g, 6.36 mmol) was added to the reaction solution, then it was degassed again. In a round bottomed flask, 2 (1.06 g, 2.8 mmol) was dried under vacuum, then under inert atmosphere was dissolved in degassed anhydrous THF (10 ml). This solution was added to the reaction mixture and was left under stirring at r.t. overnight. The reaction was checked through ^{31}P-NMR, when the signal of the mono substituted product was disappeared (68.0 ppm), the reaction was stopped. The solution was filtered from the salt, the volume of the solution was reduced to 2-3 ml and was dropped in degassed pentane (30 ml) under hard stirring to precipitate the product, the solution was filtered from the precipitate, the solid was dried under vacuum obtaining a yellow sticky soft solid as crude mixture (3.01 g, 0.5 mmol, moles were calculated doing an estimation of impurities through the NMR).

$^{31}P\{^1H\}$-NMR (162 MHz, $CDCl_3$) δ(ppm): 63.0, 62.8, 62.5, 8.3, -13.3.

STEP3: in a Schlenk Na_2SO_4 (0.60 g, 4.2 mmol), the crude mixture from the STEP2 (3.01 g, 0.5 mmol, moles were calculated doing an estimation of impurities through the NMR) and Pd(1,5-cyclooctadiene)Cl_2 (0.78 g, 2.7 mmol) were dried under vacuum, then under inert atmosphere degassed anhydrous CH_2Cl_2 was added (50 ml) and then the stirring was started. The reaction was checked through ^{31}P-NMR after 3h. If the complexation was not complete was added a little amount of Pd(1,5-cyclooctadiene)Cl_2. The solution was filtered from the salt, then the volume of the solution was reduced to 2-3 ml and was dropped in pentane/Et_2O 4:1 (70 ml) under hard stirring to precipitate the product. The precipitate was filtered and was dried under vacuum. The product was obtained as a light orange solid (3.50, 0.5 mmol). **Yield = 94%**

M.p.: >230°C. **Chemical Formula:** $C_{282}H_{210}Cl_{12}F_{78}N_{21}O_{18}P_{15}Pd_6S_{12}$. **Molecular Weight (theoretical):** 7576.03 g/mol. **^1H-NMR** (400 MHz, $CDCl_3$) δ(ppm): 8.22 (s, 6H, CH=N), 8.01 (s, 6H), 7.80 – 6.86 (complex absorption, 156H), 3.27 (d, J = 8.6 Hz, 18H), 3.05 (t, J = 8.1 Hz, 12H, CH_2-S), 2.46 – 2.23 (m, 12H, CH_2-CF_2). **$^{13}C\{^1H\}$-NMR** (101 MHz, $CDCl_3$) δ(ppm): 165.9, 151.2, 149.7, 148.0, 140.1, 137.7, 136.7, 134.1, 133.7, 133.4, 132.8 – 131.8 (complex absorption), 131.5, 131.3, 129.6 – 128.3 (complex absorption), 125.4, 124.6, 122.4, 121.4, 121.4-106.3 (complex pattern due to C-F coupling), 33.5 (CH_2-S), 31.5 (t, J = 22.3 Hz, CH_3-N), 25.4 (CH_2-CF_2). **^{19}F-NMR** (377 MHz, CDCl3) δ(ppm): -80.8 (t, J = 9.8 Hz), -114.1, -121.9, -122.9, -123.3, -126.2. **$^{31}P\{^1H\}$-NMR** (162 MHz, $CDCl_3$) δ(ppm): 62.8, 30.8, 8.4. **IR (neat) ν (cm^{-1}):** 1488, 1196, 1014, 943, 791. **ICP-OS: Pd(%(p/p)) =** 6.9 ± 0.5%rsd; **Calculated Pd(%(p/p)) =** 8.4.

1.2.3 Synthesis of 3rdm-G1-[Pd]

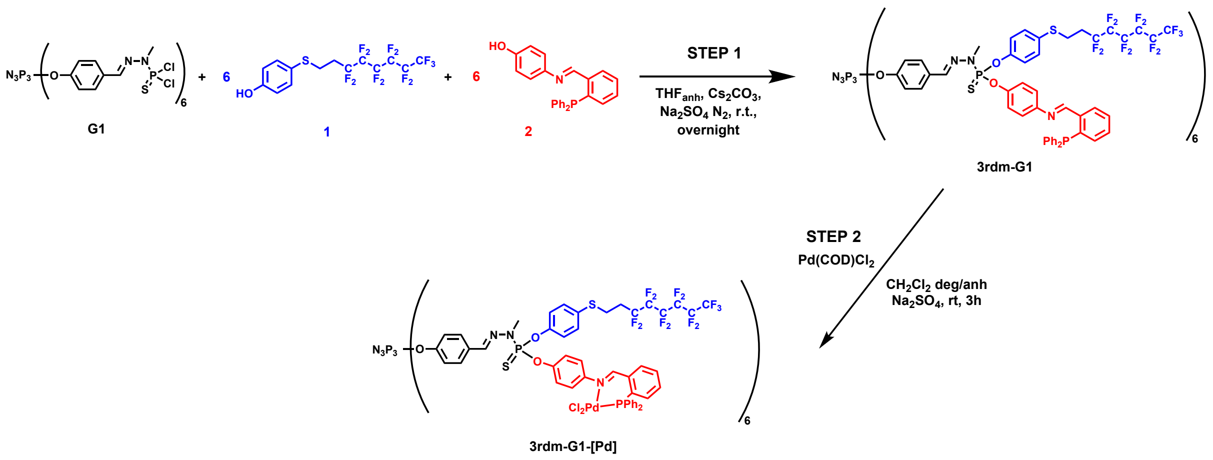

STEP1: in a Schlenk, equipped with an addition funnel, Cs_2CO_3 (0.90 g, 2.8 mmol) Na_2SO_4 (0.40 g, 2.8 mmol), **Gc_1** (0.20 g, 0.1 mmol), **1** (0.31 g, 0.7 mmol) and **2** (0.25 g, 0.65 mmol) were dried under vacuum for 30 minutes. Then under inert atmosphere anhydrous THF degassed (12 ml) was added and the reaction mixture was stirred at r.t. overnight. The reaction was checked through ^{31}P-NMR, when the reaction was completed with the disappearance of the signal of the starting material (3 signals of three different substitutions 1-1 62.4 ppm, 1-2 62.7 ppm, 2-2 62.9ppm) was stopped. The solution was filtered from the salt, the volume of the solution was reduced to 2-3 ml and was dropped in degassed pentane (30 ml) under hard stirring to precipitate the product, the precipitate was filtered and was dried under vacuum obtaining a yellow sticky soft solid as crude mixture (0.54 g, 0.1 mmol, moles were calculated doing an estimation of impurities through the NMR).

^{31}P{^{1}H}-NMR (162 MHz, $CDCl_3$) δ(ppm): 62.9, 62.7, 62.5, 8.3, -13.3.

STEP2: in a Schlenk Na_2SO_4 (0.10 g, 1.4 mmol), the crude mixture from the STEP1 (0.54 g, 0.1 mmol, moles were calculated doing an estimation of impurities through the NMR) and Pd(1,5-cyclooctadiene)Cl_2 (0.14 g, 0.4 mmol) were dried under vacuum, then under inert atmosphere degassed anhydrous CH_2Cl_2 (10 ml) was added and stirred. The reaction mixture was checked through ^{31}P-NMR after 3h (disappearance of the signal at -13.3 ppm). If the complexation was not complete was added a little amount of Pd(1,5-cyclooctadiene)Cl_2. The solution was filtered from the salt, the volume of the solution was reduced to 2-3 ml and was dropped in pentane/Et_2O 4:1 (50 ml) under hard stirring to precipitate the product. The solution was filtered from the precipitate, the solid was dried under vacuum, obtaining the product as a light orange solid (0.54 g, 0.1 mmol). **Yield = 66%**

M.p.: >230°C. **Chemical Formula:** $C_{282}H_{210}Cl_{12}F_{78}N_{21}O_{18}P_{15}Pd_6S_{12}$. **Molecular Weight (theoretical):** 7576.03 g/mol. **^{1}H-NMR** (400 MHz, $CDCl_3$) δ(ppm): 8.25 (s, 6H, CH=N), 8.03 (s, 6H), 7.85 – 6.65 (m, 156H), 3.29 (d, *J* = 18.5 Hz, 18H), 3.05 (m, 12H, CH_2-S), 2.44 – 2.21 (m, 12H, CH_2-CF_2). **^{13}C{^{1}H}-NMR** (101 MHz, $CDCl_3$) δ(ppm): 166.1, 151.2, 149.7, 149.0, 140.1, 137.7, 136.7, 134.0, 133.6, 132.6, 132.1, 131.6, 131.3, 129.1, 128.7, 125.4, 124.7, 122.4, 121.4, 121.4-106.3 (complex pattern due to C-F coupling), 33.6 (CH_2-S), 31.4 (t, *J* = 22.8 Hz), 25.4 (CH_2-CF_2). **^{19}F-NMR** (376 MHz, $CDCl_3$) δ(ppm): -80.9, -114.1, -122.0, -122.9, -123.3, -126.2. **^{31}P{^{1}H}-NMR** (162 MHz, $CDCl_3$) δ(ppm): 62.7, 62.2, 30.9, 8.4. **IR (neat) ν (cm^{-1}):** 3055, 1605, 1489,1234 1179, 1159, 1141, 940, 912, 744. **ICP-OS: Pd(%(p/p)) =** 7.6 ± 0.2%rsd; **Calculated Pd(%(p/p)) =** 8.4

1.2.4 Synthesis of 3prc-G2-[Pd]

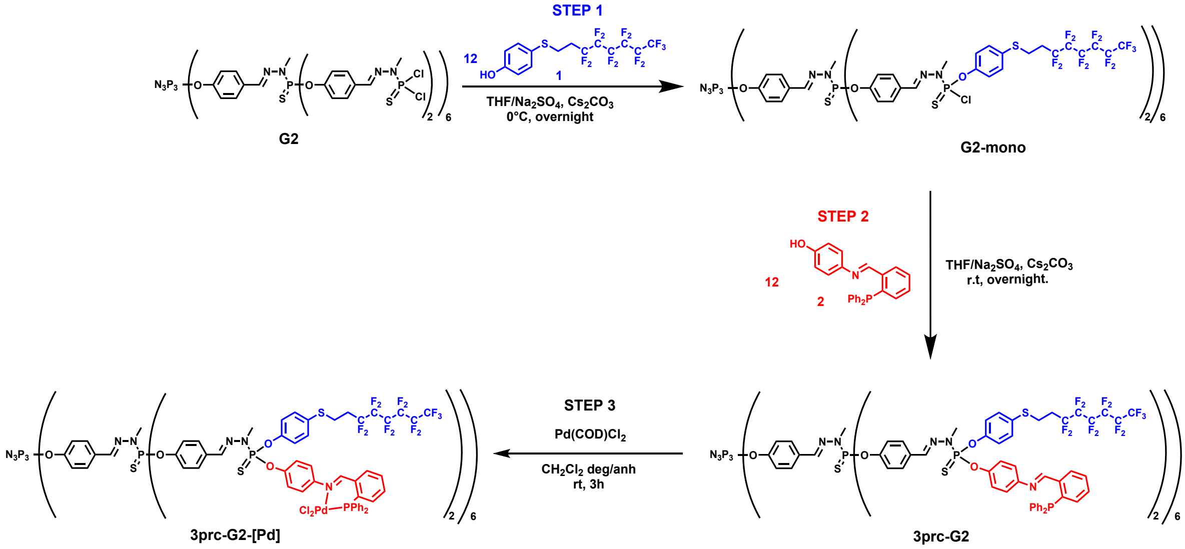

STEP1: in a Schlenk, equipped with an addition funnel, Cs_2CO_3 (0.76 g, 2.3 mmol), Na_2SO_4 (0.60 g, 4.2 mmol) and Gc_2 (0.50 g, 0.1 mmol) were dried under vacuum for 30 minutes. Then under inert atmosphere, degassed anhydrous THF (20 ml) was added and stirred, the system was place in an ice bath. In a round bottomed flask, **1** (0.55 g, 1.1 mmol) was dried under vacuum, then under inert atmosphere was dissolved in degassed anhydrous THF (15 ml). This solution was transferred in the addition funnel and was added to the reaction mixture dropwise with a rate of a drop every 8/9 sec. The reaction mixture was left under stirring overnight. The reaction was checked through ^{31}P-NMR and ^{1}H-NMR, when the mono-substitution was complete, it was possible to proceed with the STEP2 in one pot reaction.

^{31}P{^{1}H}-NMR (400 MHz, $CDCl_3$) δ(ppm): 68.3, 62.7, 62.5, 62.2, 8.4.

STEP2: Cs_2CO_3 (0.76 g, 2.3 mmol) was added to the reaction solution, then it was degassed again. In a round bottomed flask, **2** (0.44 g, 1.2 mmol) was dried under vacuum, then under inert atmosphere was dissolved in degassed anhydrous THF (6 ml). This solution was added to the reaction mixture and was stirred at r.t. overnight. The reaction was checked through ^{31}P-NMR, when the signal of the mono substituted product disappeared (signal at 68.1 ppm), the reaction was stopped. The solution was filtered from the salt, the volume of the solution was reduced to 2-3 ml and was dropped in degassed pentane (30 ml) under hard stirring to precipitate the product, the precipitate was filtered and was dried under vacuum obtaining a yellow sticky soft solid as crude mixture (1.22 g, 0.08 mmol).

^{31}P{^{1}H}-NMR (400 MHz, $CDCl_3$) δ(ppm): 63.1, 62.8, 62.5, 8.4, -13.3.

STEP3: in a Schlenk Na_2SO_4 (0.60 g, 4.2 mmol), the crude mixture from the STEP2 (1.22 g, 0.08 mmol, moles were calculated doing an estimation of impurities through the NMR) and Pd(1,5-cyclooctadiene)Cl_2 (0.28 g, 1.0mmol) were dried under vacuum, then under inert atmosphere was added anhydrous CH_2Cl_2 degassed (40 ml) and was started the stirring. The reaction was checked through ^{31}P-NMR after 3h (disappearance of the signal at -13.. If the complexation was not complete was added a little amount of Pd(1,5-cyclooctadiene)Cl_2. The solution was filtered from the salt, then the volume of the solution was reduced to few ml and was dropped in pentane/Et_2O 4:1 (80 ml) under hard stirring to precipitate the product. The precipitate was filtered and was dried under vacuum. The product was obtained as a light orange solid (1.26 g, 0.1 mmol). **Yield = 83 %**

M.p.: >230°C. **Chemical Formula:** $C_{612}H_{468}Cl_{24}F_{156}N_{51}O_{42}P_{33}Pd_{12}S_{30}$. **Molecular Weight (theoretical):** 16284.32 g/mol. **^{1}H-NMR** (400 MHz, $CDCl_3$) δ(ppm): 8.18 (s, 12H, HC=N), 7.96 (s, 12H), 7.82 – 6.61 (m, 342H), 3.24 (m, 54H, CH_3-N), 3.05 (t, J = 8.2 Hz, 24H, CH_2-S), 2.47 – 2.23 (m, 24H-CH_2-CF_2). **^{13}C{^{1}H}-NMR** (101 MHz, $CDCl_3$) δ(ppm): 165.9, 151.3, 149.8, 148.9, 140.0, 137.7, 136.6, 134.1, 133.6, 132.6, 132.2, 131.6, 131.2, 129.1 (d, *J* = 9.9 Hz), 128.6 (d, *J* = 10.7 Hz), 125.3, 124.7, 122.5 (d, *J* = 5.0 Hz), 121.5, 121.2, 121.6 – 104.6 (complex pattern due to C-F coupling) 33.4 (d, *J* = 11.1 Hz, CH_2-S), 31.5 (t, *J* = 22.1 Hz, CH_3-N), 25.4 (CH_2-CF_2). **^{19}F-NMR** (377 MHz, $CDCl_3$) δ(ppm): -80.8, -114.0, -121.9, -122.9, -123.3, -126.2. **^{31}P{^{1}H}-NMR** (400 MHz, $CDCl_3$) δ(ppm): 62.6, 30.9, 8.3. **IR (neat) ν (cm^{-1}):** 1604, 1489, 1195, 1161, 1144, 918, 837, 789. **ICP-OS: Pd(%(p/p)) =** 7.8 ± 1.0%rsd; **Calculated Pd(%(p/p)) =** 7.8.

1.2.5 Synthesis of 3rdm-G2-[Pd]

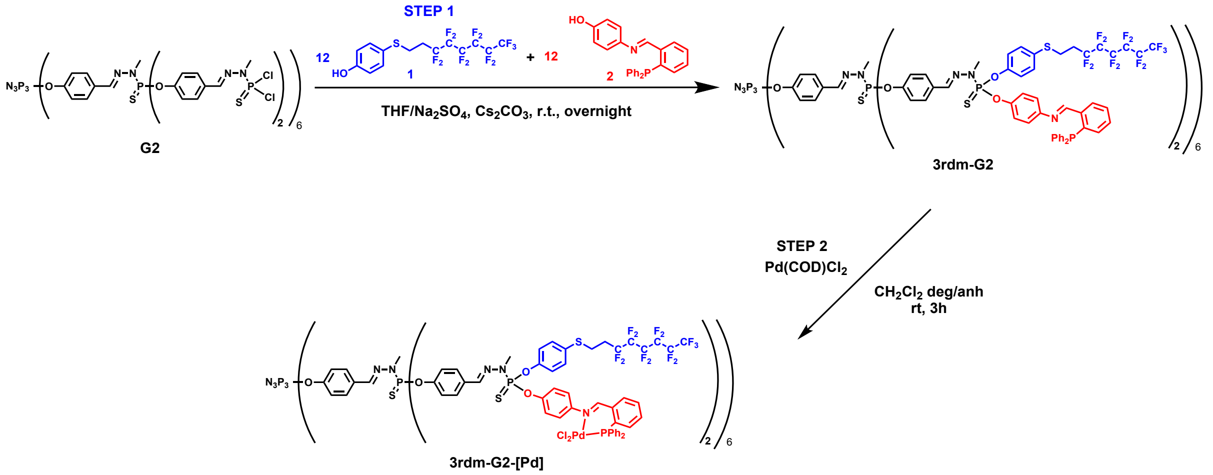

STEP1: in a Schlenk, equipped with an addition funnel, Cs_2CO_3 (0.97 g, 3.0 mmol) Na_2SO_4 (0.40 g, 2.8 mmol), **Gc$_2$** (0.30 g, 0.06 mmol), **1** (0.33 g, 0.7 mmol) and **2** (0.27 mg, 0.7 mmol) were dried under vacuum for 30 minutes. Then under inert atmosphere degassed anhydrous THF (15 ml) was added and was stirred, the reaction solution was left at r.t. overnight. The reaction was checked through ^{31}P-NMR, when the reaction was completed, was stopped and the solution was filtered from the salt, the volume of the solution was reduced to 2-3 ml and was dropped in degassed pentane (40 ml) under hard stirring to precipitate the product, the precipitate was filtered and was dried under vacuum obtaining a yellow sticky soft solid as crude mixture (0.86 g, 0.06 mmol).

^{31}P{^{1}H}-NMR (400 MHz, $CDCl_3$) δ(ppm): 63.0, 62.8, 62.5, 8.4, -13.3.

STEP2: in a Schlenk Na_2SO_4 (0.30 g, 2.1 mmol), the crude mixture from the STEP1 (0.86 g, 0.06 mmol, moles were calculated doing an estimation of impurities through the NMR) and Pd(1,5-cyclooctadiene)Cl_2 (0.19 g, 0.7 mmol) were dried under vacuum, then under inert atmosphere was added anhydrous CH_2Cl_2 degassed (20 ml) and was started the stirring. The reaction was checked through ^{31}P-NMR after 3h (disappearance of signal at -13.3 ppm). If the complexation was not complete was added a little amount of Pd(1,5-cyclooctadiene)Cl_2. The solution was filtered from the salt, the volume of the solution was reduced to 2-3 ml and was dropped in pentane/Et_2O 4:1 (70 ml) under hard stirring to precipitate the product. The precipitate was filtered and was dried under vacuum, obtaining the product as a light orange solid (0.82 g, 0.05 mmol). **Yield = 85%**

M.p.: >230°C. **Chemical Formula:** $C_{612}H_{468}Cl_{24}F_{156}N_{51}O_{42}P_{33}Pd_{12}S_{30}$. **Molecular Weight (theoretical):** 16284.32 g/mol. **^{1}H-NMR** (400 MHz, $CDCl_3$) δ(ppm): 8.16 (s, 12H, CH=N), 7.96 (s, 12H), 7.84 – 6.58 (complex absorption, 342H), 3.40 – 3.11 (m, 56H), 3.05 (t, 24H, CH_2-S), 2.46 – 2.27 (m, 24H, CH_2-CF_2). **^{13}C{^{1}H}-NMR** (101 MHz, $CDCl_3$) δ(ppm): 166.0, 151.3, 149.8, 148.9, 139.9, 137.7, 136.6, 134.1, 133.5, 132.6, 132.2, 131.7, 131.2, 129.2, 128.6, 125.4, 124.7, 122.4, 121.6, 120.4 – 106.9 (complex pattern due to C-F coupling), 33.4 (CH_2-S), 31.6 (t, J = 22.1 Hz), 25.4 (CH_2-CF_2). **^{19}F-NMR** (377 MHz, $CDCl_3$) δ(ppm): -80.9, -114.1, -122.0, -122.9, -123.3, -126.2. **^{31}P{^{1}H}-NMR** (162 MHz, $CDCl_3$) δ(ppm): 62.6, 30.9, 8.3. **IR (neat) ν (cm^{-1}):** 1603, 1489, 1436, 1191, 1159, 1141, 912, 834, 788. **ICP-OS: Pd(%(p/p))** = 7.1 ± 0.7%rsd; **Calculated Pd(%(p/p))** = 7.8.

1.2.6 Synthesis of 3prc-G3-[Pd]

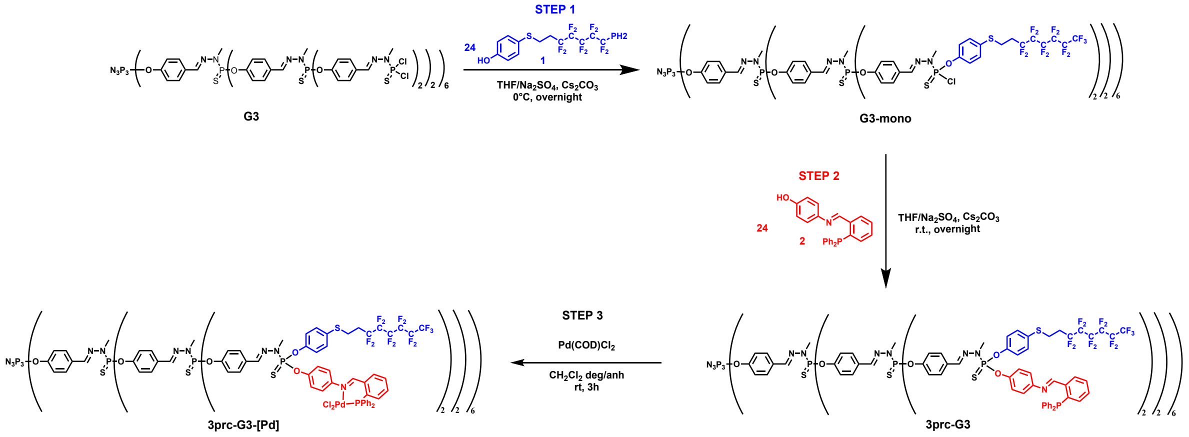

STEP1: in a Schlenk, equipped with an addition funnel, Cs_2CO_3 (0.33 g, 1.0 mmol), Na_2SO_4 (0.20 g, 1.4 mmol) and Gc_3 (0.30 g, 0.03 mmol) were dried under vacuum for 30 minutes. Then under inert atmosphere degassed anhydrous THF (7 ml) , was added and was stirred, the system was place in an ice bath. In a round bottomed flask, **1** (0.32 mg, 0.7 mmol) was dried under vacuum, then under inert atmosphere was dissolved in degassed anhydrous THF (5 ml). This solution was transferred in the addition funnel and was added to the reaction solution dropwise with a rate of a drop every 8/9 sec. The reaction solution was left under stirring overnight. The reaction was checked through ^{31}P-NMR, when the mono-substitution was complete, it was possible to proceed with the STEP2 in one pot reaction.

^{31}P{^{1}H}-NMR (400 MHz, $CDCl_3$) δ(ppm): 68.3, 62.4, 62.2, 8.2.

STEP2: Cs_2CO_3 (0.44 g, 1.3 mmol) was added to the reaction solution, then it was degassed again. In a round bottomed flask, **2** (2.52 mg, 0.7 mmol) was dried under vacuum, then under inert atmosphere was dissolved in degassed anhydrous THF (6 ml). This solution was added to the reaction solution and was left under stirring at r.t. overnight. The reaction was checked through ^{31}P-NMR, when the signal of the mono substituted product was disappeared (68.1 ppm), the reaction was stopped. The solution was filtered from the salt, the volume of the solution was reduced to 2-3 ml and was dropped in degassed pentane (30 ml) under hard stirring to precipitate the product, the precipitate was filtered and was dried under vacuum obtaining a yellow sticky soft solid as crude mixture (0.81 g, 0.03 mmol).

^{31}P{^{1}H}-NMR (400 MHz, $CDCl_3$) δ(ppm): 62.7, 62.5, 8.0, -13.3.

STEP3: in a Schlenk Na_2SO_4 (0.30 g, 2.1 mmol), the crude mixture from the STEP2 (0.81 g, 0.03 mmol, moles were calculated doing an estimation of impurities through the NMR) and Pd(1,5-

cyclooctadiene)Cl_2 (0.18 g, 0.6 mmol) were dried under vacuum, then under inert atmosphere degassed anhydrous CH_2Cl_2 (30 ml) was added and was stirred. The reaction was checked through ^{31}P-NMR after 3h (disappearing of the signal at -13.3). If the complexation was not complete was added a little amount of Pd(1,5-cyclooctadiene)Cl_2. The solution was filtered from the salt, then the volume of the solution was reduced to 2-3 ml and was dropped in pentane/Et_2O 4:1 (60 ml) under hard stirring to precipitate the product. The precipitate was filtered and was dried under vacuum. The product was obtained as a light orange solid (0.66 g, 0.02 mmol). **Yield = 72.0%**

.p.: >230°C. **Chemical Formula:** $C_{1272}H_{984}Cl_{48}F_{312}N_{111}O_{90}P_{69}Pd_{24}S_{66}$. **Molecular Weight (theoretical):** 33700.88 g/mol. **^{1}H-NMR** (400 MHz, $CDCl_3$) δ(ppm): 8.12 (s, 12H, CH=N), 7.89 (s, 12H), 7.77 – 6.57 (m, complex absorption 738H), 3.49 – 3.11 (m, 126H), 3.09 – 2.79 (m, 48H, CH_2-S), 2.47 – 2.10 (m, 48H, CH_2-CF_2). **^{13}C{^{1}H}-NMR** (400 MHz, $CDCl_3$) δ(ppm): 165.9, 151.3, 149.8, 148.9, 140.7 – 139.1 (m), 137.6, 137.0 – 136.0 (m), 135.5 – 133.1 (m), 133.1 – 130.6 (m), 129.8 – 127.8 (m), 125.0 – 124.1 (m), 123.0 – 122.2 (m), 122.2 – 120.9 (m), 121.6 – 104.6 (complex pattern due to C-F coupling) 33.3 (CH_2-S), 32.2 – 30.8 (m), 25.4 (CH_2-CF_2). **^{19}F-NMR** (400 MHz, $CDCl_3$) δ(ppm): -80.8, -114.1, -121.9, -122.9, -123.3, -126.2. **^{31}P{^{1}H}-NMR** (400 MHz, $CDCl_3$) δ(ppm): 62.5, 30.9, 28.6, 8.2. **IR (neat) ν (cm^{-1}):** 1608, 1492, 1194, 917, 838, 779. **ICP-OS: Pd(%(p/p)) =** 8.6 ± 2.3%rsd; **Calculated Pd(%(p/p)) =** 7.6.

1.2.7 Synthesis of 3rdm-G3-[Pd]

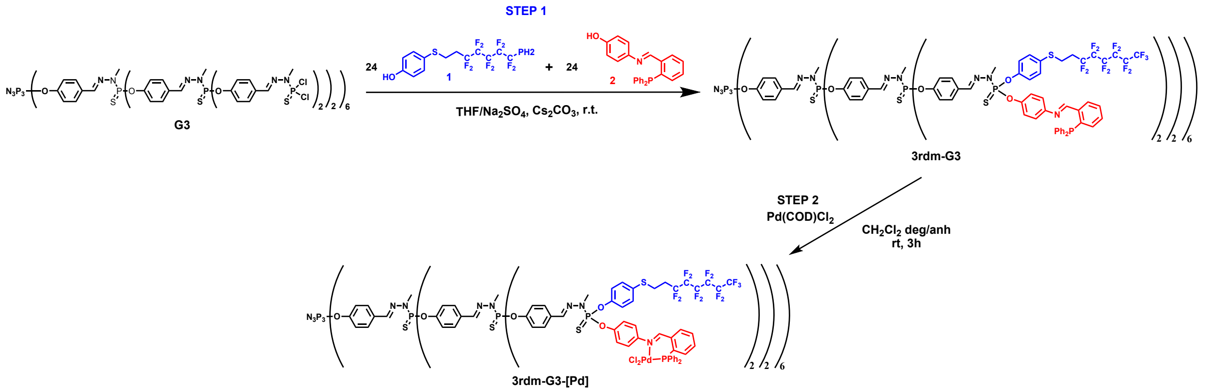

STEP1: in a Schlenk, equipped with an addition funnel, Cs_2CO_3 (0.91 g, 2.8 mmol) Na_2SO_4 (0.40 g, 2.8 mmol), **Gc$_3$** (0.30 g, 0.03 mmol), **1** (0.31 g, 0.7 mmol) and **2** (0.25 g, 0.7 mmol) were dried under vacuum for 30 minutes. Then under inert atmosphere degassed anhydrous THF (15 ml) was added and was stirred, the reaction solution was left at r.t. overnight. The reaction was checked through ^{31}P-NMR, when the reaction was completed disappearance signal starting material), was stopped and the solution was filtered from the salt. The volume of the solution was reduced to 2-3 ml and was dropped in degassed pentane (40 ml) under hard stirring to precipitate the product, the precipitate was filtered and was dried under vacuum obtaining a yellow sticky soft solid as crude mixture (0.88 g, 0.03 mmol).

^{31}P{^{1}H}-NMR (162 MHz, $CDCl_3$) δ(ppm): 62.9, 62.6, 62.4, 8.0, -13.3.

STEP2: in a Schlenk Na_2SO_4 (0.30 g, 2.1 mmol), the crude mixture from the **STEP1** (0.88 g, 0.03 mmol, moles were calculated doing an estimation of impurities through the NMR) and Pd(1,5-cyclootadiene)Cl_2 (0.18 g, 0.6 mmol) were dried under vacuum, then under inert atmosphere degassed anhydrous CH_2Cl_2 (20 ml) was added and was stirred. The reaction was checked through ^{31}P-NMR after 3h (disappearance of signal at -13.3 ppm). If the complexation was not complete

was added a little amount of Pd(1,5-cyclooctadiene)Cl_2. The solution was filtered from the salt, the volume of the solution was reduced to 2-3 ml and was dropped in pentane/Et_2O 4:1 (70 ml) under hard stirring to precipitate the product. The precipitate was filtered and was dried under vacuum, obtaining the product as a light orange solid (0.75 g, 0.03 mmol). **Yield = 93%**

M.p.: >230°C. **Chemical Formula:** $C_{1272}H_{984}Cl_{48}F_{312}N_{111}O_{90}P_{69}Pd_{24}S_{66}$. **Molecular Weight (theoretical):** 33700.88 g/mol. **^{1}H-NMR** (400 MHz, $CDCl_3$) δ(ppm): 8.17 (s, 24H, CH=N), 7.95 (s, 24H), 7.83 – 6.48 (complex absorption, 714H), 3.57 – 3.12 (m, 126H), 3.04 (t, *J* = 8.3 Hz, 48H, CH_2-S), 2.46 – 2.19 (m, 48H, CH_2-CF_2). **^{13}C{^{1}H}-NMR** (101 MHz, $CDCl_3$) δ(ppm): 166.1, 151.3, 149.8, 148.9, 139.9, 137.8, 136.6, 134.1, 132.6, 132.2, 131.7, 131.2, 129.2, 128.7, 125.3, 124.7, 122.4, 121.6, 120.9 – 106.5 (complex pattern due to C-F coupling), 33.3 (CH_2-S), 31.6 (t, *J* = 21.7 Hz), 25.4 (CH_2-CF_2). **^{19}F-NMR** (377 MHz, $CDCl_3$) δ(ppm): -80.8, -114.1, -122.0, -122.9, -123.3, -126.2. **^{31}P{^{1}H}-NMR** (162 MHz, $CDCl_3$) δ(ppm): 62.6, 31.7, 31.0, 8.3. **IR (neat) ν (cm^{-1}):** 1603, 1489, 1436, 1191, 1160, 1142, 913, 835. **ICP-OS: Pd(%(p/p)) =** 7.1 ± 2.9%rsd; **Calculated Pd(%(p/p)) =** 7.6.

1.3 General procedure for catalysis

1.3.1 Stille cross-coupling

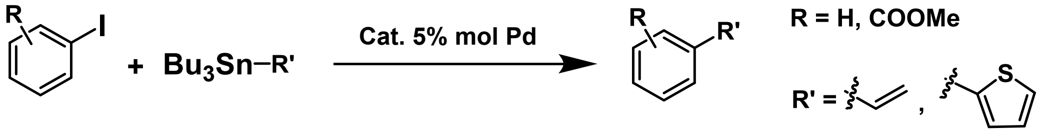

In a Schlenk the catalyst (5% mol Pd) was dissolved under inert atmosphere in THF-d8 (1.25 ml) and stirred. The aryl halide (0.26 mmol) and tributyltin compound (0.26 mmol) were added to the reaction mixture, and it was warmed to 50°C. Sampling procedure: a drop of the reaction solution was taken after 1, 2, 3, 5, 7 hours through a Pasteur and dissolved in 0.5 ml of $CDCl_3$, it was analysed through ^{1}H-NMR. After 22h the reaction mixture was cooled down at r.t., under stirring was added pentane/Et_2O 4:1 (20 ml) to precipitate the catalyst. The solid was filtered and dried under vacuum.

1.3.2 Heck cross-coupling

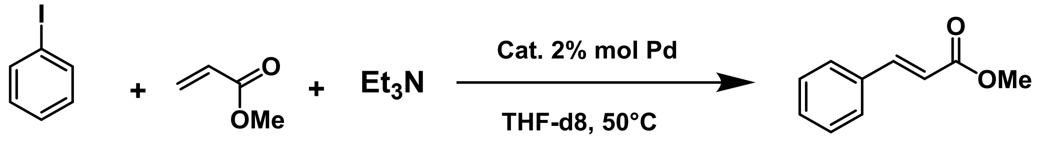

In a Schlenk the catalyst (2% mol Pd) was dissolved under inert atmosphere in THF-d8 (2 ml). Under stirring Iodobenzene (0.08 ml, 0.67 mmol), methyl acrylate (0.12 ml, 1.34 mmol) and triethylamine (0.19 ml, 1.34 mmol) were added in the reaction mixture. The solution was warmed to 50°C. The conversion was followed through ^{1}H-NMR. Sampling procedure: a drop of the reaction solution was taken after 1.5, 3, 4.5, 6, 24, 30 hours through a Pasteur and dissolved in 0.5 ml of $CDCl_3$, it was analysed through ^{1}H-NMR. After 30 h the reaction mixture was cooled down at r.t., under stirring was added pentane/Et_2O 4:1 (20 ml) to precipitate the catalyst. The solid was filtered and dried under vacuum.

1.4 NMR Spectra

M-mono

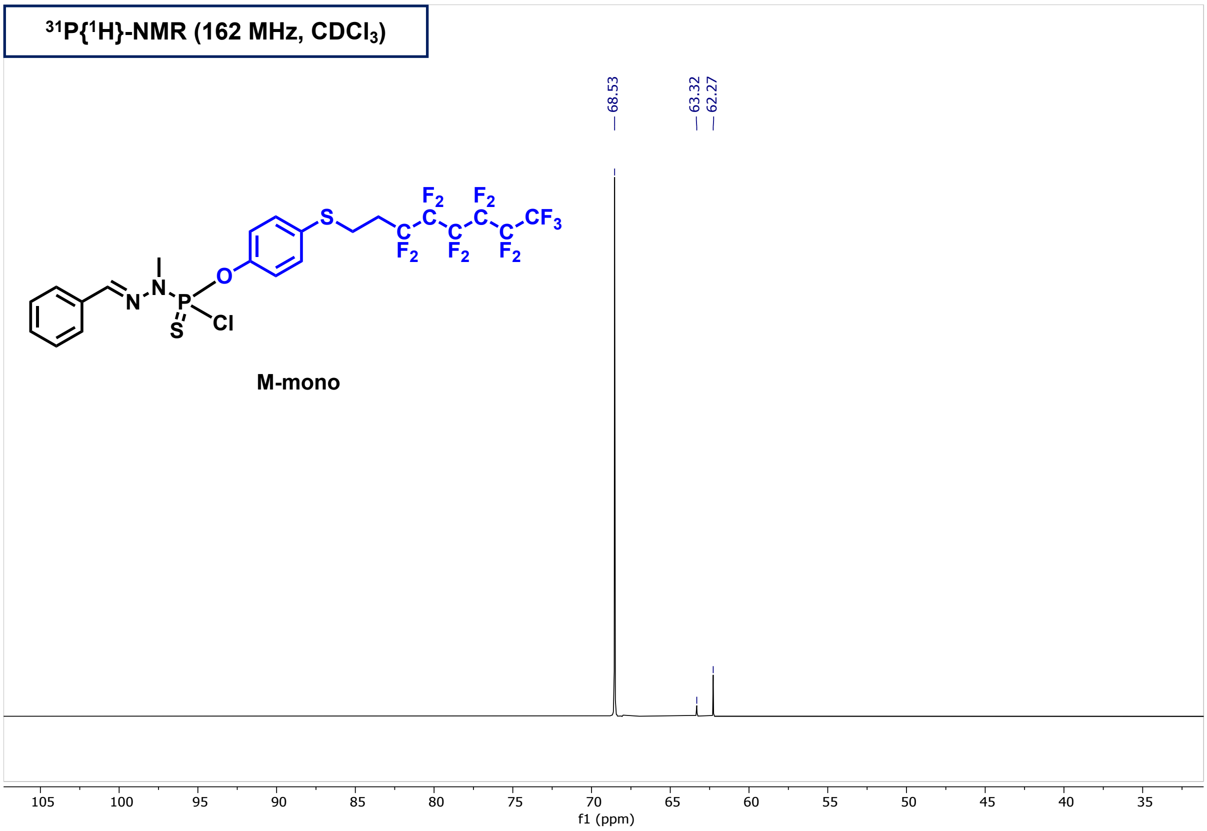

3prc

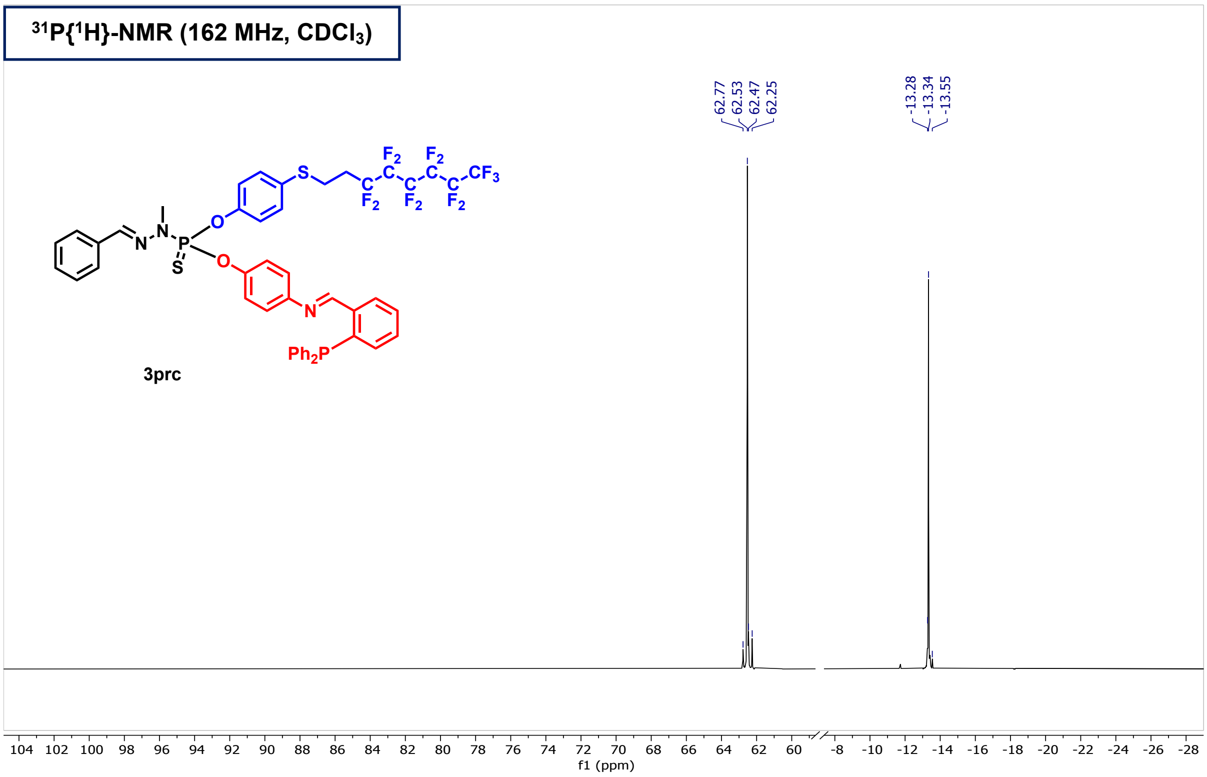

3prc-[Pd]

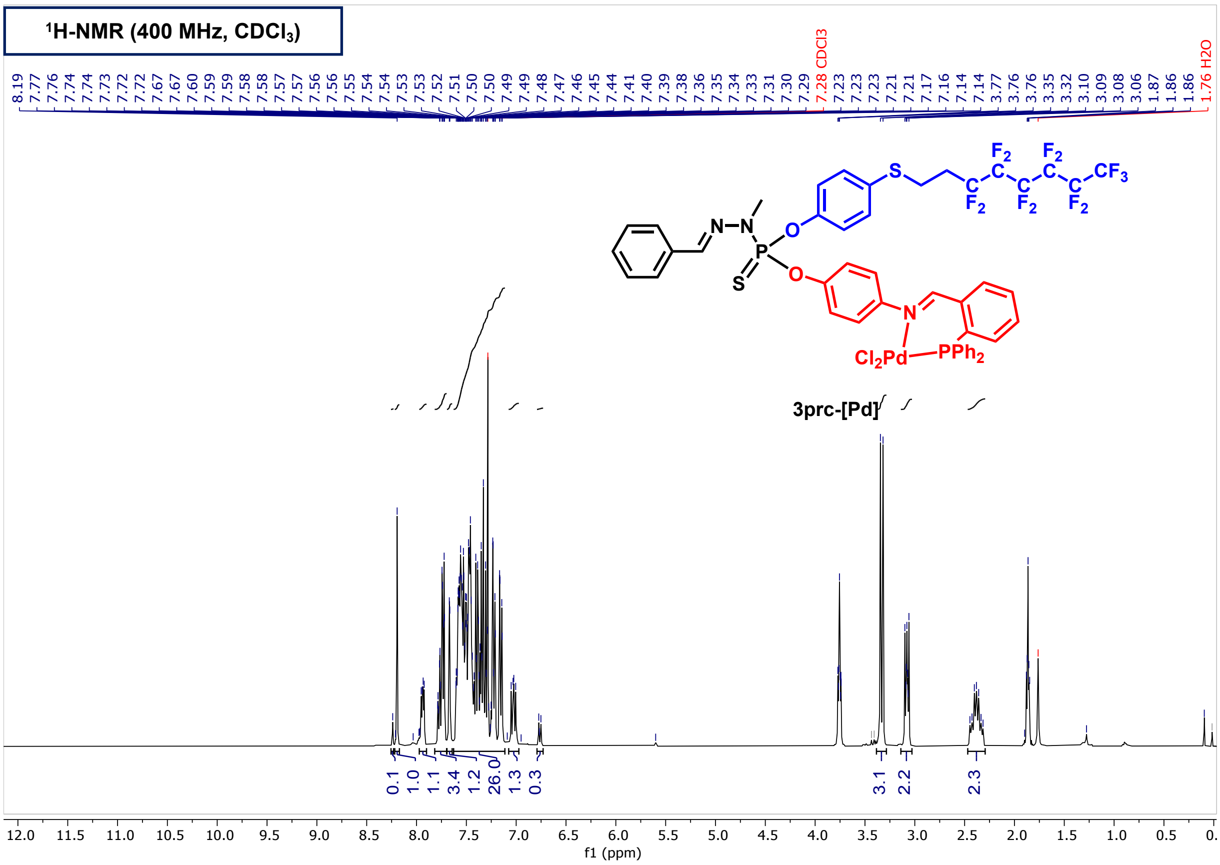

1H-NMR (400 MHz, CDCl3)
8.19 7.77 7.76 7.74 7.74 7.73 7.72 7.72 7.67 7.67 7.60 7.59 7.59 7.58 7.58 7.57 7.57 7.56 7.56 7.55 7.54 7.54 7.53 7.53 7.52 7.51 7.50 7.50 7.49 7.49 7.48 7.47 7.46 7.45 7.44 7.41 7.40 7.39 7.38 7.36 7.35 7.34 7.33 7.31 7.30 7.29 7.28 CDCl3 7.23 7.23 7.23 7.21 7.21 7.17 7.16 7.14 7.14 3.77 3.76 3.76 3.35 3.32 3.10 3.09 3.08 3.06 1.87 1.86 1.86 1.76 H2O
3prc-[Pd]
0.1 1.0 1.1 3.4 1.2 26.0 1.3 0.3 3.1 2.2 2.3
f1 (ppm)

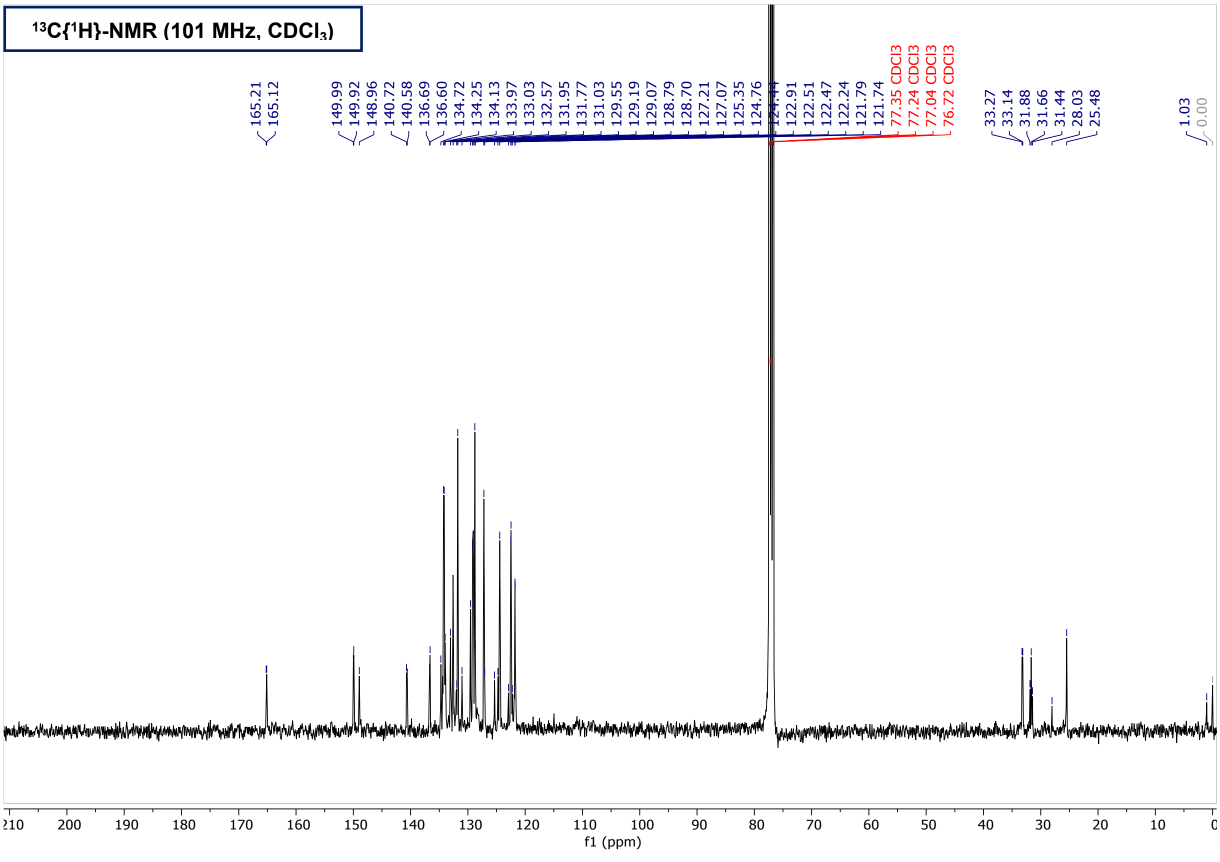

13C{1H}-NMR (101 MHz, CDCl3)
165.21 165.12 149.99 149.92 148.96 140.72 140.58 136.69 136.60 134.72 134.25 134.13 133.97 133.03 132.57 131.95 131.77 131.03 129.55 129.19 129.07 128.79 128.70 127.21 127.07 125.35 124.76 122.91 122.51 122.47 122.24 121.79 121.74 77.35 CDCl3 77.24 CDCl3 77.04 CDCl3 76.72 CDCl3 33.27 33.14 31.88 31.66 31.44 28.03 25.48 1.03 0.00
f1 (ppm)

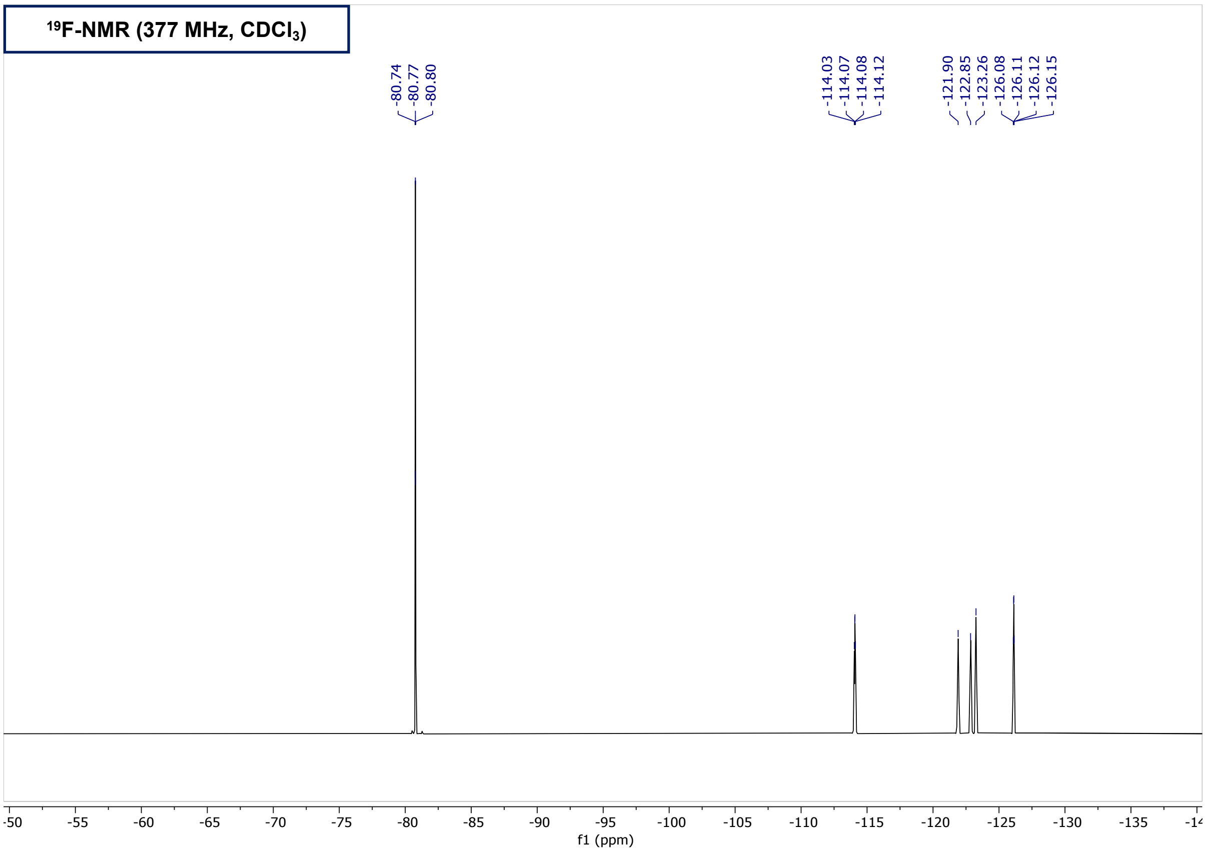
19F-NMR (377 MHz, CDCl3)
-80.74
-80.77
-80.80
-114.03
-114.07
-114.08
-114.12
-121.90
-122.85
-123.26
-126.08
-126.11
-126.12
-126.15
f1 (ppm)

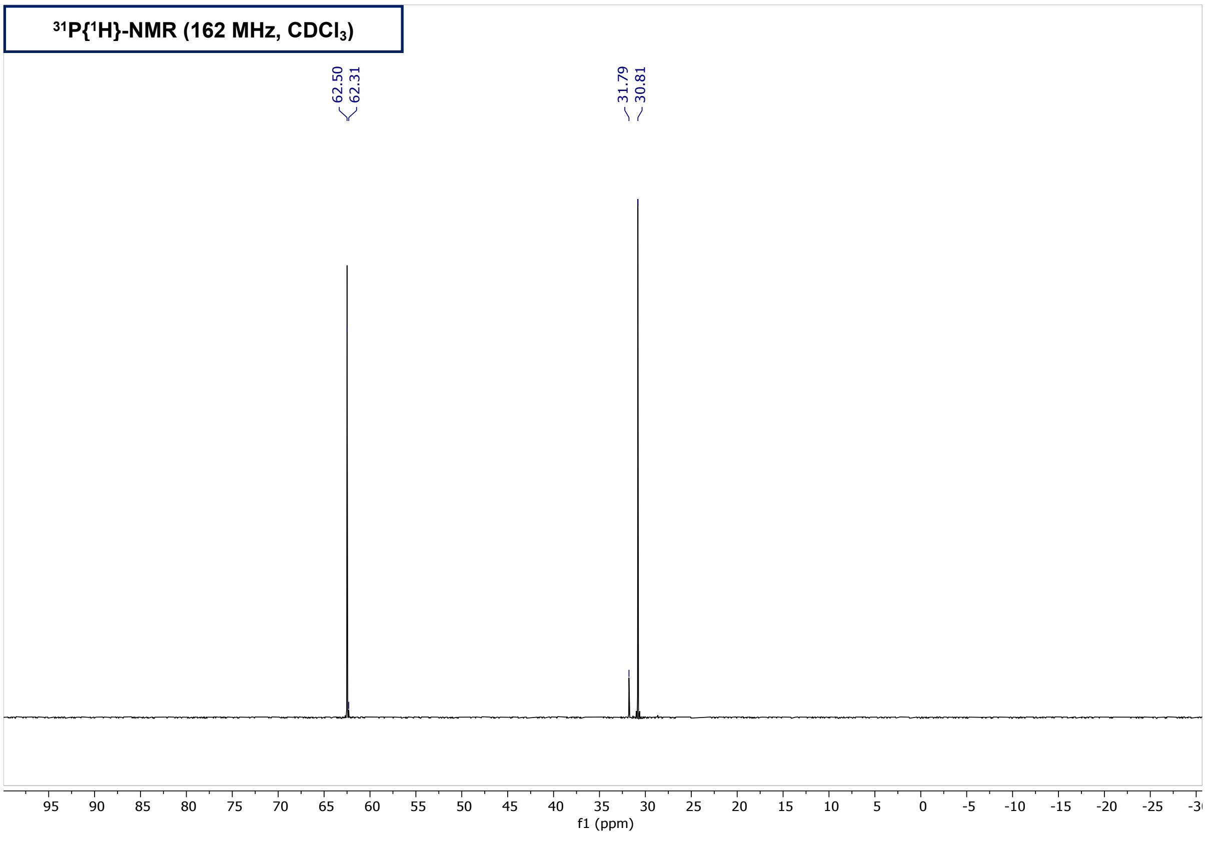
31P{1H}-NMR (162 MHz, CDCl3)
62.50
62.31
31.79
30.81
f1 (ppm)

G1-mono

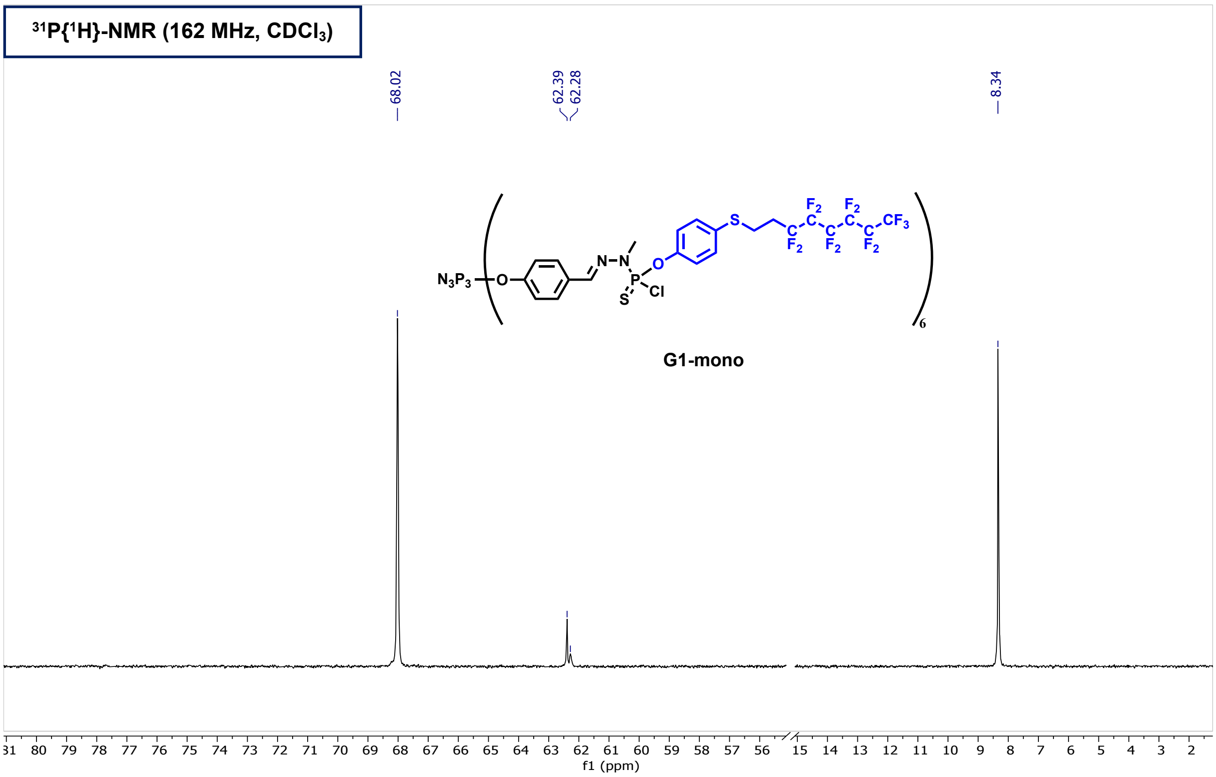

3prc-G1

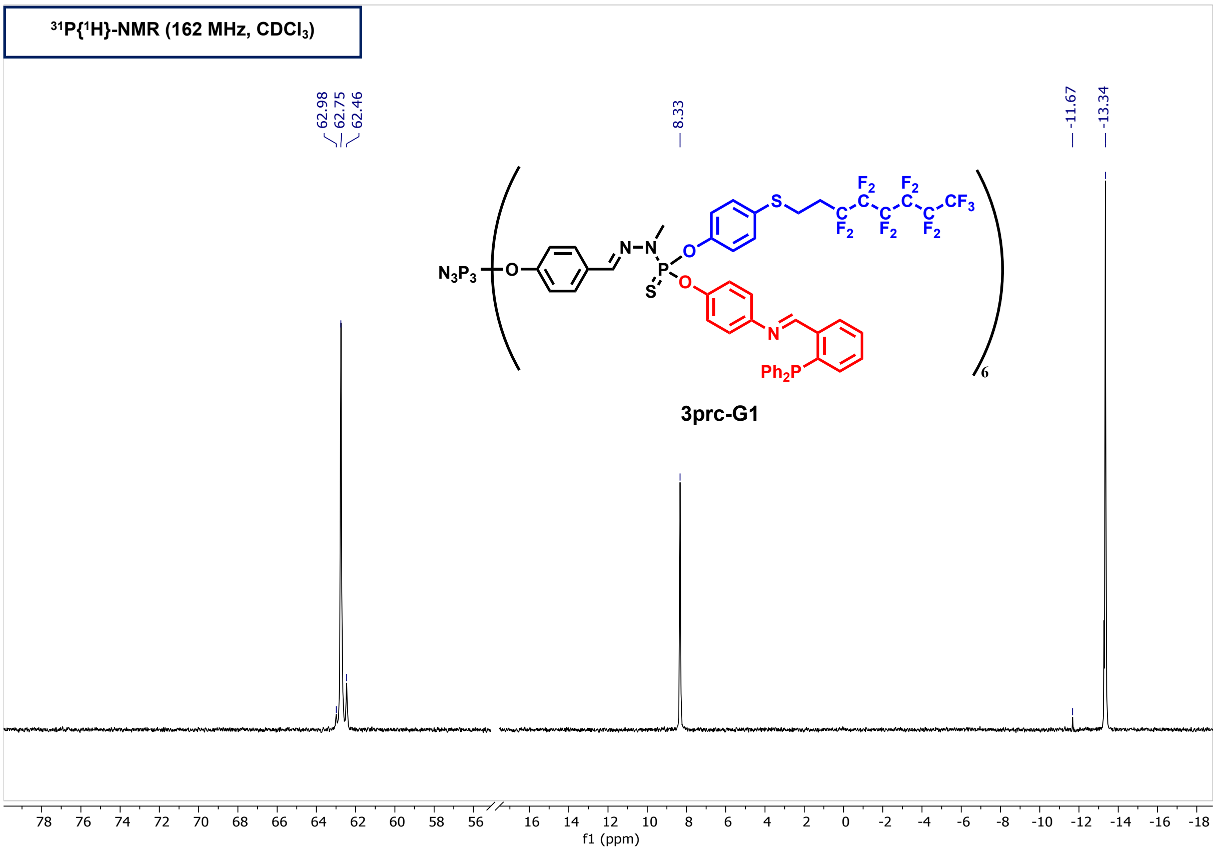

3prc-G1-[Pd]

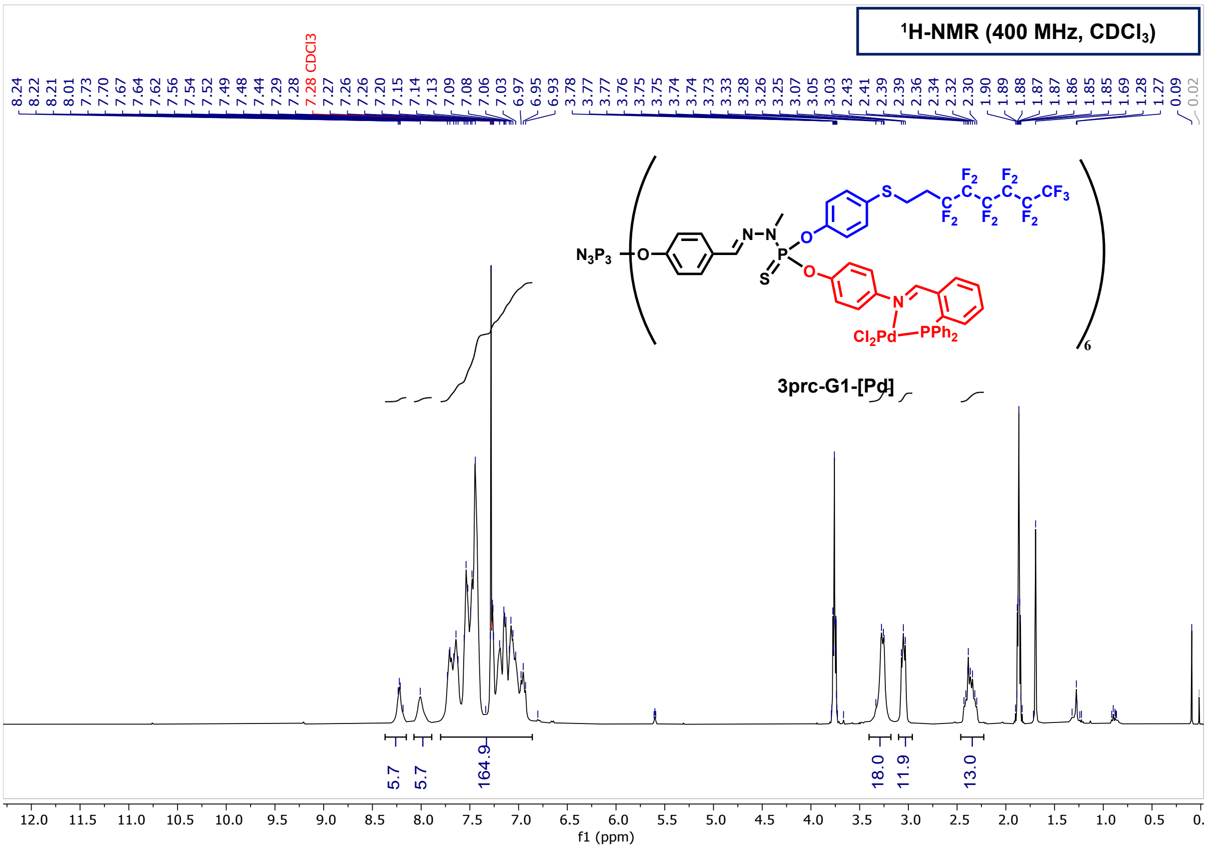

1H-NMR (400 MHz, CDCl3)
3prc-G1-[Pd]
f1 (ppm)

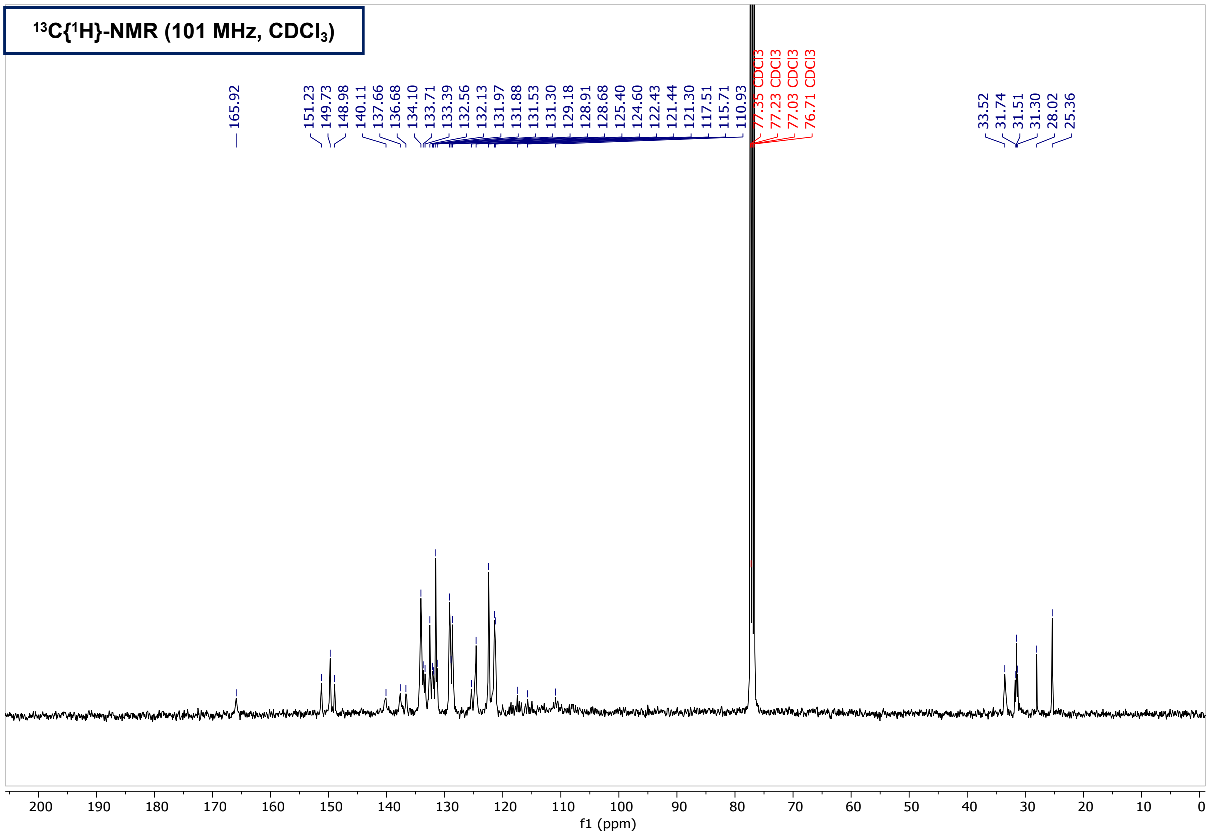

13C{1H}-NMR (101 MHz, CDCl3)
f1 (ppm)

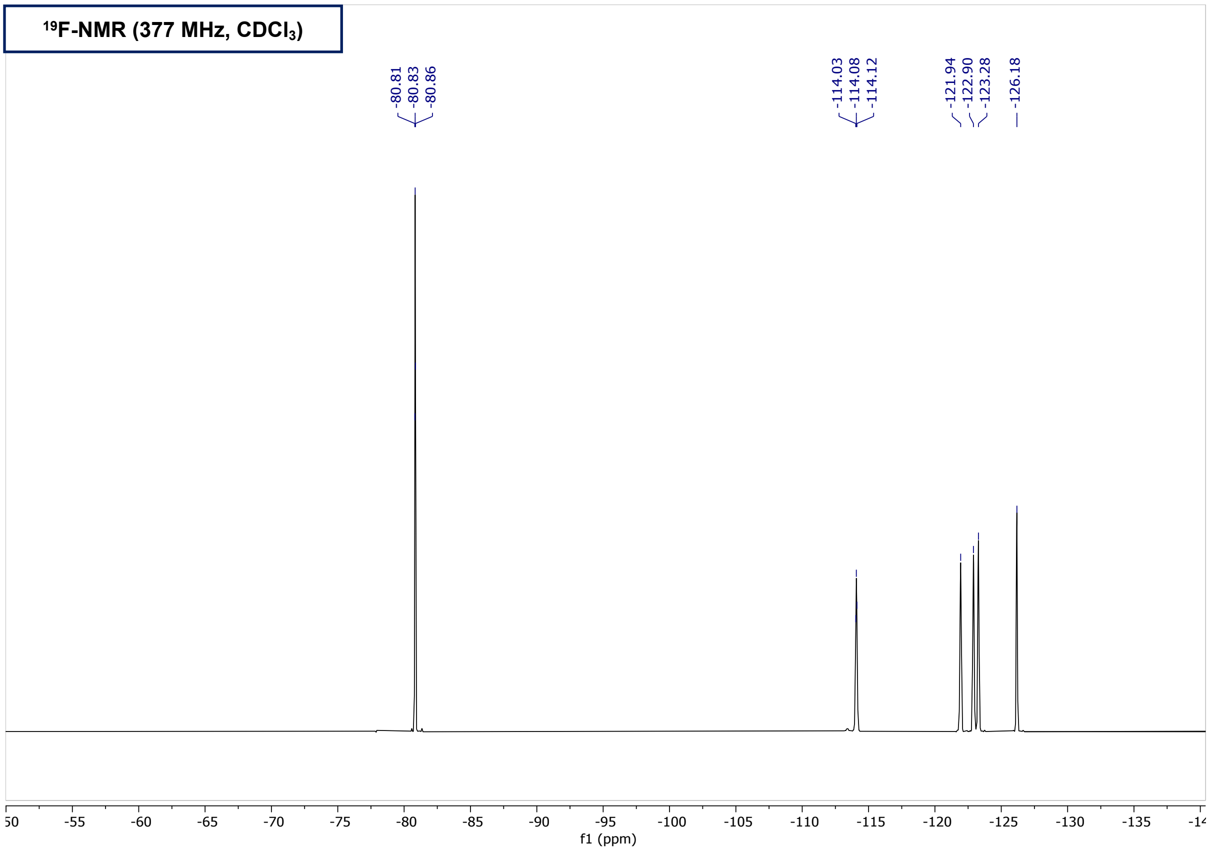

19F-NMR (377 MHz, CDCl3)
-80.81
-80.83
-80.86
-114.03
-114.08
-114.12
-121.94
-122.90
-123.28
-126.18
f1 (ppm)

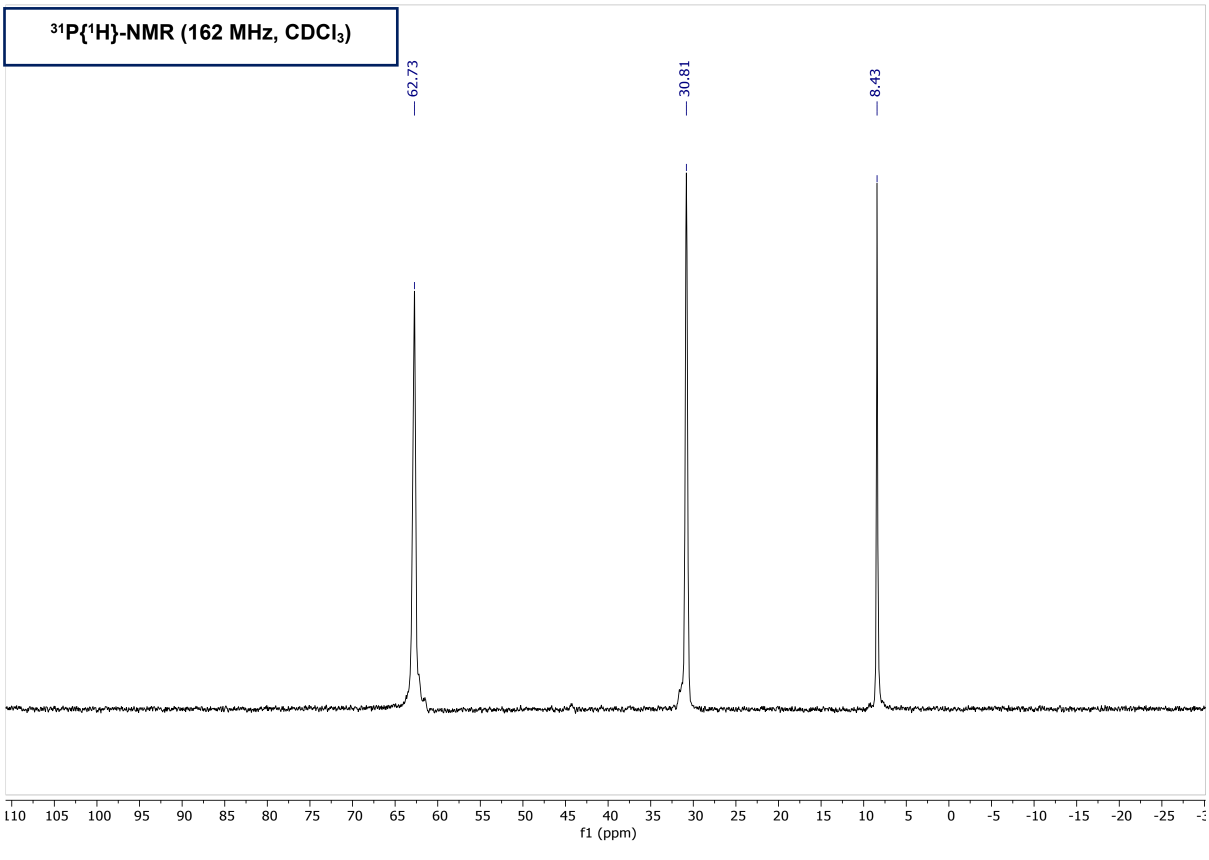

31P{1H}-NMR (162 MHz, CDCl3)
62.73
30.81
8.43
f1 (ppm)

3rdm-G1

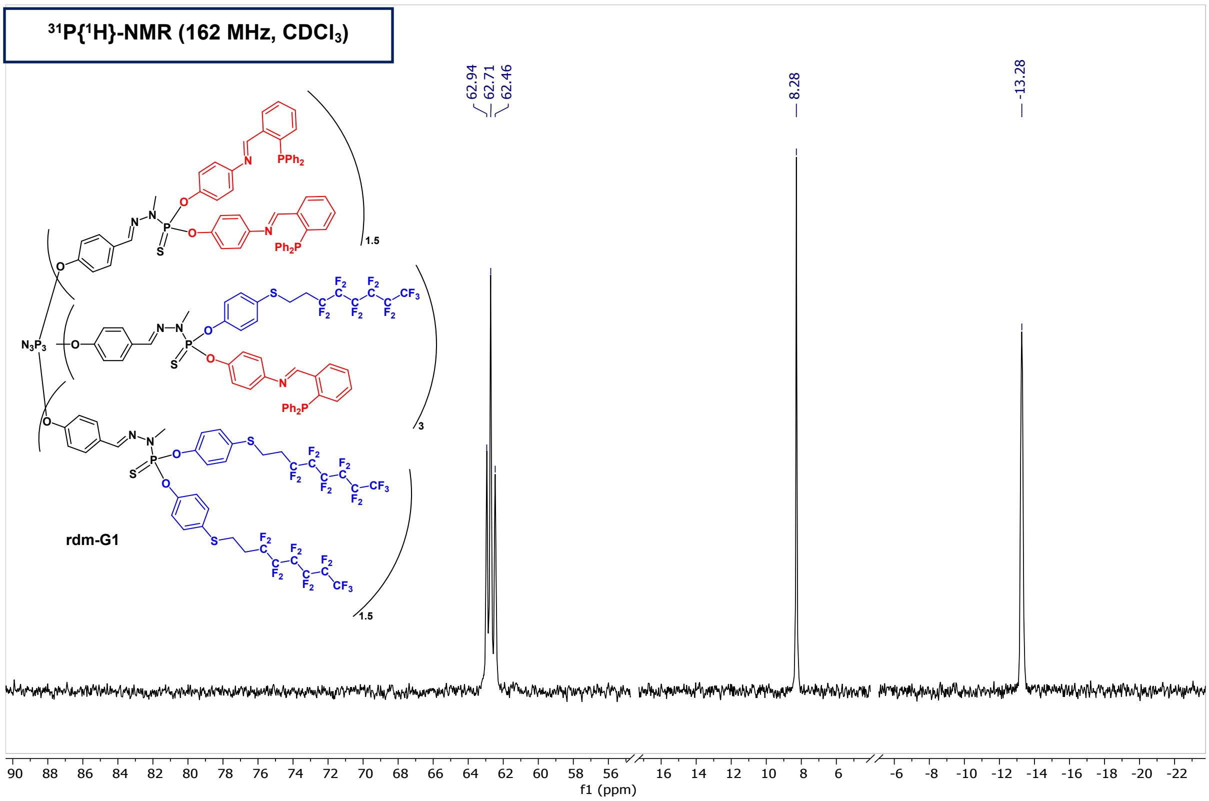

3rdm-G1-[Pd]

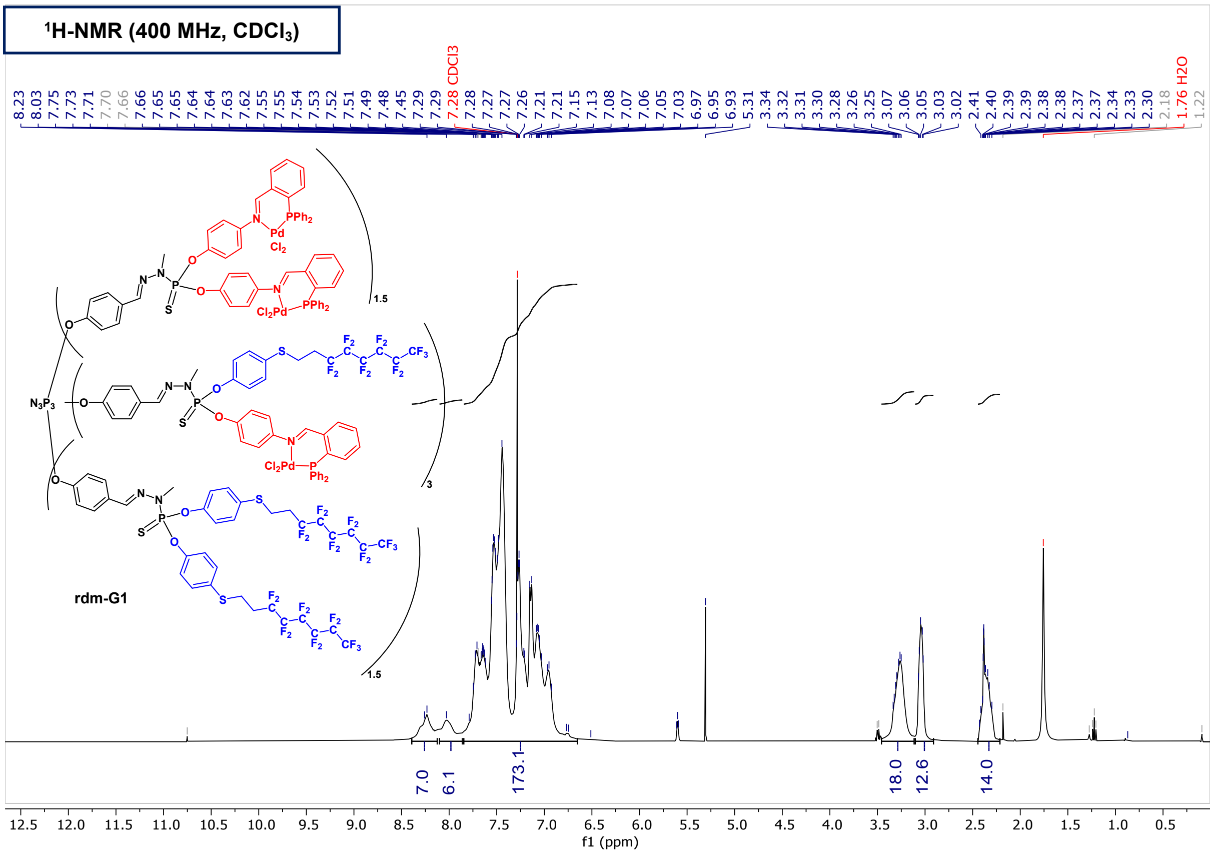

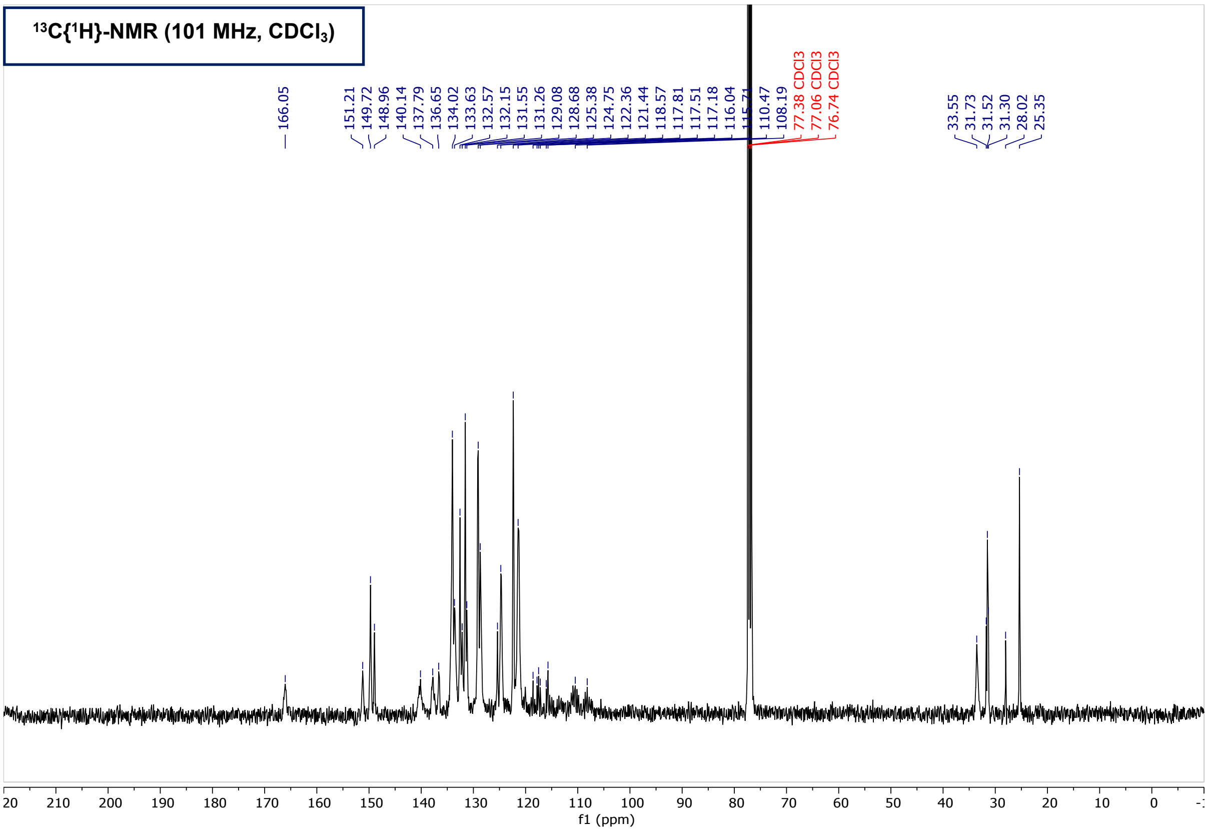

$^{13}C\{^1H\}$-NMR (101 MHz, CDCl3)
166.05
151.21
149.72
148.96
140.14
137.79
136.65
134.02
133.63
132.57
132.15
131.55
131.26
129.08
128.68
125.38
124.75
122.36
121.44
118.57
117.81
117.51
117.18
116.04
110.47
108.19
77.38 CDCl3
77.06 CDCl3
76.74 CDCl3
33.55
31.73
31.52
31.30
28.02
25.35
f1 (ppm)

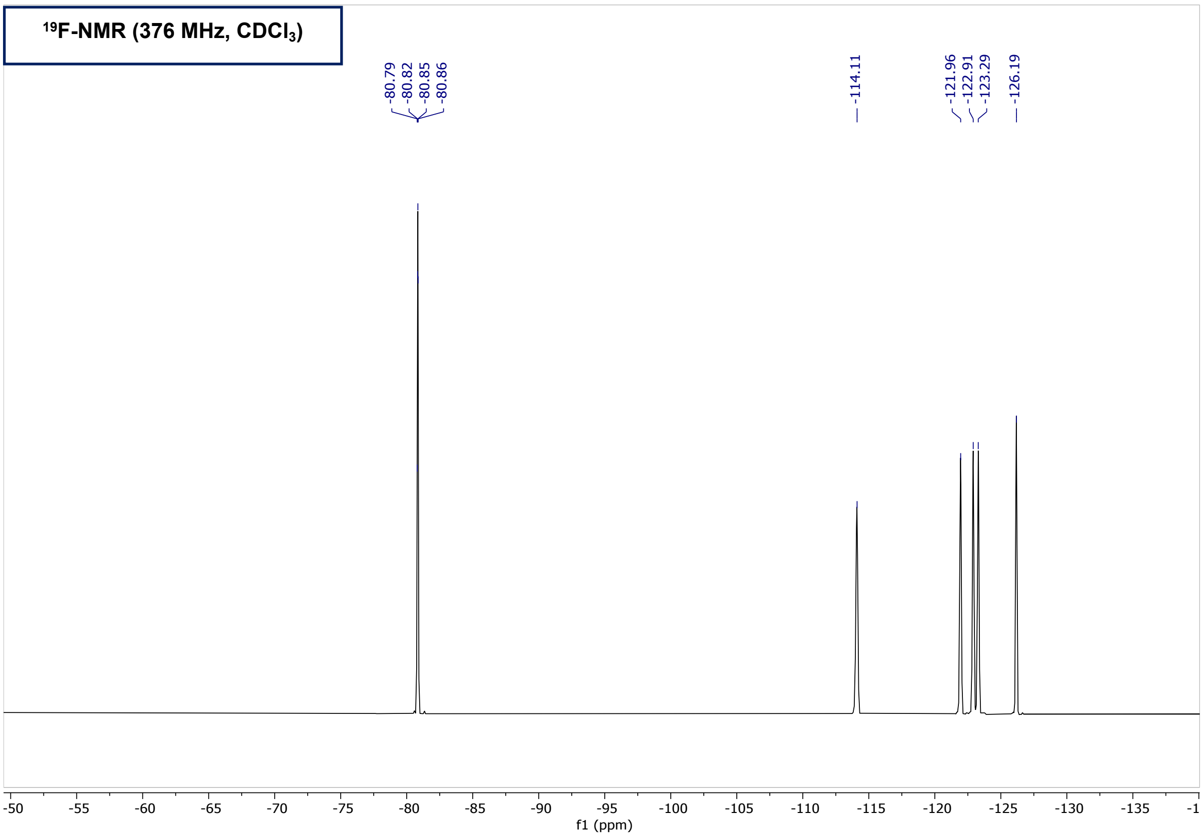

^{19}F-NMR (376 MHz, CDCl3)
-80.79
-80.82
-80.85
-80.86
-114.11
-121.96
-122.91
-123.29
-126.19
f1 (ppm)

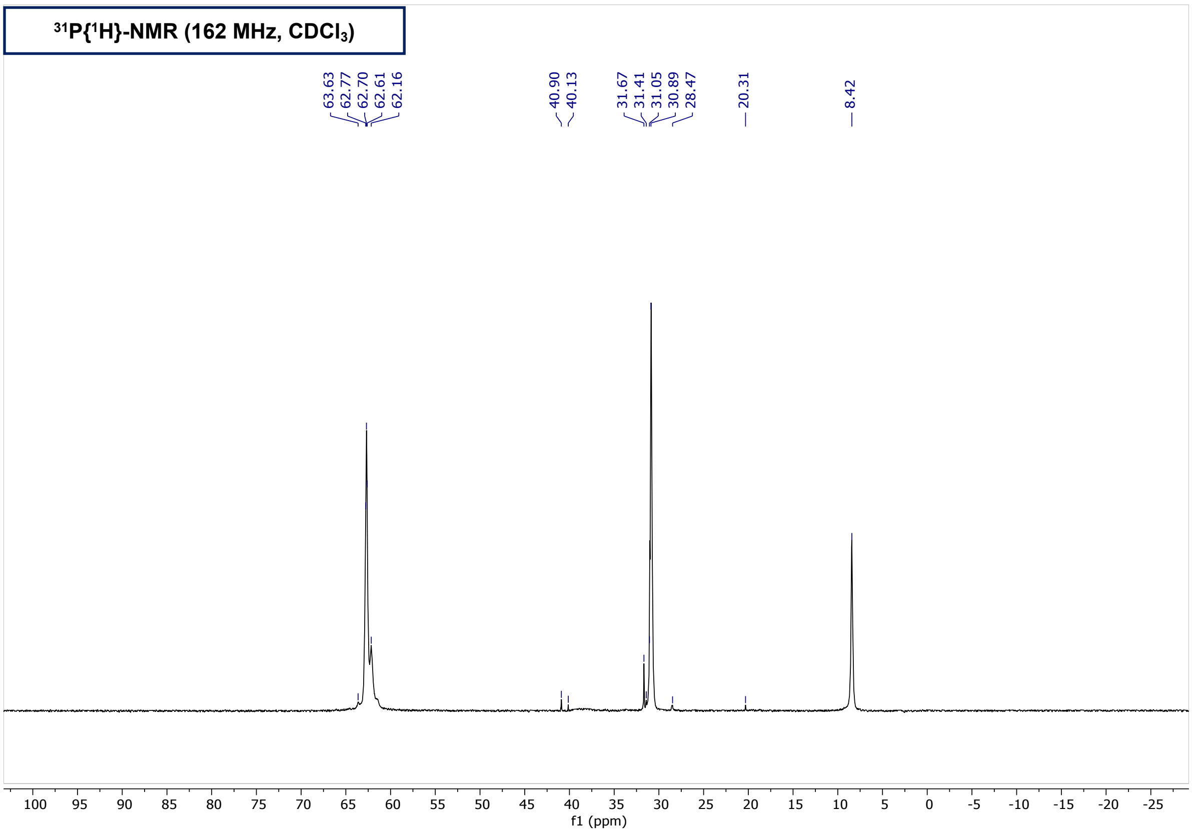

31P{1H}-NMR (162 MHz, CDCl3)
63.63
62.77
62.70
62.61
62.16
40.90
40.13
31.67
31.41
31.05
30.89
28.47
20.31
8.42
f1 (ppm)

G2-mono

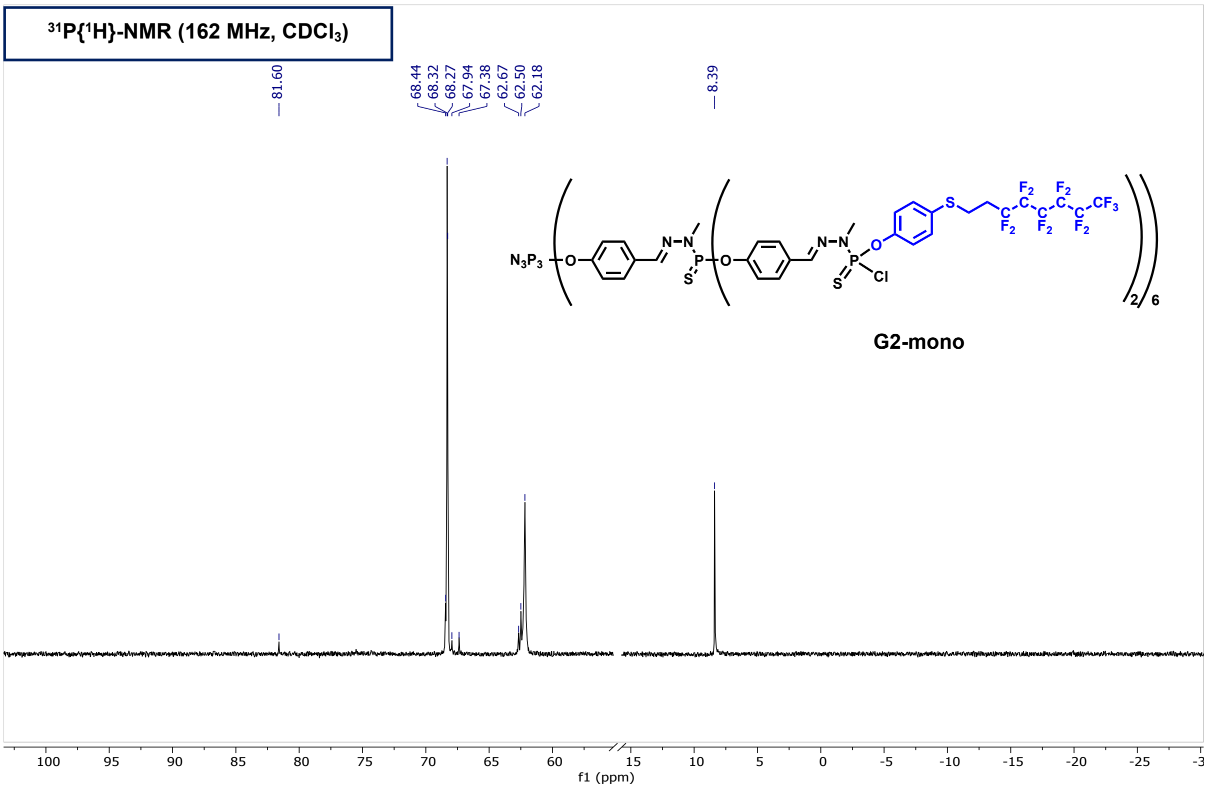

31P{1H}-NMR (162 MHz, CDCl3)
81.60
68.44
68.32
68.27
67.94
67.38
62.67
62.50
62.18
8.39
N3P3
G2-mono
f1 (ppm)

3prc-G2

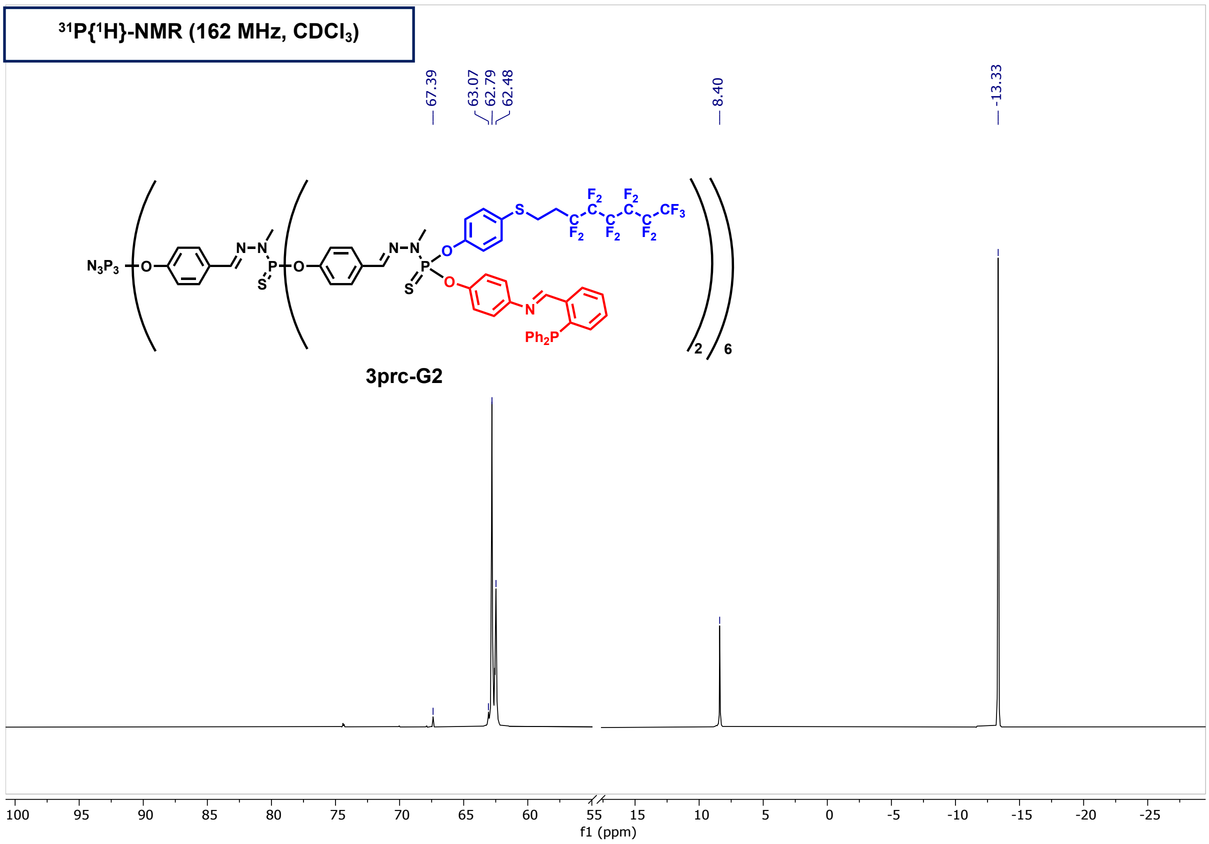

3prc-G2-[Pd]

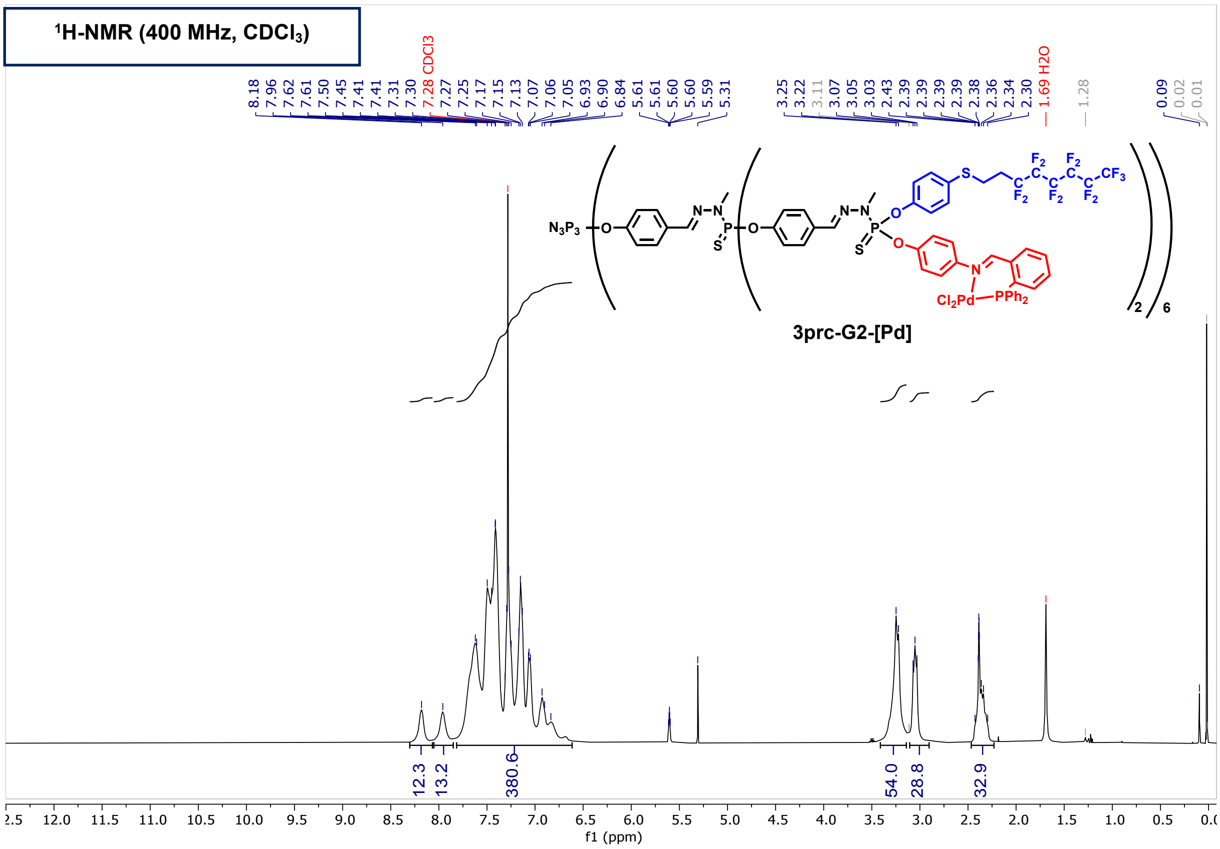

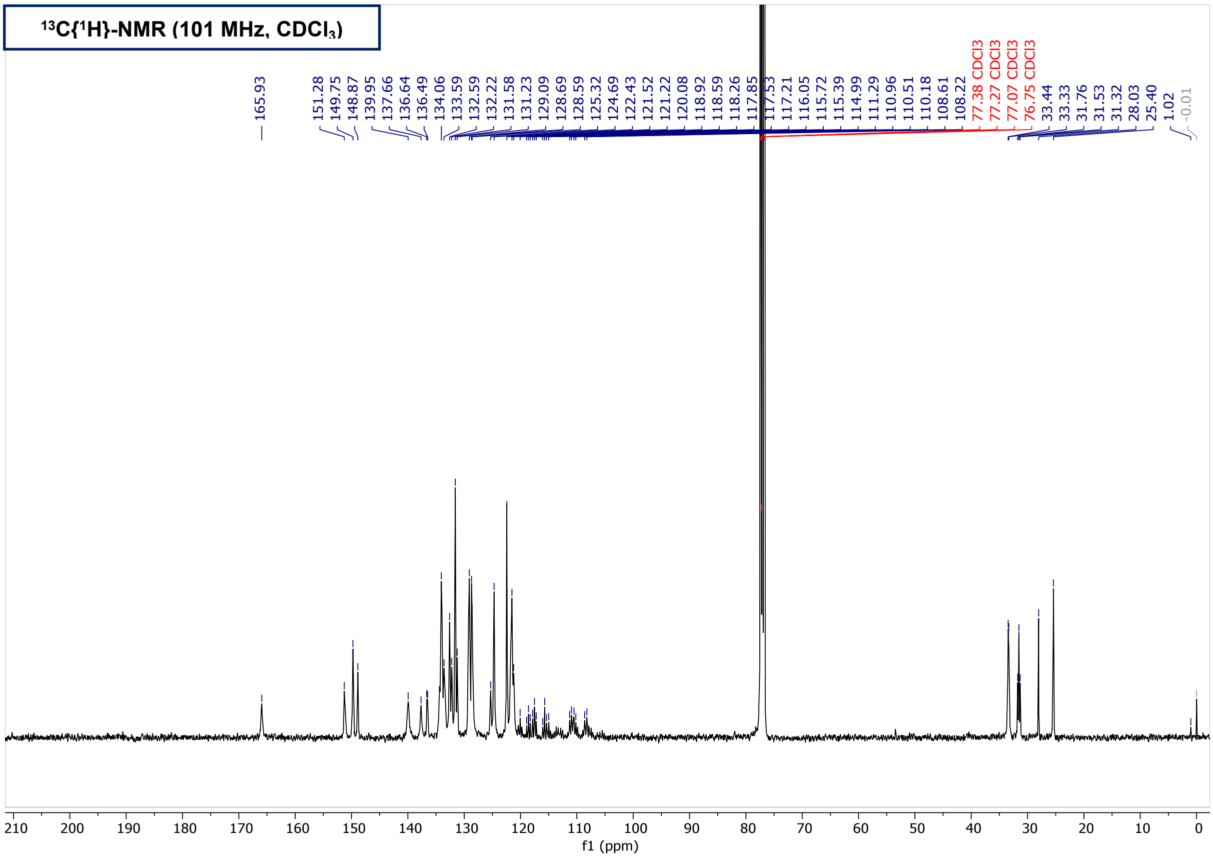

$^{13}C\{^1H\}$-NMR (101 MHz, CDCl$_3$)
165.93
151.28
149.75
148.87
139.95
137.66
136.64
136.49
134.06
133.59
132.59
132.22
131.58
131.23
129.09
128.69
128.59
125.32
124.69
122.43
121.52
121.22
120.08
118.92
118.59
118.26
117.85
117.53
117.21
116.05
115.72
115.39
114.99
111.29
110.96
110.51
110.18
108.61
108.22
77.38 CDCl3
77.27 CDCl3
77.07 CDCl3
76.75 CDCl3
33.44
33.33
31.76
31.53
31.32
28.03
25.40
1.02
-0.01
f1 (ppm)

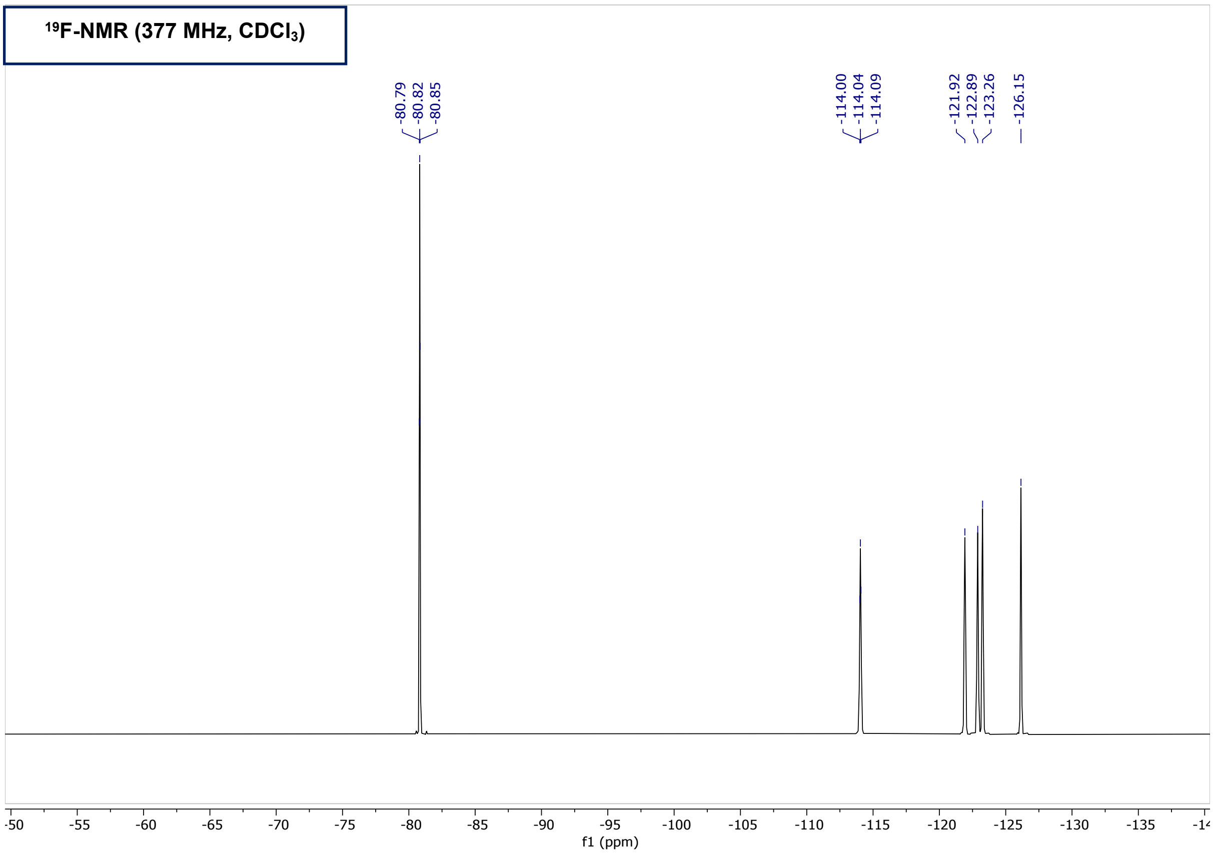

^{19}F-NMR (377 MHz, CDCl$_3$)
-80.79
-80.82
-80.85
-114.00
-114.04
-114.09
-121.92
-122.89
-123.26
-126.15
f1 (ppm)

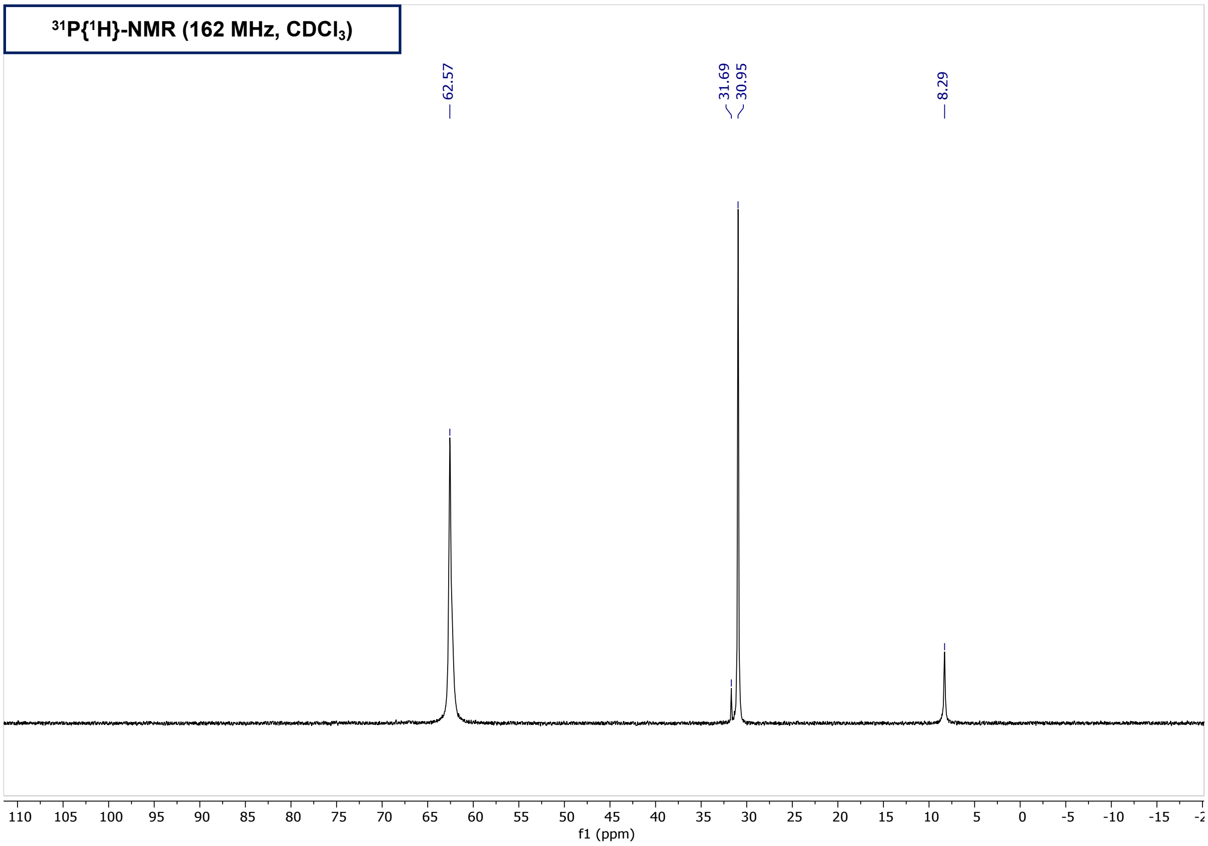

$^{31}P\{^1H\}$-NMR (162 MHz, $CDCl_3$)
62.57
31.69
30.95
8.29
f1 (ppm)

3rdm-G2

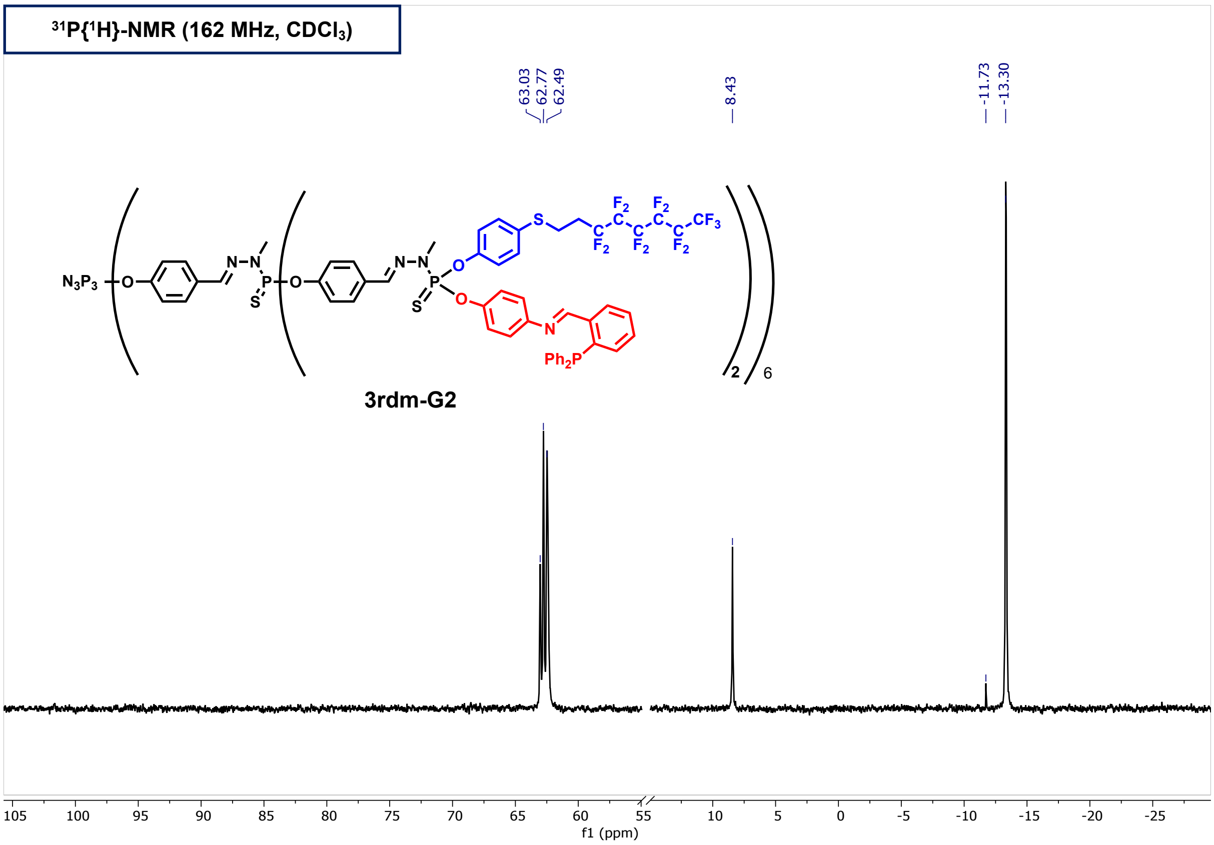

$^{31}P\{^1H\}$-NMR (162 MHz, $CDCl_3$)
63.03
62.77
62.49
8.43
-11.73
-13.30
3rdm-G2
f1 (ppm)

3rdm-G2-[Pd]

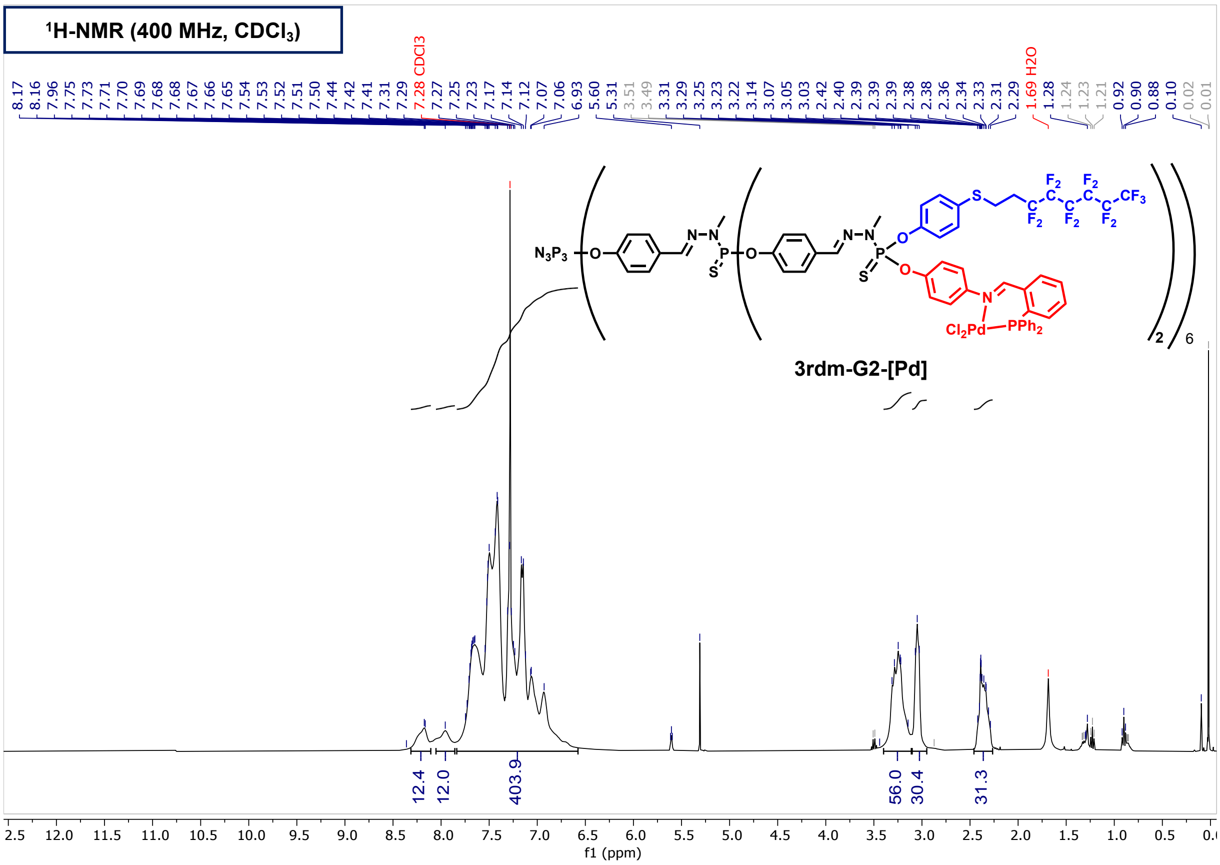
^{1}H-NMR (400 MHz, $CDCl_3$)
3rdm-G2-[Pd]
f1 (ppm)

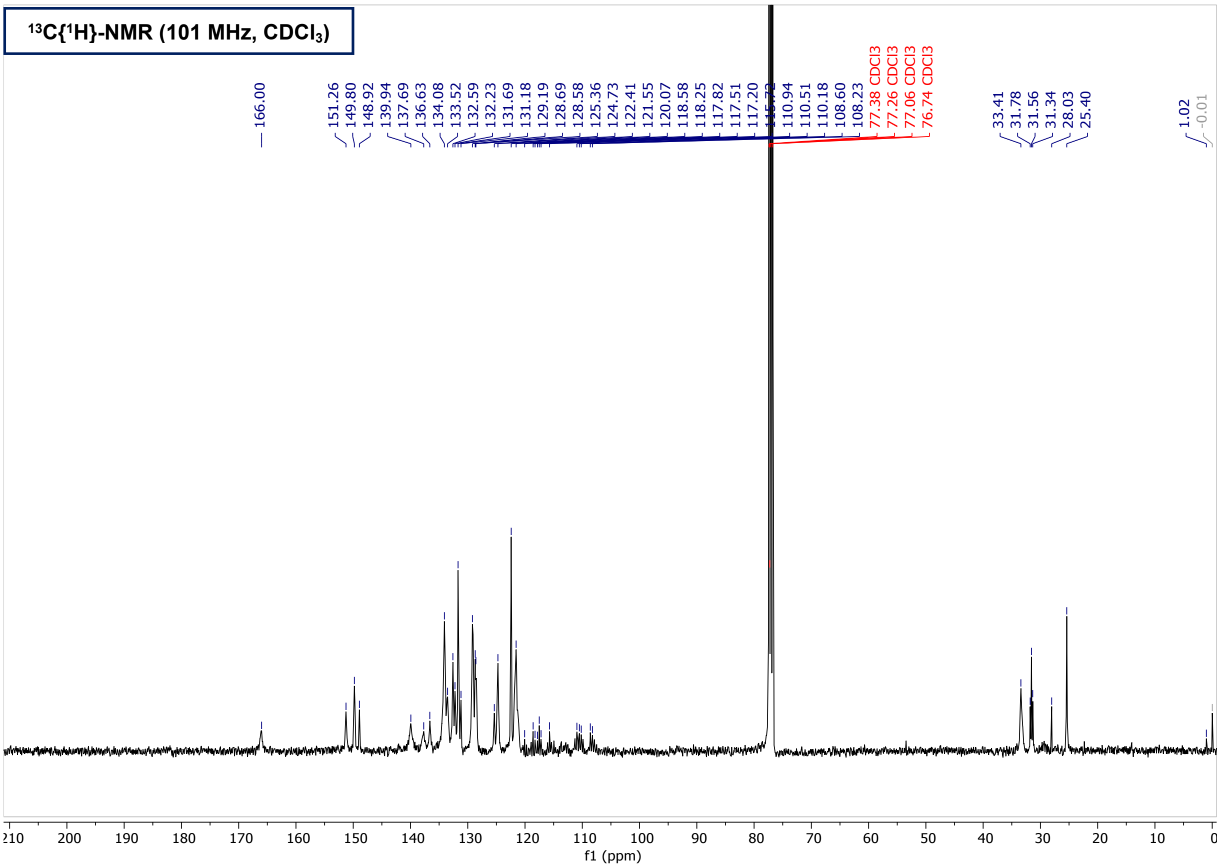
$^{13}C\{^1H\}$-NMR (101 MHz, $CDCl_3$)
f1 (ppm)

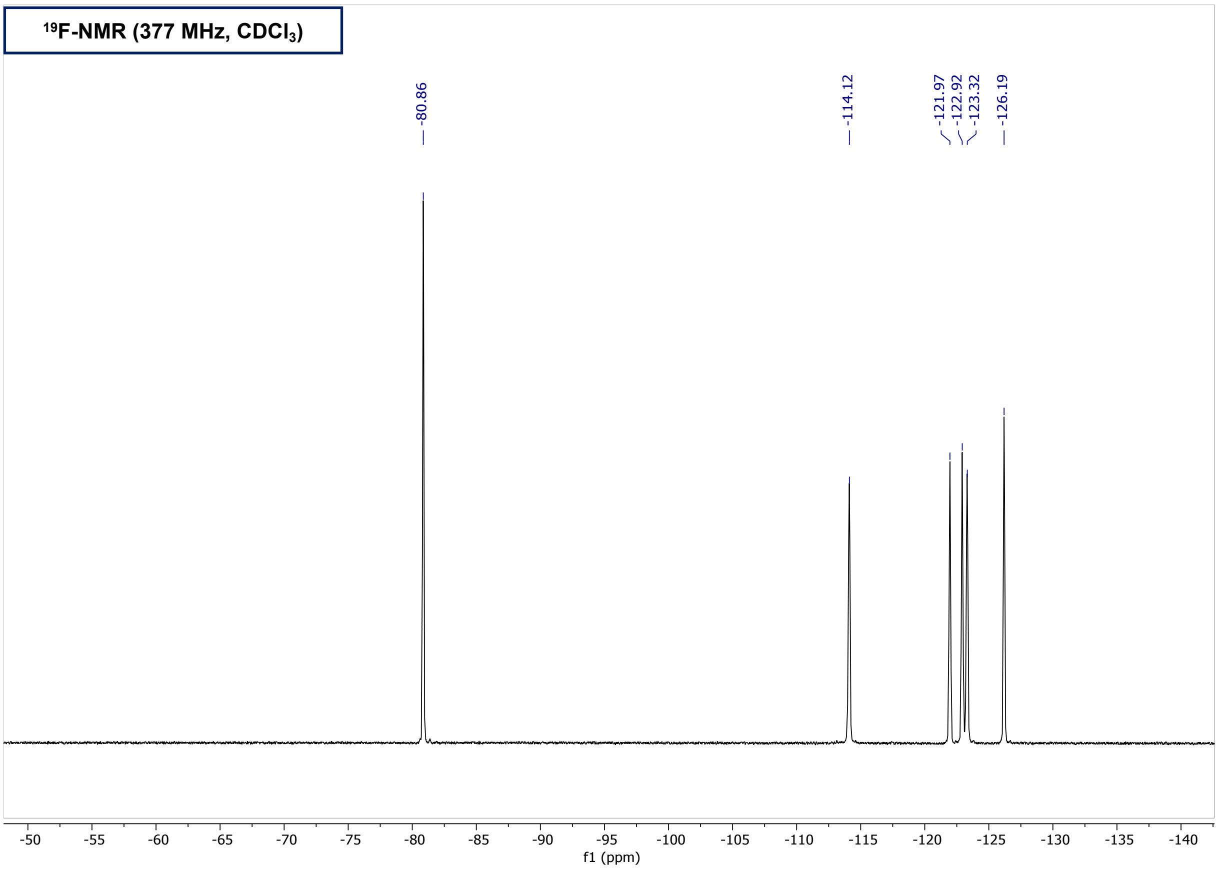

19F-NMR (377 MHz, CDCl3)
-80.86
-114.12
-121.97
-122.92
-123.32
-126.19
-50 -55 -60 -65 -70 -75 -80 -85 -90 -95 -100 -105 -110 -115 -120 -125 -130 -135 -140
f1 (ppm)

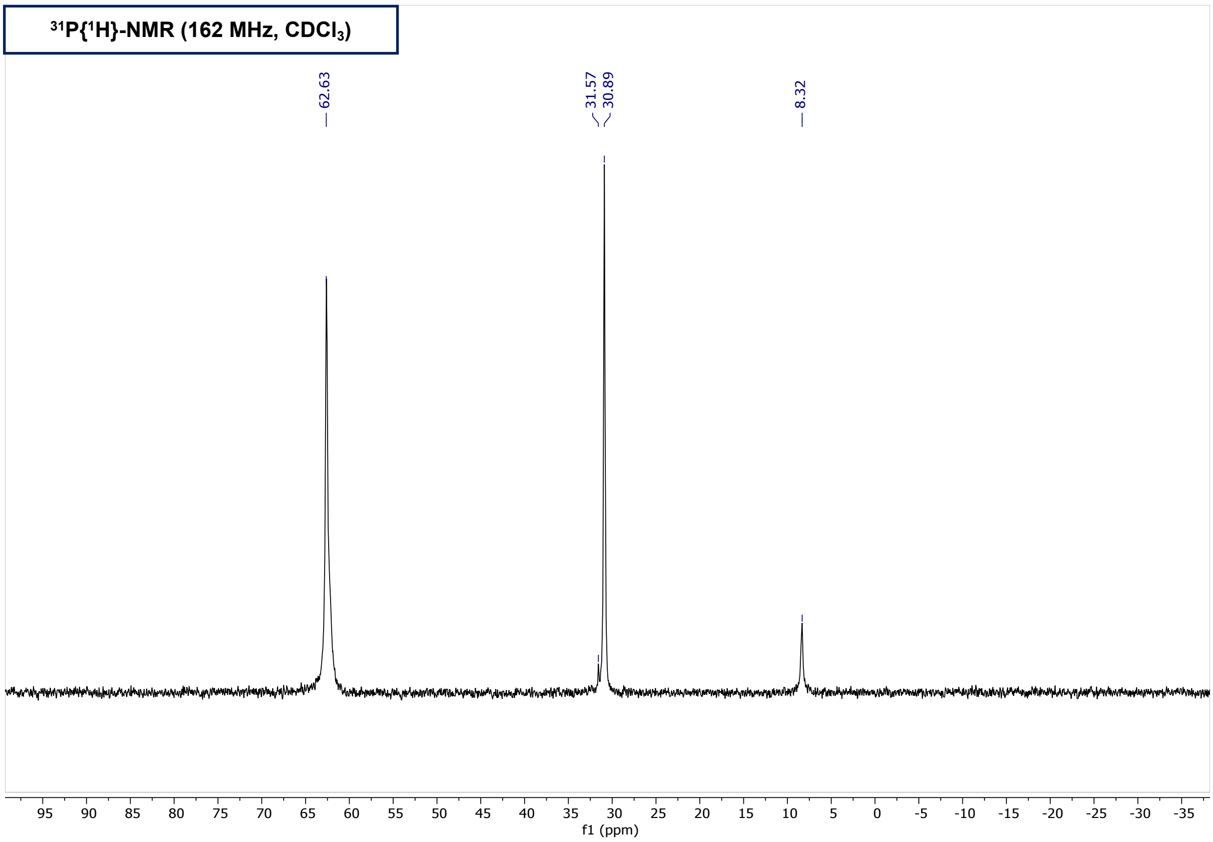

31P{1H}-NMR (162 MHz, CDCl3)
62.63
31.57
30.89
8.32
95 90 85 80 75 70 65 60 55 50 45 40 35 30 25 20 15 10 5 0 -5 -10 -15 -20 -25 -30 -35
f1 (ppm)

G3-mono

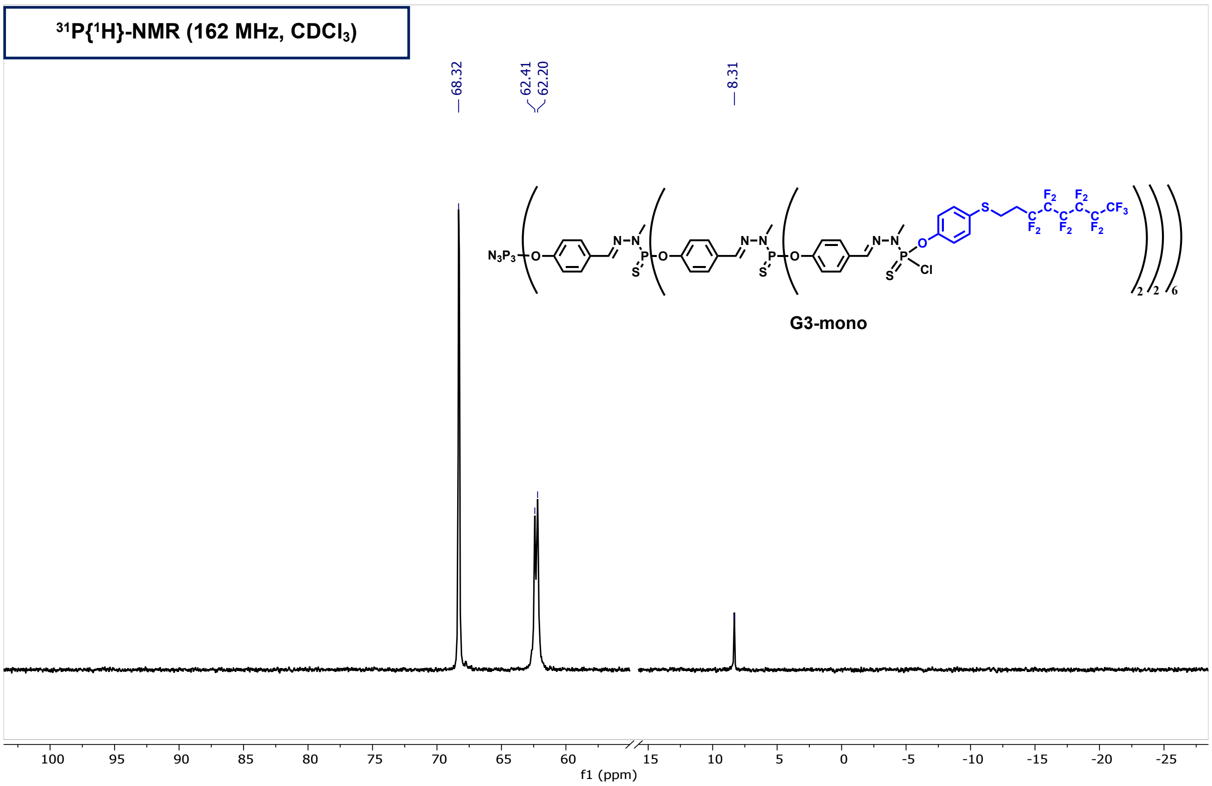

3prc-G3

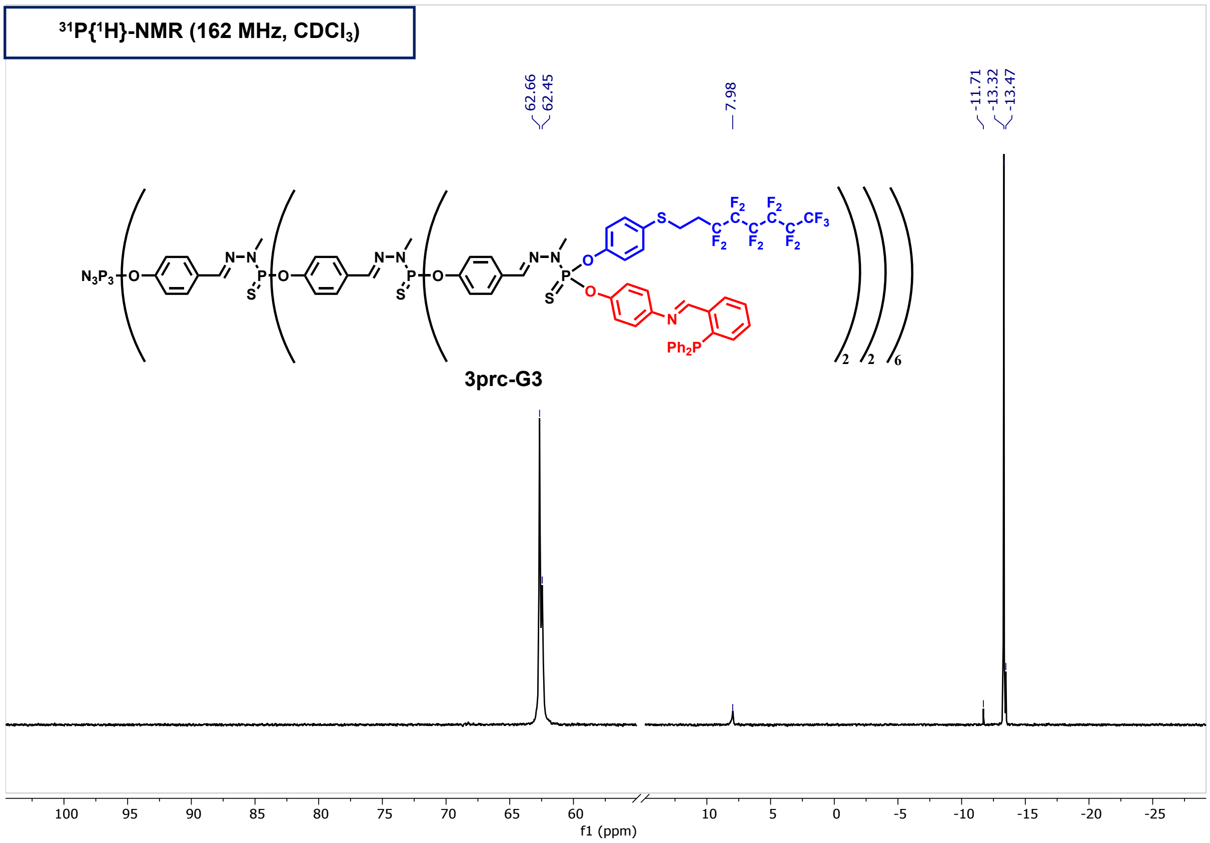

3prc-G3-[Pd]

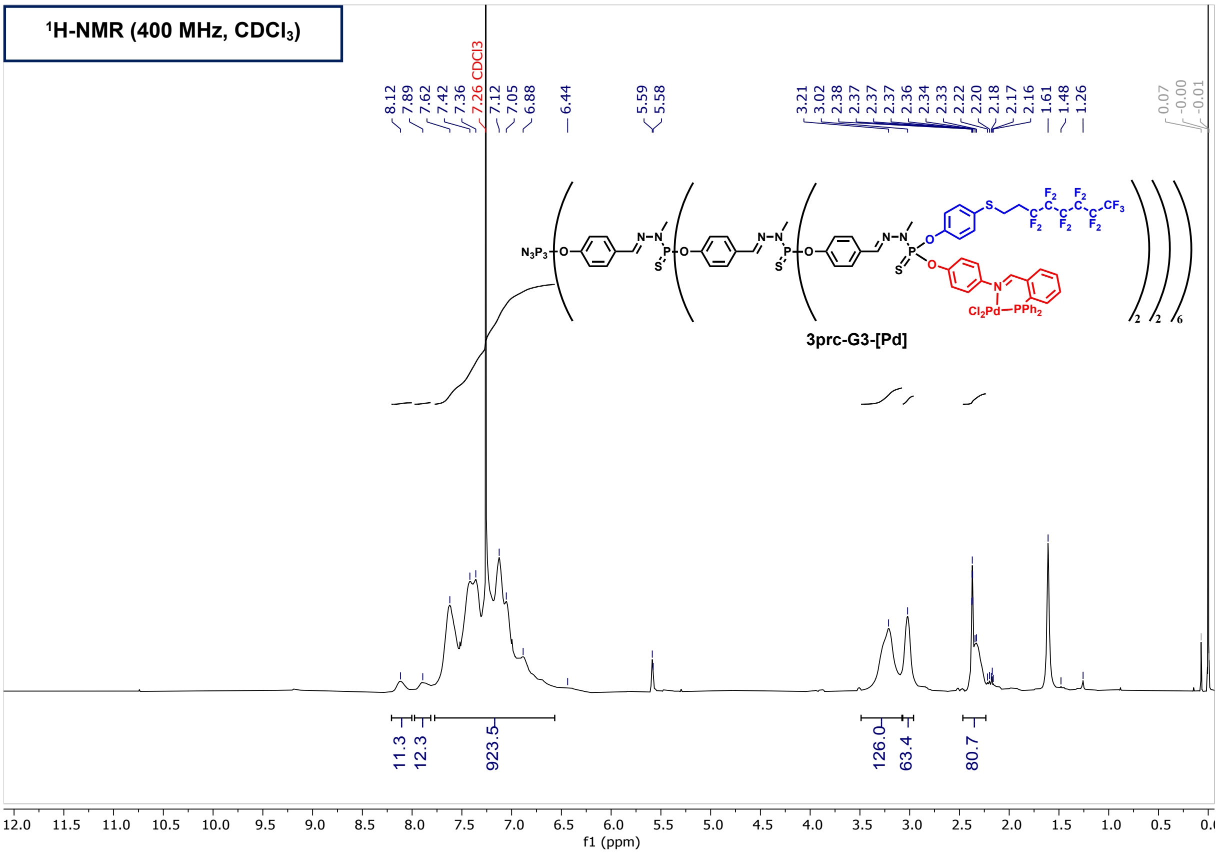

1H-NMR (400 MHz, CDCl3)
3prc-G3-[Pd]
f1 (ppm)

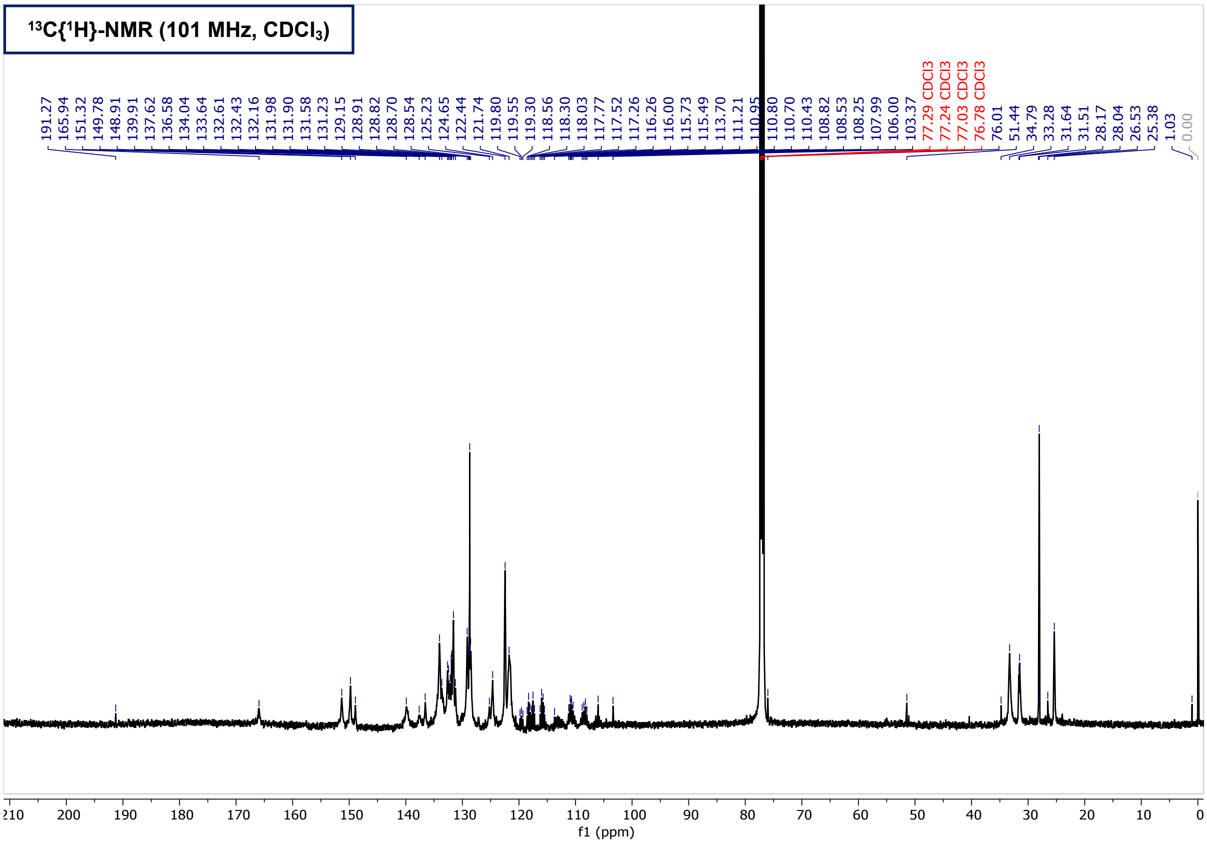

13C{1H}-NMR (101 MHz, CDCl3)
f1 (ppm)

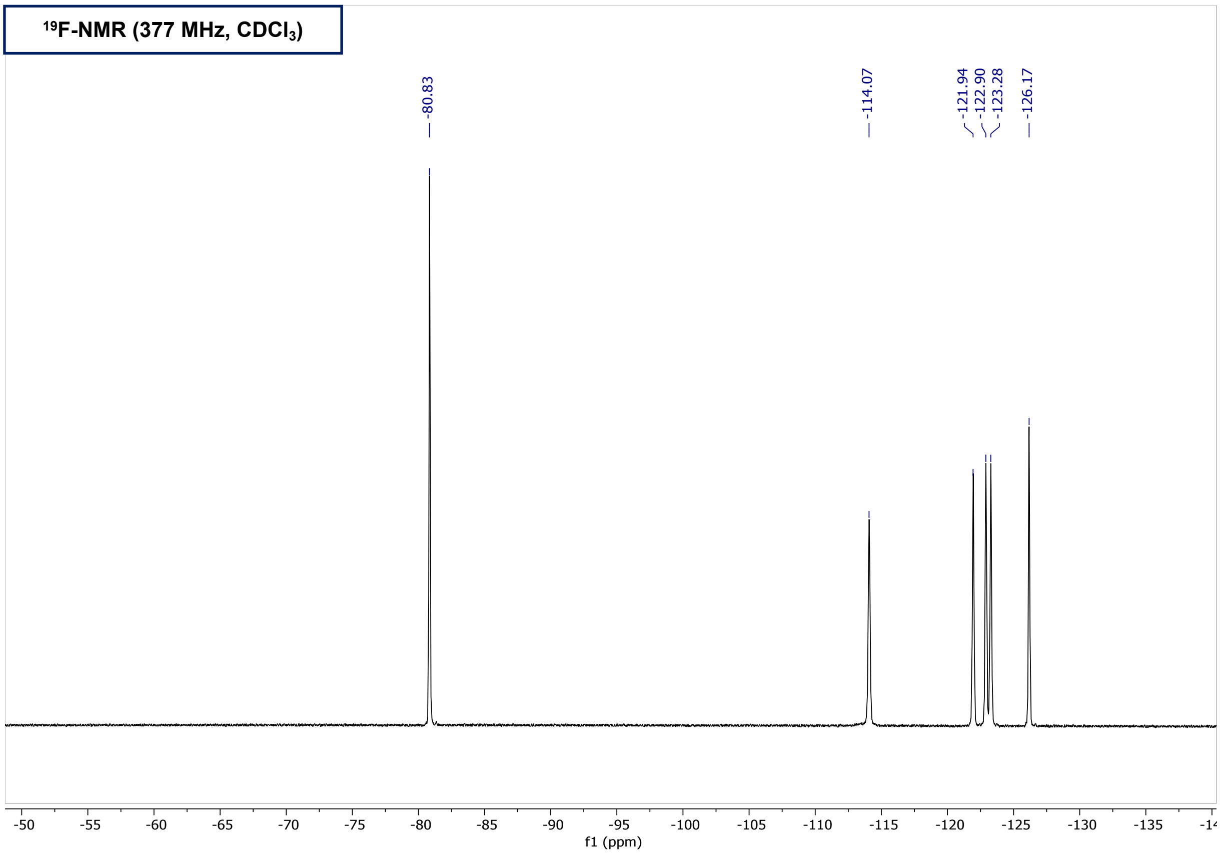

^{19}F-NMR (377 MHz, CDCl$_3$)
-80.83
-114.07
-121.94
-122.90
-123.28
-126.17
f1 (ppm)

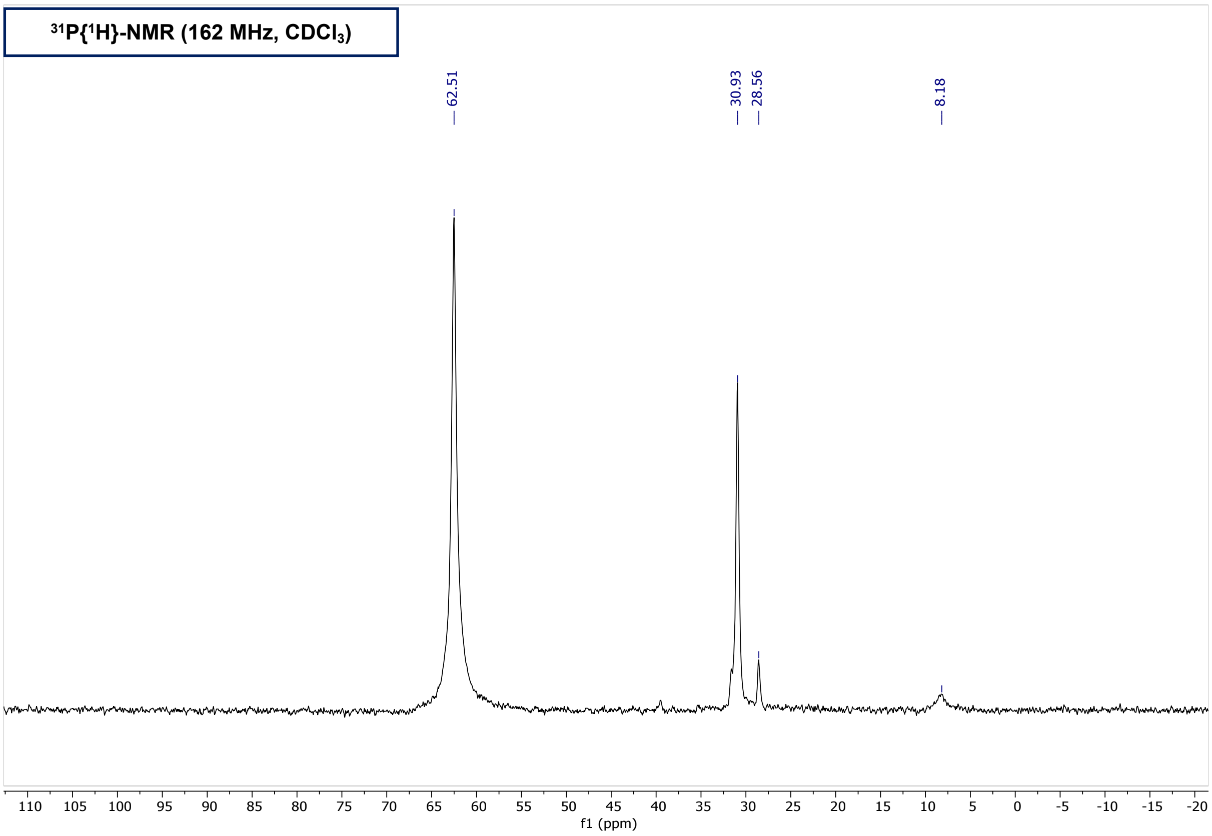

^{31}P{^{1}H}-NMR (162 MHz, CDCl$_3$)
62.51
30.93
28.56
8.18
f1 (ppm)

3rdm-G3

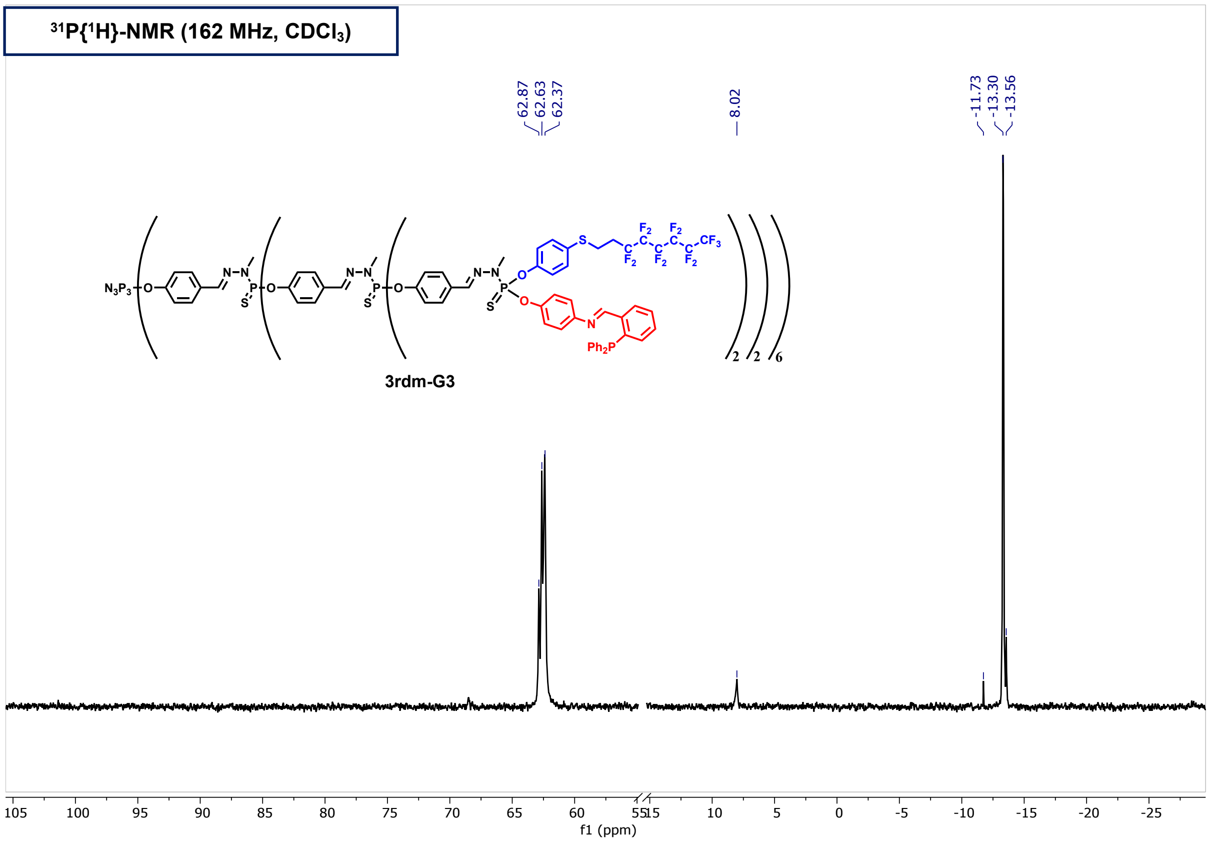

3rdm-G3-[Pd]

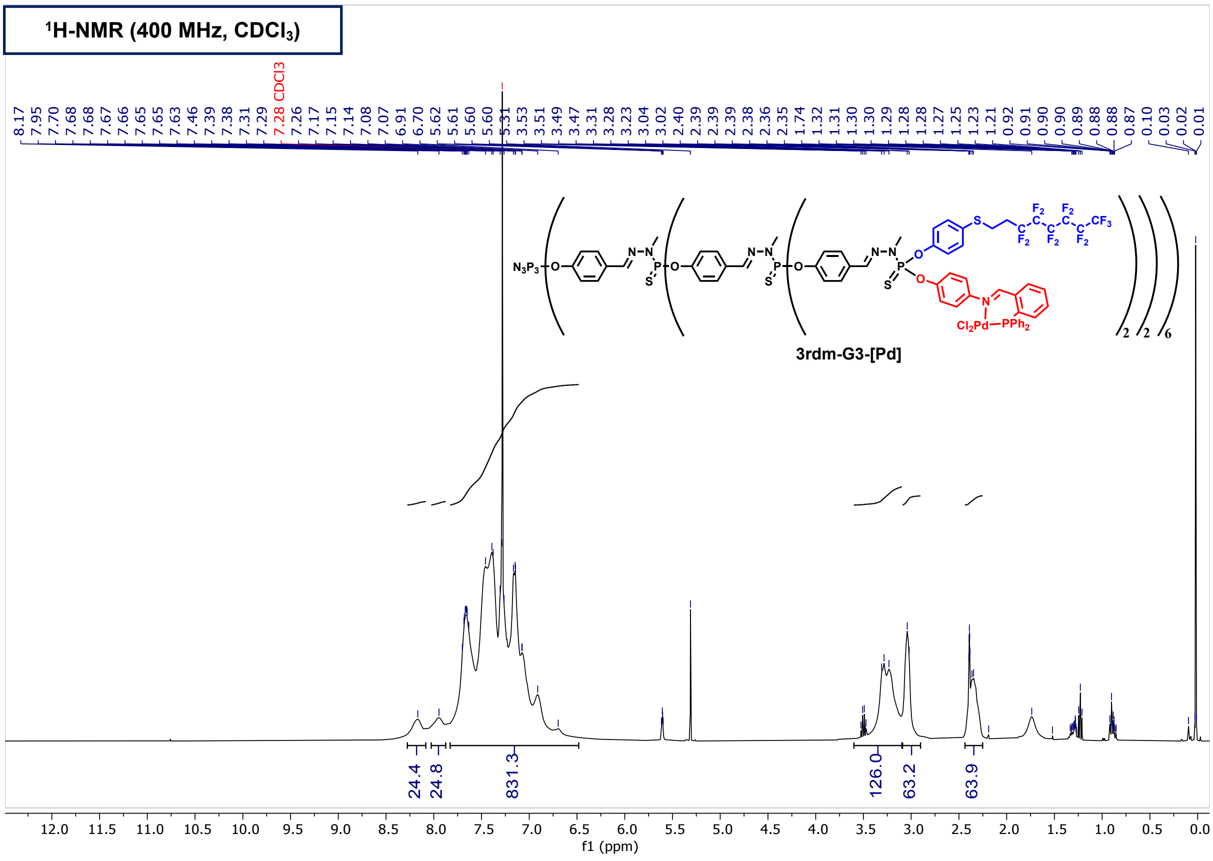

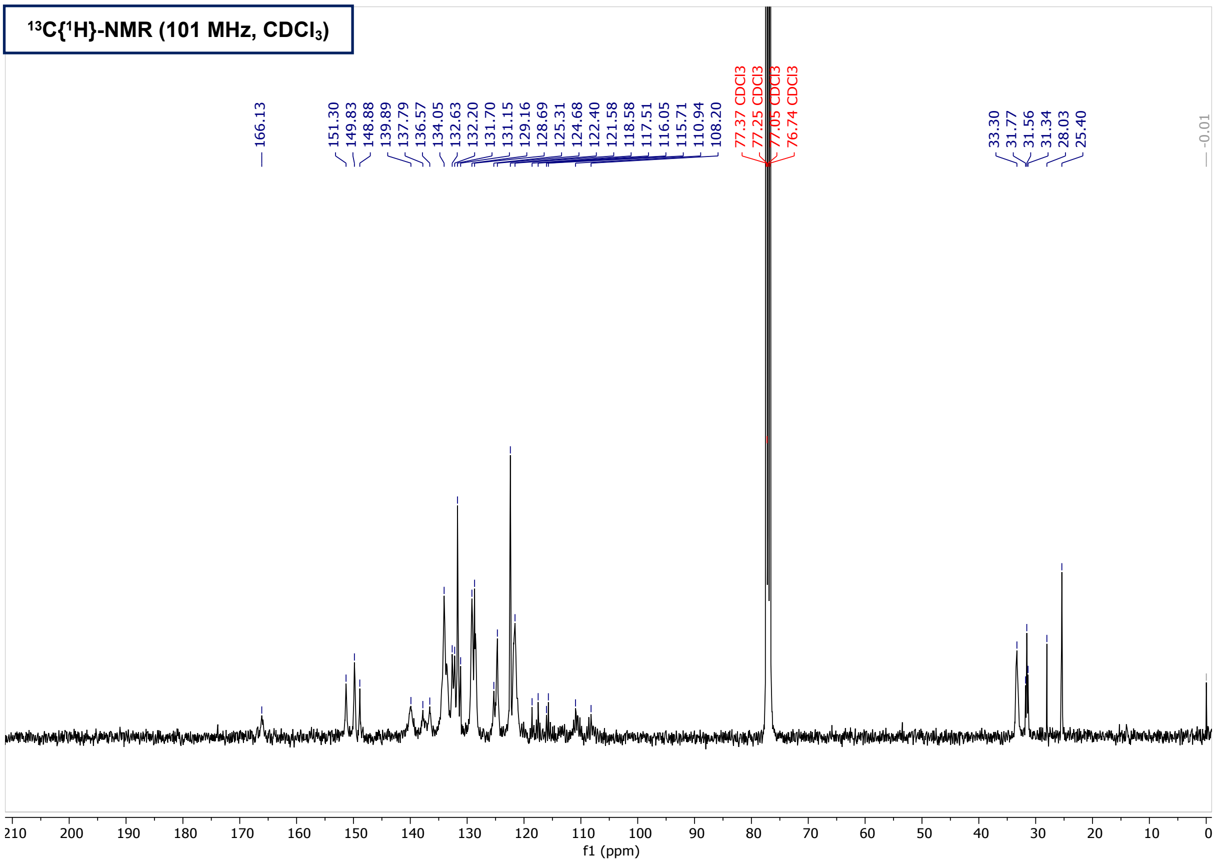

$^{13}C\{^1H\}$-NMR (101 MHz, $CDCl_3$)
166.13
151.30
149.83
148.88
139.89
137.79
136.57
134.05
132.63
132.20
131.70
131.15
129.16
128.69
125.31
124.68
122.40
121.58
118.58
117.51
116.05
115.71
110.94
108.20
77.37 CDCl3
77.25 CDCl3
77.05 CDCl3
76.74 CDCl3
33.30
31.77
31.56
31.34
28.03
25.40
-0.01
f1 (ppm)

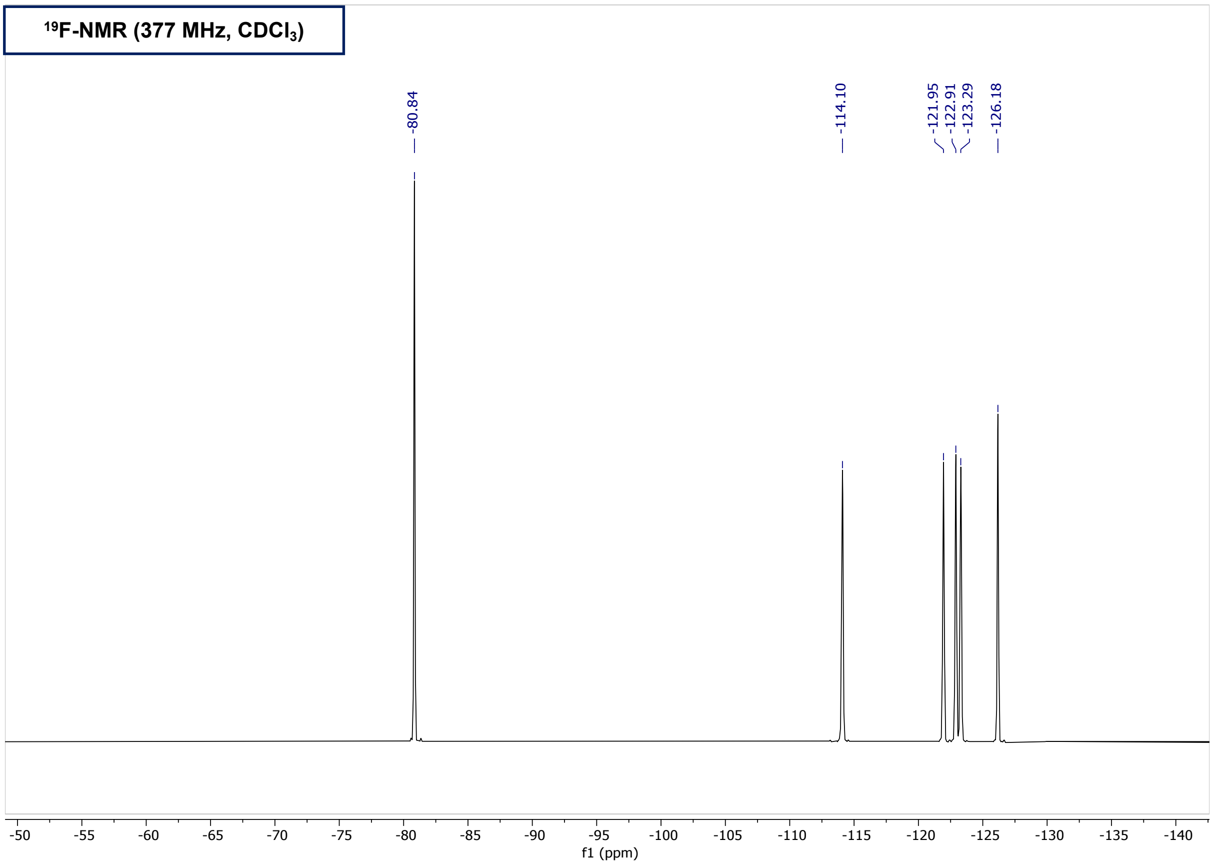

^{19}F-NMR (377 MHz, $CDCl_3$)
-80.84
-114.10
-121.95
-122.91
-123.29
-126.18
f1 (ppm)

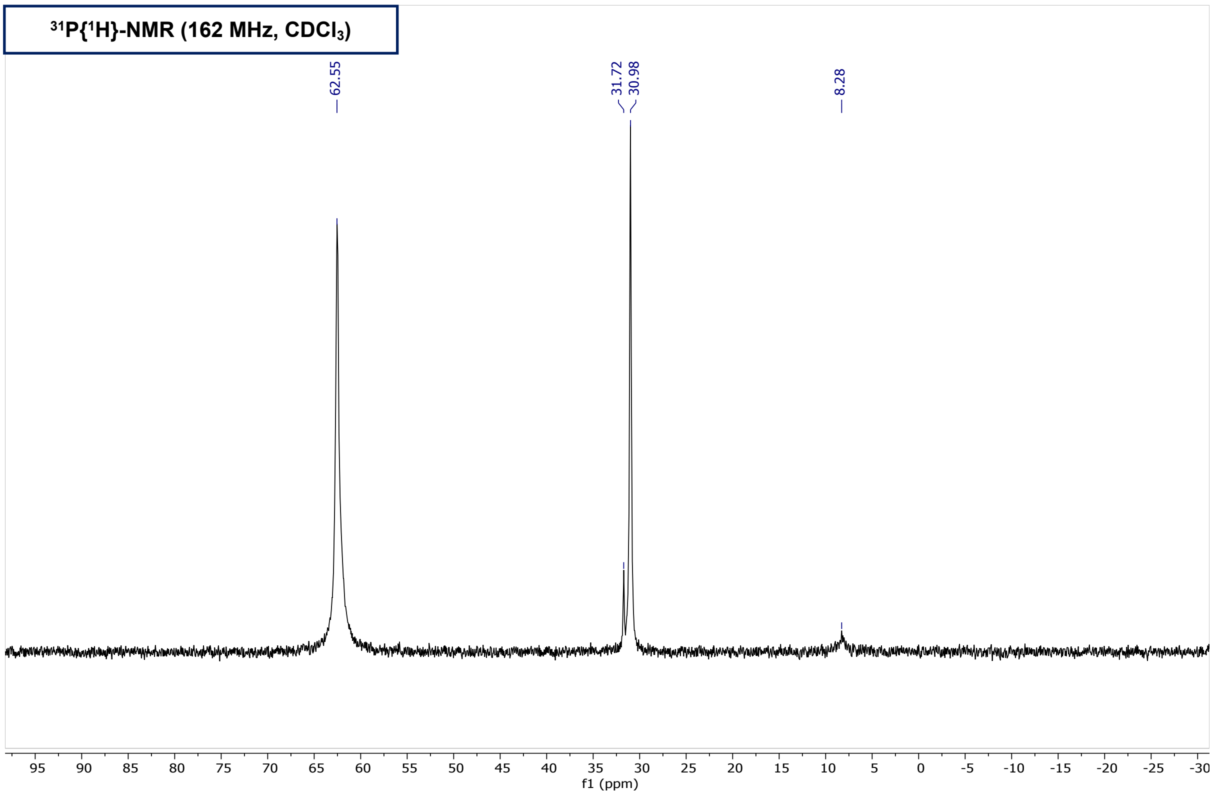

1.5 IR Spectra

3prc-[Pd]

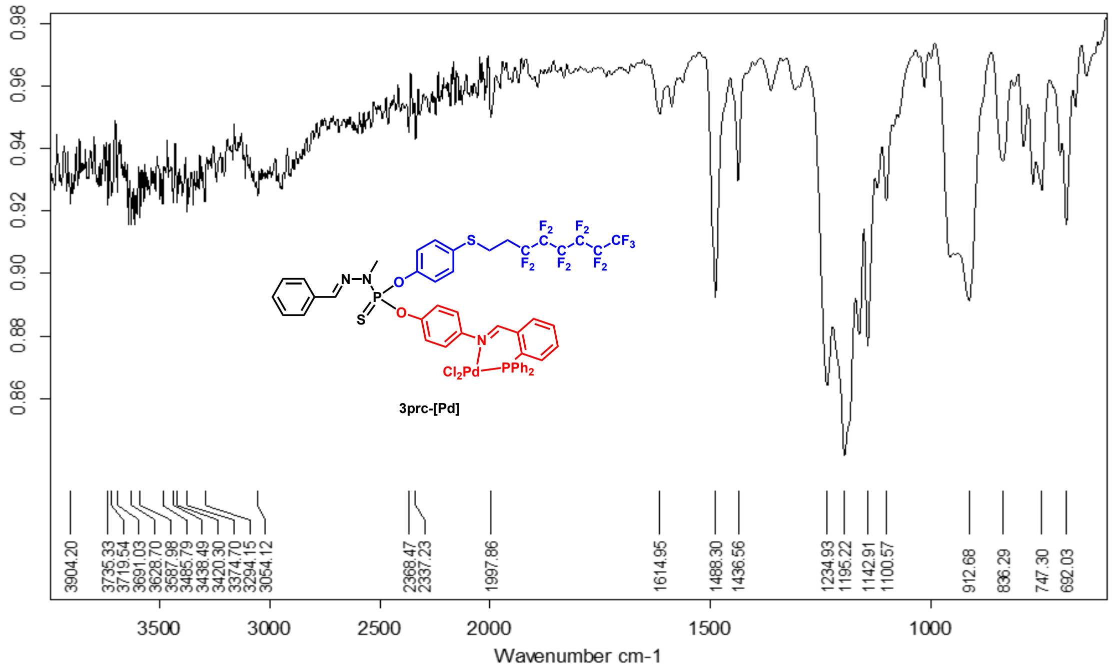

3prc-G1-[Pd]

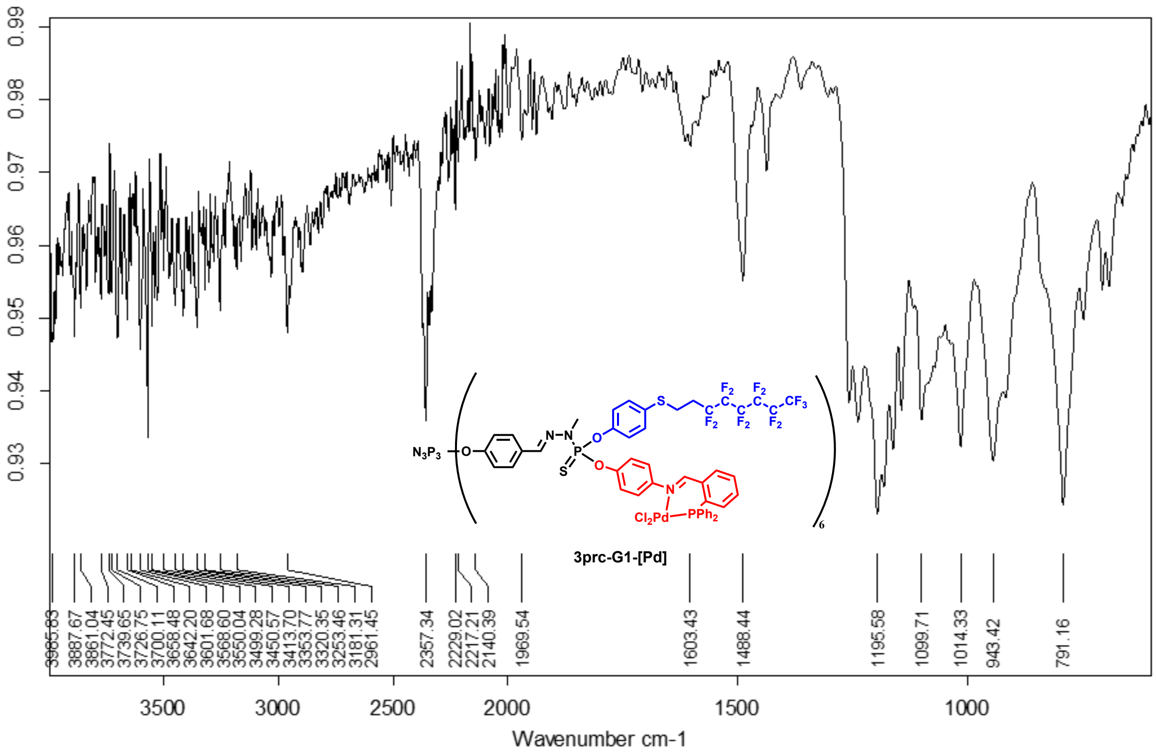

3rdm-G1-[Pd]

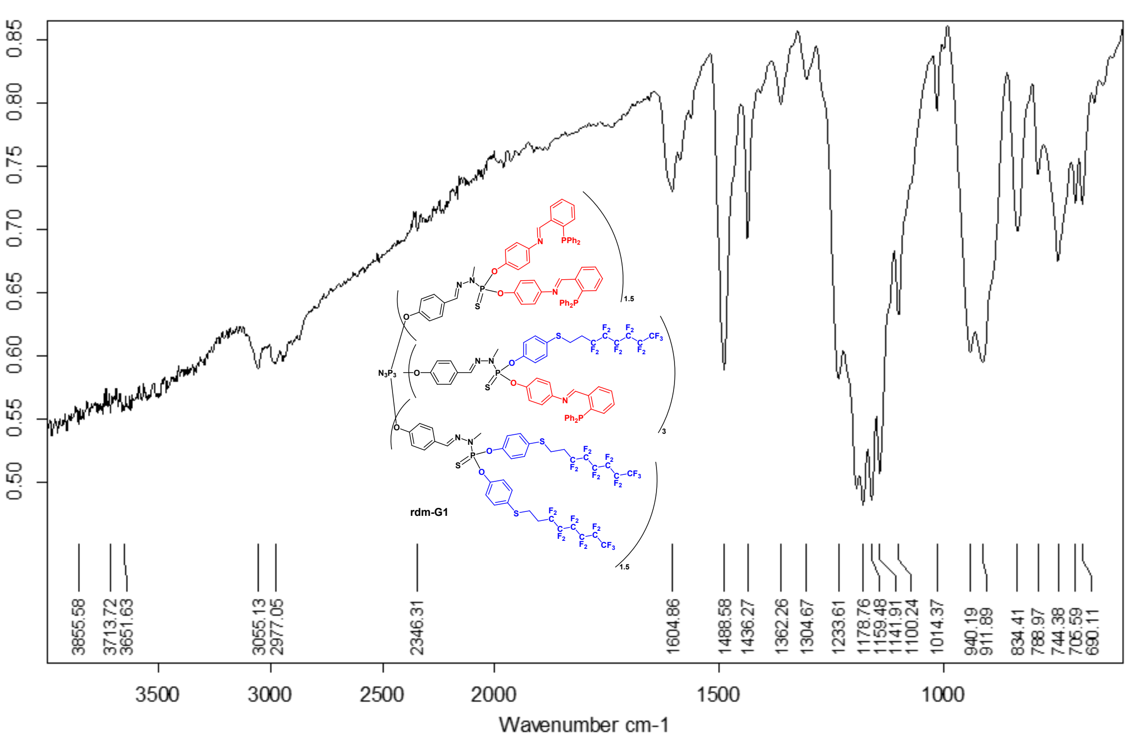

3prc-G2-[Pd]

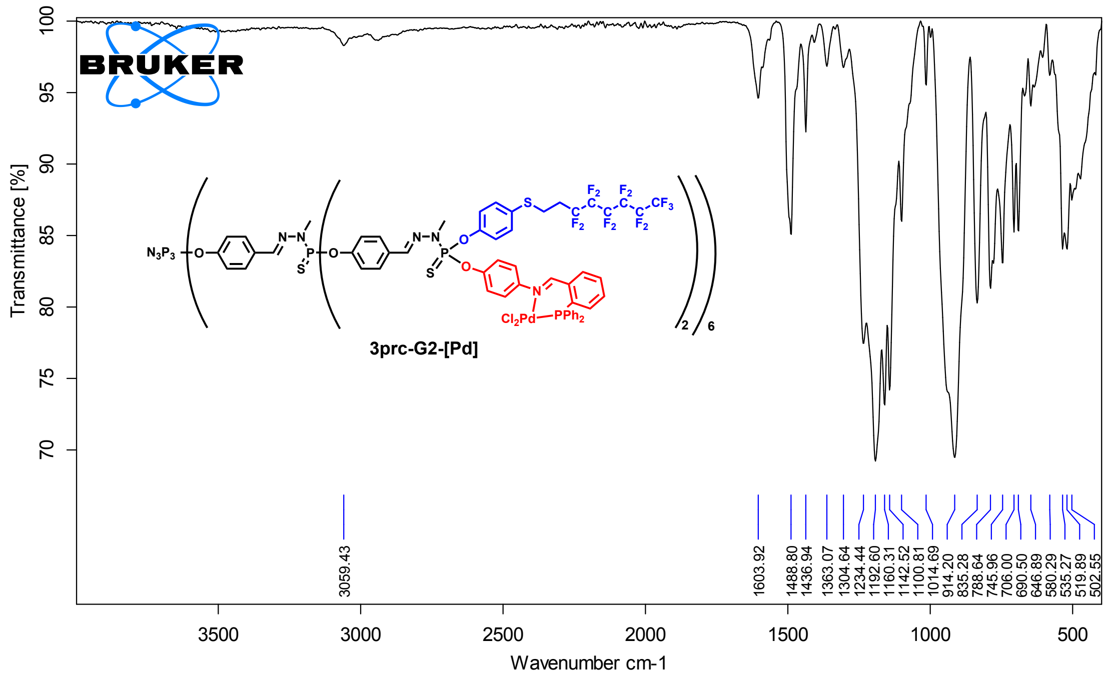

3rdm-G2-[Pd]

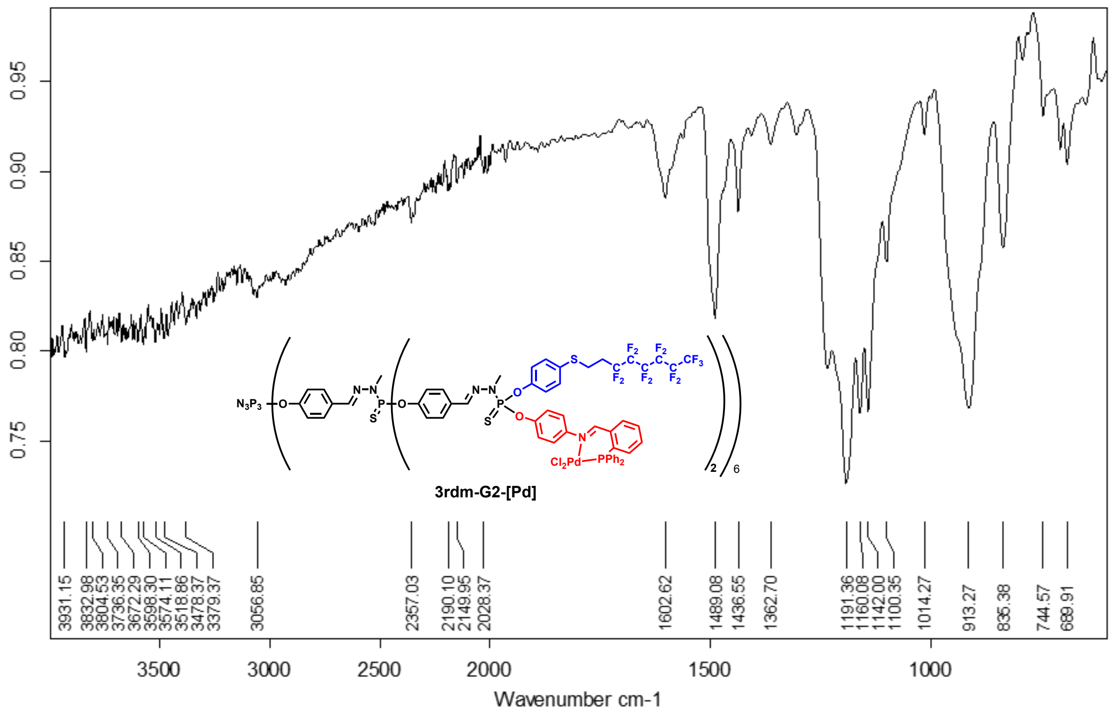

3prc-G3-[Pd]

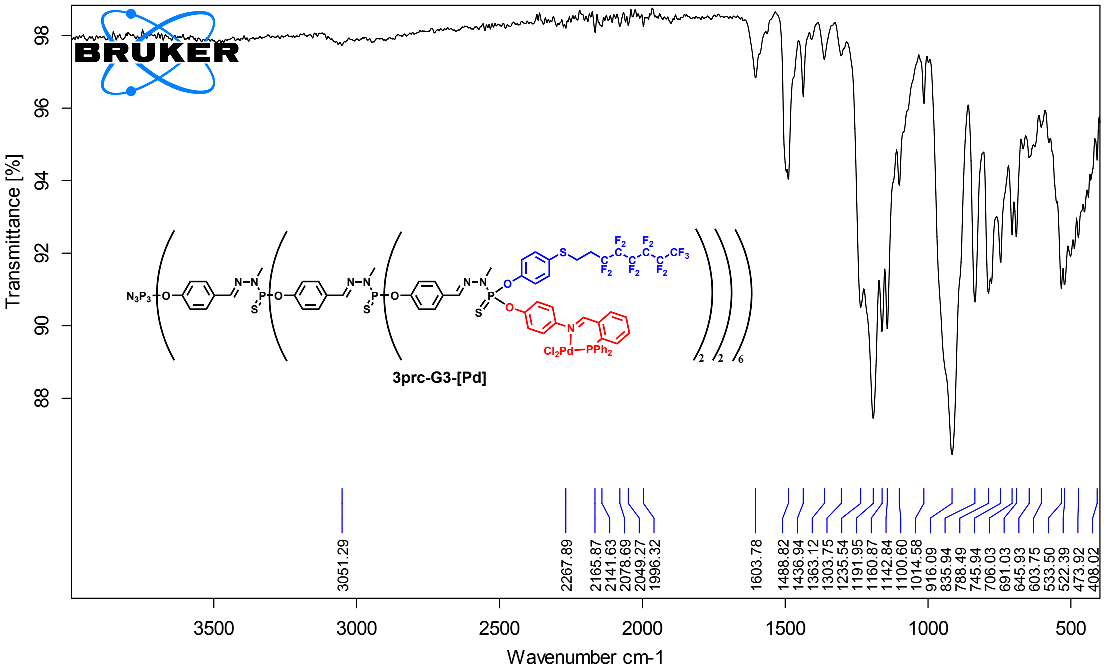

3rdm-G3-[Pd]

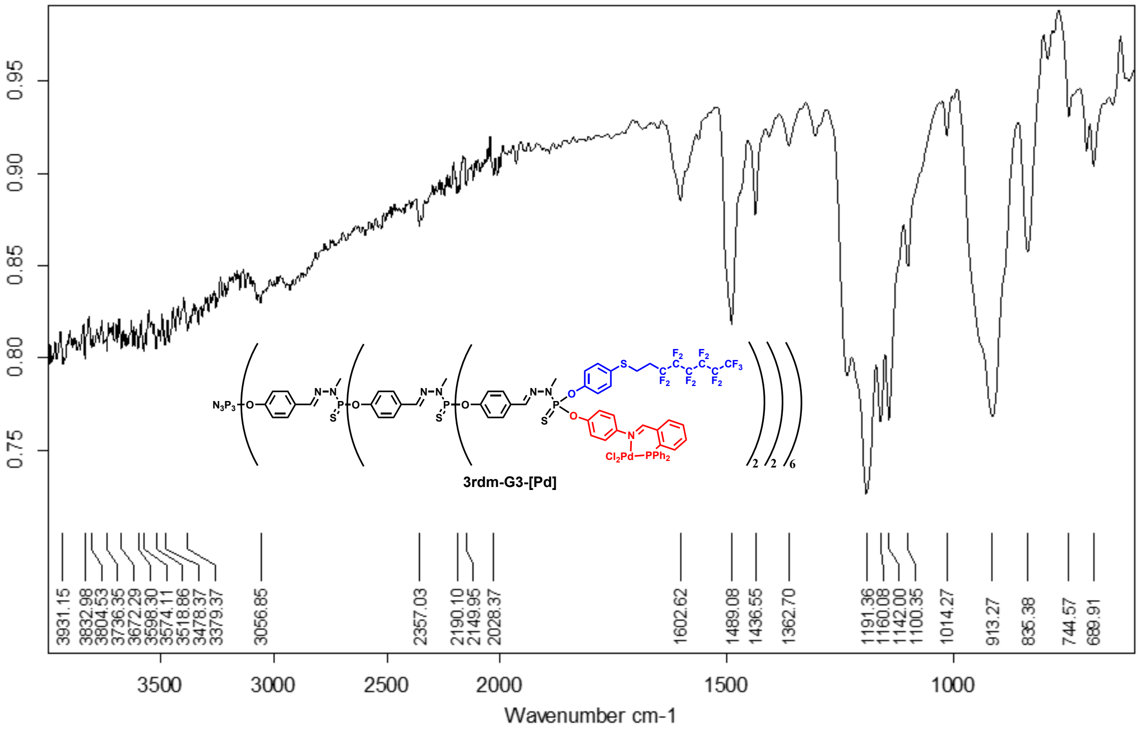

1.6 MS Spectra

3prc-[Pd]

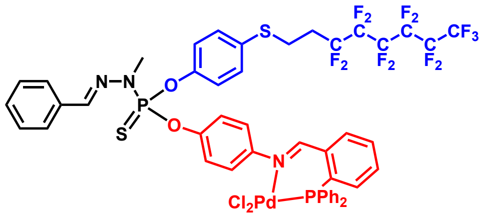

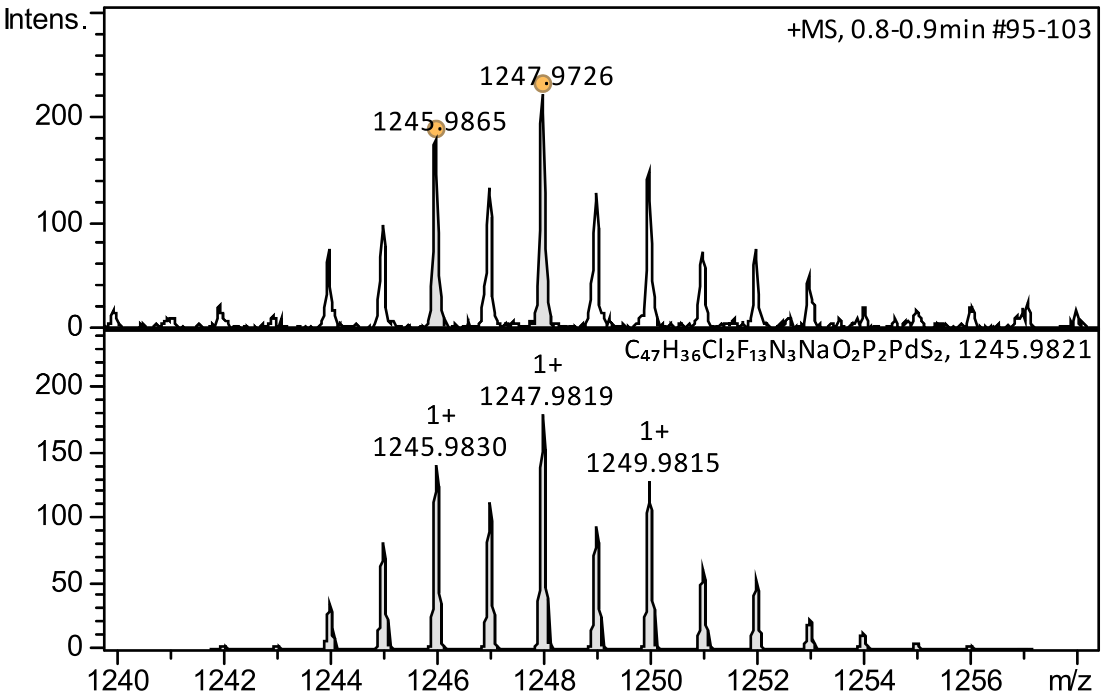

Meas. m/z	#	Ion Formula	m/z	err [mDa]	err [ppm]	mSigma
1245.986511	1	C47H36Cl2F13N3NaO2P2PdS2	1245.982072	-3.5	-2.8	356.5
